# Guidelines for nutrition in adults with head and neck cancer: The American Society for Parenteral and Enteral Nutrition

**DOI:** 10.1002/jpen.70067

**Published:** 2026-03-03

**Authors:** Nicole Kiss, Merran Findlay, Jacqui Frowen, Whitney E. Lewis, Jeannine Mills, Anurag K. Singh, David D. Church, Jacob T. Mey, Sarah Peterson, Kathleen Aguzzi, Sarah Bellini, Maria Paula Villela Coelho, Laura Cordwin, Michael Duffy, Shanna Hager, Manpreet S. Mundi, Michael Owen‐Michaane, Kathleen Price, Heather Stanner, Bridget Storm, Malika Udagedara, Liam McKeever

**Affiliations:** ^1^ Institute for Physical Activity and Nutrition Deakin University Geelong Victoria Australia; ^2^ Maridulu Budyari Gumal (SPHERE) Cancer Clinical Academic Group School of Clinical Medicine, University of New South Wales (UNSW Sydney) Sydney New South Wales Australia; ^3^ Sir Peter MacCallum Department of Oncology The University of Melbourne Melbourne Victoria Australia; ^4^ Bristol Myers Squibb Princeton New Jersey USA; ^5^ Dartmouth Cancer Center Lebanon New Hampshire USA; ^6^ Department of Radiation Medicine Roswell Park Comprehensive Cancer Center Buffalo New York USA; ^7^ Department of Geriatrics University of Arkansas for Medical Sciences Little Rock Arkansas USA; ^8^ Pennington Biomedical Research Center Baton Rouge Louisiana USA; ^9^ Department of Clinical Nutrition Rush University Medical Center Chicago Illinois USA; ^10^ Option Care Health Bannockburn Illinois USA; ^11^ Department of Nutrition, Dietetics, and Food Science Brigham Young University Provo Utah USA; ^12^ Department of Pediatrics Sabará Hospital Infantil São Paulo Brazil; ^13^ Department of Nutrition Services Michigan Medicine Ann Arbor Michigan USA; ^14^ Department of Pediatrics University of Arizona Tucson Arizona USA; ^15^ Department of Pharmacy Select Specialty Hospital Miami Florida USA; ^16^ Division of Endocrinology, Diabetes, Metabolism, and Nutrition, Mayo Clinic Rochester Minnesota USA; ^17^ Division of Preventive Medicine and Nutrition, Columbia University Irving Medical Center New York New York USA; ^18^ American Society for Parenteral and Enteral Nutrition (ASPEN) Silver Spring Maryland USA; ^19^ Metz Culinary Management Dallas Pennsylvania USA; ^20^ Department of Medicine, Division of Gastroenterology University of Toronto Toronto Ontario Canada; ^21^ Department of Clinical Nutrition College of Health Sciences, Rush University Chicago Illinois USA

**Keywords:** appetite stimulants, clinical guidelines, dietitian intervention, dysphagia, energy requirements, enteral nutrition, feeding tubes, head and neck cancer, immunonutrition, interdisciplinary care, malnutrition, nasogastric tube, nutrition assessment, nutrition screening, nutrition support, PEG, postoperative feeding, protein requirements, special nutrients, speech pathology

## Abstract

**Background:**

Head and neck cancer represents the seventh most common cancer diagnosis globally. Maintaining sufficient nutrition is important for preventing malnutrition (undernutrition) and muscle wasting, which contribute to worse outcomes, although many patients are unable to maintain adequate oral intake throughout treatment. This guideline provides practice guidance on nutrition care for patients with head and neck cancer.

**Methods:**

An interdisciplinary team developed key questions and scanned results from a systematic search of the PubMed, EMBASE, CINAHL, and Cochrane Central databases back to 2001. Recommendations were created for key questions concerning timing and duration of nutrition support (early vs delayed enteral nutrition [EN], postoperative feeding, and perioperative dietitian intervention), frequency of dietitian and speech pathology consultations, nutrition screening and assessment, macronutrient requirements, enteral access devices, adjunctive strategies (appetite stimulants and continuation of oral intake alongside EN), specialized nutrients (arginine, glutamine, ω‐3, and immunonutrition), and interdisciplinary care.

**Results:**

Ninety‐two studies were included. Recommendations were made supporting early initiation of EN when oral intake is inadequate, nutrition within 24 h after surgery, weekly consultation with a dietitian during radiotherapy, malnutrition screening and assessment with validated tools, interdisciplinary models of care, dietitian involvement before and after surgery, and when to consider use of specialized nutrients. Recommendations are also provided for protein intake (1.2–1.5 g/kg/day) and energy intake (≥30 kcal/kg/day).

**Conclusion:**

This guideline provides guidance for the nutrition care of patients with head and neck cancer, identifies research gaps, and calls for standardized outcome reporting to further the state of the evidence. This paper was approved by the ASPEN Board of Directors.

## PURPOSE

Head and neck cancer is defined as tumors or cancerous cells arising from the mucosa of the oral cavity, lips, larynx, pharynx, cervical esophagus, nose, sinuses, skin, and salivary glands.^1^, ^2^ Globally, in 2021, head and neck cancers were the seventh most common type of cancer worldwide, accounting for 792,280 new cancer diagnoses and 424,066 deaths.^3^ Patients with head and neck cancer present special nutrition‐related challenges and are at high risk for malnutrition because of difficulties chewing and swallowing, loss of appetite, and other symptoms caused by tumor location and symptomatic response to treatment.^4^ For the purpose of this guideline, malnutrition refers to undernutrition. Guidance is crucial to help interdisciplinary teams administering nutrition care to this key population.

Evidence‐based guidelines for the nutrition management of adult patients with head and neck cancer were first published by the Clinical Oncology Society of Australia (COSA) in 2011 and have since been updated and maintained as a living guideline.^5^ The COSA guidelines are a comprehensive synthesis of the literature addressing the continuum of care from diagnosis through survivorship and palliative care and encompassing all study designs from randomized control trials (RCTs) to cross‐sectional designs, as well as studies that included other high nutrition risk cancer populations. This American Society for Parenteral and Enteral Nutrition (ASPEN) guideline departs from the COSA effort by restricting to study designs capable of providing causal inference (RCTs and quasi‐experimental designs) and by tightening population restrictions to exclusively include head and neck cancer populations to form recommendations under these strict criteria. Additionally, this guideline is expanded to include new questions reflecting areas of current clinical interest and multidisciplinary team care.

This clinical guideline's objective is to provide practice guidance for all clinicians on the nutrition care of adult patients with head and neck cancer via a systematic review and grading of the scientific literature, and a modified Delphi approach to facilitate expert consensus. The complete guideline recommendations are presented in Table 1.

**Table 1:** Guideline questions, evidence grades, recommendations.

**1a: In adult patients with head and neck cancer receiving chemoradiation or radiation, does earlier EN vs later EN impact clinical outcomes?** *Recommendation*: In adults with head and neck cancer planning for or receiving radiotherapy with or without chemotherapy or other systemic therapy, we suggest initiation of enteral feeding where there is clinical evidence that nutrition intake or status is compromised, despite other strategies (oral nutrition supplements, food fortification) having been attempted. This should be individualized to oral nutrition intake, symptom burden, and nutrition status while considering individual treatment plans and clinical, psychosocial, and socioeconomic status. **Certainty of evidence:** Moderate **Strength:** Strong
**1b: In adult patients with head and neck cancer, does longer postoperative nutrition (enteral or oral nutrition supplements) vs shorter duration of nutrition support impact clinical outcomes?** *Recommendation*: In adults with head and neck cancer, we suggest early commencement (within 24 h) of postoperative nutrition intake (EN, oral, or otherwise) to meet estimated nutrition requirements. Early initiation of oral intake should occur in consultation with the surgical team, dietitian, and speech pathologist. To meet nutrition requirements, oral intake should be increased gradually, and supplemental enteral feeding should be maintained until sufficient oral intake is established. Early initiation of oral intake may be inappropriate in certain situations, making consultation with surgical, speech, and nutrition teams critical. **Certainty of evidence:** Very low **Strength:** Weak
**1c: In adult patients with head and neck cancer, does increasing the frequency of dietetic intervention vs standard care during chemoradiation or radiation and up to 3 months posttreatment impact clinical outcomes?** *Recommendation*: In adults with head and neck cancer receiving radiotherapy with or without chemotherapy or other systemic therapy, we recommend weekly consultation with a dietitian during treatment and fortnightly (every 2 weeks) for up to 6 weeks after treatment to maintain nutrition status and quality of life while preventing unplanned hospital admissions and early cessation of treatment. **Quality of evidence:** High **Strength:** Strong
**1d. In adult patients with head and neck cancer, does longer preoperative and postoperative intervention by a dietitian compared with shorter intervention duration impact clinical outcomes?** *Recommendation*: In adults with head and neck cancer, we recommend that the duration of dietitian intervention preoperatively and postoperatively should be individualized according to the patient's nutrition status; swallowing function; symptom burden; and clinical, psychosocial, and socioeconomic status. Intervention should continue while there remains a risk to nutrition status from nutrition‐impact symptoms, reduced dietary intake, continued indication for nutrition support, and/or other factors relating to nutrition risk. Patients undergoing certain surgical procedures, such as those with extensive surgical resections (eg, free flap reconstructions) or with dysphagia, may require dietetic support for a longer duration. **Quality of evidence:** Very low/expert opinion **Strength:** Strong
**2a. In adult patients aged ≥16 years with head and neck cancer receiving any treatment modality, does nutrition screening vs not screening impact clinical outcomes?** *Recommendation*: In adults with head and neck cancer, we recommend that all patients be screened for malnutrition using a validated tool at their first presentation to the healthcare facility and regularly throughout treatment and recovery to facilitate timely referral for nutrition intervention. In certain patient subgroups who are at higher risk of malnutrition, screening may be bypassed if processes are established for automatic referral to a dietitian. **Certainty of evidence:** Very low/expert opinion **Strength:** Strong
**2b. In adult patients with head and neck cancer receiving any treatment modality, does nutrition assessment vs no nutrition assessment impact clinical outcomes?** *Recommendation*: In adults with head and neck cancer, we recommend that patients undergo a comprehensive nutrition assessment if they have been screened and found to be at risk of malnutrition, if they are automatically referred through established protocols because of high malnutrition risk, or if they present with an enteral access device, either planned or already in situ. We further recommend this assessment be performed by a dietitian or other qualified nutrition professional using a tool that has been validated in the oncology population (eg, PG‐SGA or SGA). **Certainty of evidence:** Very low/expert opinion **Strength:** Strong
**3a. In adult patients with head and neck cancer receiving any treatment modality, does intensive nutrition therapy designed to meet current recommendations for protein intake vs standard care impact clinical outcomes?** *Recommendation*: In adults with head and neck cancer receiving any modality of treatment, we recommend a protein intake of 1.2–1.5 g/kg/day, which would meet the needs of most patients with head and neck cancer. This may be met and maintained through one or a combination of oral intake, oral nutrition supplements, or EN to meet protein requirements and should be tailored to symptom burden and nutrition status while considering individual treatment plans, clinical, psychosocial, and socioeconomic status. **Quality of Evidence:** Very low/expert opinion **Strength:** Strong
**3b. In adult patients with head and neck cancer receiving any treatment modality, does intensive nutrition therapy designed to meet current recommendations for energy intake vs standard care impact clinical outcomes?** *Recommendation*: In adults with head and neck cancer receiving any modality of treatment, we recommend an energy intake of at least 30 kcal/kg/day. This may be met and maintained through one or a combination of oral intake, oral nutrition supplements, or EN to meet energy requirements and should be tailored to symptom burden and nutrition status while considering individual treatment plans, clinical, psychosocial, and socioeconomic status. Nutrition status should be monitored regularly to determine if energy intake is sufficient, noting that sufficient energy intake is also important to ensure protein intake is used for the preservation of muscle mass. **Certainty of evidence:** High **Strength:** Strong
**4a. In adult patients with head and neck cancer receiving any treatment modality, does estimating protein requirements based on an alternate body weight or composition vs standard care (actual weight) impact clinical outcomes?** *Recommendation*: In adults with head and neck cancer receiving any treatment modality, because of insufficient evidence at present to demonstrate a benefit from individualizing protein requirements based on body composition, we suggest estimating protein requirements based on actual body weight. However, the risk of overestimating protein requirements in patients with obesity is higher. An acceptable solution to this may be to use the higher end of ideal body weight or to use actual body weight, while using clinical judgment to determine whether the resulting target protein amount is achievable. We recommend ongoing monitoring of nutrition intake alongside nutrition status, muscle mass, muscle strength, and physical performance as an indication of adequacy of protein intake. **Quality of evidence:** Very low/expert opinion **Strength:** Weak
**4b. In adult patients with head and neck cancer receiving any treatment modality does estimating energy requirements based on an alternate body weight or composition vs standard care (actual weight) impact clinical outcomes?** *Recommendation*: In adults with head and neck cancer receiving any treatment modality, we suggest estimating energy requirements based on actual body weight because of insufficient evidence at present to demonstrate a benefit from individualizing energy requirements based on body composition. However, the risk of overestimating energy requirements in patients with obesity is higher. In this situation, either ideal body weight or actual body weight may be used with clinical judgment to determine whether the resulting target energy requirement is achievable. We recommend ongoing monitoring of nutrition intake alongside weight, nutrition status, muscle mass, muscle strength, and physical performance as an indication of adequacy of energy intake. **Quality of evidence:** Very low/expert opinion **Strength:** Weak
**5. In adult patients with head and neck cancer receiving any treatment modality, does gastrostomy feeding (via PEG or RIG) vs NGT feeding impact clinical outcomes?** *Recommendation*: In adults with head and neck cancer receiving any treatment modality, we suggest that the decision to place a PEG or RIG tube vs an NGT is made through discussion among interdisciplinary team members, including a dietitian or other member with nutrition training. The decision regarding the type of enteral access device should be based on the clinical situation (including tumor location and stage), symptom burden (especially preexisting dysphagia), treatment plan, psychosocial situation, and the anticipated duration of enteral feeding. If EN is indicated, feeding via PEG/RIG may be more appropriate when anticipated for longer durations (commonly >4–6 weeks); otherwise, an NGT should be considered. **Quality of evidence:** Moderate **Strength:** Weak
**6. In adult patients with head and neck cancer receiving any treatment modality, does more frequent speech pathology intervention compared with standard of care impact clinical outcomes?** *Recommendation*: In adults with head and neck cancer receiving any treatment modality, we recommend consultation by a speech pathologist before treatment (surgery or radiotherapy with or without chemotherapy) for baseline assessment and education if the treatment is likely to affect swallowing function, or in the case of preexisting dysphagia. We recommend that the frequency of consultation by a speech pathologist during and after radiotherapy (with or without chemotherapy) and after surgery be guided by the treatment plan, as well as the severity of dysphagia and other treatment toxicities. Additional considerations include clinical, psychosocial, and socioeconomic status. Interventions should be tailored to reduce dysphagia risk, minimize malnutrition, and improve quality of life. These interventions may include the maintenance of oral intake throughout radiotherapy (if safe to do so), prophylactic or therapeutic swallowing exercises, texture modification, swallowing maneuvers, compensatory strategies, and education. **Quality of evidence:** Moderate **Strength:** Strong
**7. In adult patients with head and neck cancer undergoing any treatment modality, does a multidisciplinary approach to nutrition management vs standard care impact clinical outcomes?** *Recommendation*: In adults with head and neck cancer, we recommend an interdisciplinary approach to nutrition management. An interdisciplinary approach should involve collaboration between health professionals with the expertise to manage any symptom or issue that is affecting or anticipated to affect the patient's nutrition intake or nutrition status. We recommend that the core team for nutrition management include dietitians, nurses, pharmacists, physicians, and speech pathologists. Additional members may include dental professionals, physical therapists, psychologists, and social workers. **Quality of evidence:** Moderate **Strength:** Strong
**8. In adult patients with head and neck cancer receiving any treatment modality, does a pharmaceutical appetite stimulant compared with no pharmaceutical appetite stimulant impact clinical outcomes?** *Recommendation*: In adults with head and neck cancer who are experiencing anorexia and receiving any treatment modality, we suggest dietary counseling (including oral nutrition supplements or enteral feeding) and management of other symptoms that are affecting oral intake as first‐line strategies to address anorexia and improve nutrition intake. Otherwise, a pharmaceutical appetite stimulant may be considered for short‐term use where clinically appropriate. In conjunction with the medical team and dietitian, this decision should ideally include discussion with a pharmacist specializing in oncology. **Quality of evidence:** Very low/expert opinion **Strength:** Weak
**9. In adult patients with head and neck cancer receiving chemoradiation or radiation, does continuing oral intake (if tolerated) after the initiation of EN compared with not continuing oral intake impact clinical outcomes?** *Recommendation*: In adults with head and neck cancer who have commenced EN and who can safely continue oral intake per consult with a speech pathologist, we suggest that continuing any degree of oral intake may be beneficial for maintaining swallow function. The amount, type, and texture of the oral intake will depend on swallow safety and treatment toxicities. The volume and timing of enteral feeding should be adjusted according to what is consumed orally to optimize the opportunity for the patient to continue oral intake while also ensuring that nutrition requirements are met. **Quality of evidence:** Very low/expert opinion **Strength:** Weak
**10. In adult patients with head and neck cancer receiving any treatment modality, does use of special‐purpose nutrients compared with not using special‐purpose nutrients impact clinical outcomes?** *Recommendation*: ***Arginine*** Given the limited evidence on progression‐free and overall survival and some evidence of benefit for decreased fistula development and length of stay in adults with head and neck cancer, we suggest that using arginine‐supplemented nutrition may be acceptable at the discretion of the interdisciplinary team. **Certainty of evidence:** Low **Strength:** Weak ***Glutamine*** Oral/enteral glutamine has been shown to reduce the severity of oral mucositis, with the potential to reduce other treatment toxicities and hospitalization as well as improve treatment completion. We therefore suggest that the use of oral/enteral glutamine in patients with head and neck cancer may be acceptable at the discretion of the interdisciplinary team. Intravenous glutamine is more controversial because of one small study that reported increased mortality in the patients receiving intravenous glutamine and recent preclinical trials suggesting mechanisms through which glutamine may contribute to tumor growth and treatment resistance. For this reason, we suggest not adding parenteral glutamine to standard nutrition therapy in patients with head and neck cancer until further research becomes available to confirm its safety. **Certainty of evidence:** High **Strength:** Weak ***ω‐3*** Given the inconsistent evidence for benefit but no evidence of significant harms in patients with head and neck cancer, we suggest that ω‐3–supplemented nutrition is unlikely to be harmful and may be used or not at the discretion of the interdisciplinary team. **Certainty of evidence:** Low **Strength:** Weak ***Combined special‐purpose nutrients/immunonutrition*** In patients with head and neck cancer, given the inconsistent evidence for benefit but no evidence of significant harms, we suggest that combined special‐purpose nutrient or immunonutrition‐supplemented formulas are unlikely to be harmful and may be used or not at the discretion of the interdisciplinary team. **Certainty of evidence:** Very low **Strength:** Weak

*Note*: Clinical outcomes are progression‐free survival, overall survival, nutrition intake, time to transition to full oral diet, nutrition status, weight, muscle mass, sarcopenia, myeosteatosis, global quality of life, fatigue, return to work, performance status, length of stay, surgical complications, and hospital readmissions.

Abbreviations: EN, enteral nutrition; NGT, nasogastric tube; PEG, percutaneous endoscopic gastrostomy; PG‐SGA, Patient‐Generated Subjective Global Assessment; RIG, radiologically inserted gastrostomy; SGA, Subjective Global Assessment.

Recommendations in this guideline do not constitute medical or other professional advice and should not be taken as such. To the extent that the information published herein may be used to assist in the care of patients, the primary component of quality medical care is the result of the professional judgment of the healthcare professionals providing care. The information presented here is not a substitute or replacement for the exercise of professional judgment by healthcare professionals; rather, it is intended to supplement professional training and judgment. Circumstances and patient specifics in clinical settings may require actions different from those recommended in this document; in those cases, the judgment of the treating professionals should prevail. Use of this information does not in any way guarantee any specific benefit in outcome or survival. This paper was approved by the ASPEN Board of Directors.

*Target Population*: The target population is adult patients ≥16 years with head and neck cancer receiving any treatment modality (eg, chemotherapy, radiation, surgery, or a combination of therapies).

*Target Audience*: This clinical guideline is intended for use by any medical health professional involved in the nutrition care of adult patients with head and neck cancer (eg, dietitians, nurses, pharmacists, physicians, and speech‐language pathologists).

## METHODS

### The panels

The guideline was comprised of four panels: a clinical panel, a bias panel, a guidelines relief panel, and an external validation panel. The clinical panel was chaired by Nicole Kiss, PhD, APD. The clinical panel included dietitians, an epidemiologist/methodologist, a pharmacist, a radiation oncologist, and a speech pathologist. A nurse and an ear, nose, and throat physician were included in the early phases of the project, and although they were not included in the later activities of the clinical panel, they participated in the external validation panel to generate expert consensus and as independent journal reviewers before publication.

**Figure 1:**
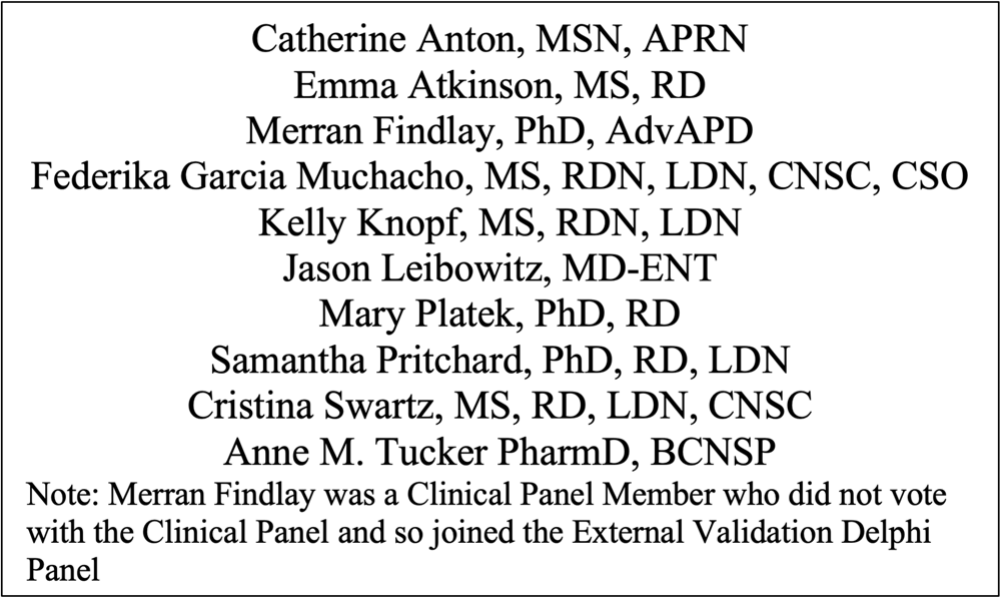
The external validation Delphi panel.

The role of the clinical panel was to create the guideline key questions using the Population, Intervention, Comparison, Outcome, and Timeframe (PICOT) framework, assist in the creation of search terms, perform the screening and data extraction, create the initial recommendations, and edit the final manuscript. The bias panel was composed of doctoral‐level researchers with a background in nutrition. They were trained and overseen by the epidemiologist/methodologist to perform bias analysis of the included studies. The guidelines relief panel was composed of an interdisciplinary team of dietitians, nurses, pharmacists, and physicians, and their role was to assist in screening and data extraction. Finally, the external validation panel was an interdisciplinary group of dietitians, a nurse, pharmacists, and an ear, nose, and throat physician (Figure 1). Their role was to review the recommendations approved by the clinical panel and provide external validation to the wording of the recommendations via a blinded vote.

### The protocol

An “a priori” protocol for this guideline was published and made available for public comment for 6 weeks.^6^ Emails were sent to members of ASPEN to solicit feedback from clinicians and researchers. Two patients with head and neck cancer were also solicited for feedback to ensure all key stakeholders had an equal opportunity to provide input. The protocol was adjusted based on the feedback received.

### Study inclusion/exclusion criteria

To be included, an article had to be a study of patients aged ≥16 years with head and neck cancer, published after January 1, 2001, whose primary or secondary objective was directly relevant to at least one of our questions and outcomes of interest. To include European studies that use a younger cutoff to define adults, the age of 16 years rather than 18 years was chosen to define an adult. For each question, we restricted to the study design most able to answer that specific question. The decision‐making process for this is outlined in the published protocol for this guideline.^6^ The result was that studies for all guideline questions were restricted to RCTs and quasi‐experimental designs. Study designs that were vulnerable to known unmanaged confounders were excluded. When such confounding is present, the resulting statistic is altered in magnitude and/or direction, losing its ability to meaningfully reflect the relationship under study.

### The search strategy

**Figure 2:**
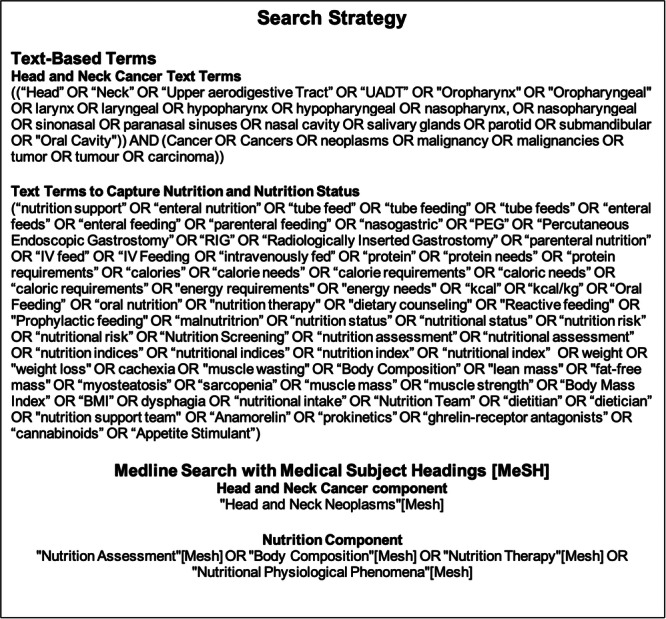
The search strategy.

A comprehensive search was performed in the PubMed database from 2001 to July 27, 2025. The decision to restrict the search to studies published after 2001 reflects modern radiation techniques, treatment modalities, and protocols. The PubMed search strategy may be viewed in Figure 2. Analogous search strategies were composed and run for the EMBASE, CINAHL, and Cochrane Central databases. The results of these searches were uploaded into Covidence for data collection.

### Data collection and presentation

All data were screened and extracted in duplicate in Covidence by researchers blinded to each other's decisions, except for the catch‐up search, which collects citations at the end of the guidelines process before journal submission. This was screened in duplicate but extracted directly by the guideline methodologist. Practice tests were performed before data collection to improve internal agreement.^6^ Any discrepancies among panel members’ study selections were adjudicated by a third party. Data were presented as narrative tables to account for the considerable heterogeneity in study design, outcome types, and timepoints explored. Forest plots were created wherever three or more studies were highly similar in population, intervention, comparator, outcome, and time point of measurement.

### Bias analysis

Bias of included studies was assessed in duplicate by the bias panel and checked by the methodologist. Each study received two bias assessments to account for differences in blinding bias between outcomes. The Cochrane Risk of Bias 2 tool^7^ was used to assess RCTs and the Cochrane Risk of Bias in Non‐Randomized Interventions tool^8^ was used to assess quasi‐experimental designs. Bias assessments are available in the Supplementary Materials.

### Statistical analysis

Wherever three or more studies were able to be meaningfully conflated, a summary statistic was run using a random‐effects model with a Knapp Hartung adjustment. Small study numbers (<10) tend to destabilize a summary statistic leading to statistical false positive results. The Knapp Hartung adjustment adjusts the error to account for this issue.^9^, ^10^ Publication bias was assessed through funnel plots and Egger tests wherever ≥10 studies are available for conflation into a forest plot.

### Recommendations

**Figure 3:**
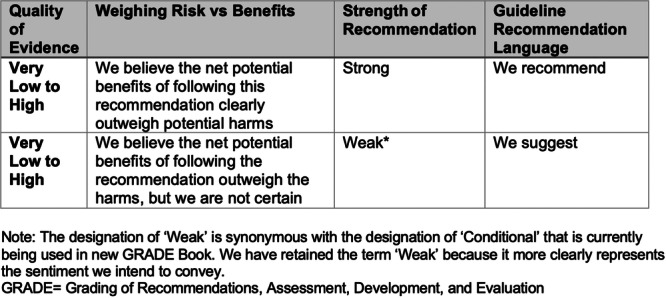
GRADE language for guideline recommendations. The designation of “weak” is synonymous with the designation of “conditional” that is currently being used in the new GRADE Book. We have retained the term “weak” because it more clearly represents the sentiment we intend to convey. GRADE, Grading of Recommendations, Assessment, Development, and Evaluation.

The Grading of Recommendations, Assessment, Development, and Evaluations (GRADE) tool was used to facilitate transparency in the creation of the guideline recommendations. The clinical panel assembled multiple times to discuss the recommendations for every key question and wrote the recommendations together. The GRADE process generates two major outputs: it generates a certainty level that the current evidence can answer the key questions, which ranges from “very low” to “high,” and it also generates a strength of the recommendation. For this guideline, where certainty existed that the potential benefits of following the recommendation outweighed any potential harms, the strength was listed as “strong.” Where we were less certain but still felt that the benefits outweighed the harms, the strength is listed as “weak” (Figure 3). New terminologies have recently been introduced by the GRADE group that have replaced the term “weak” with the term “conditional.” We have chosen not to adopt that alteration, but the meaning of the two terms may be considered equivalent for this guideline. The separation of certainty of evidence from recommendation strength makes it is possible to have a strong recommendation despite very low certainty of evidence if the clinical panel is certain that the potential benefits outweigh the potential harms.

A modified Delphi approach to generate expert consensus was performed, permitting a blinded vote by the clinical panel members. This continued until ≥70% agreement was met for each recommendation. Then, the recommendations were sent to the external validation panel, which repeated the modified Delphi. This was a larger panel, and so an 80% agreement was required. Whenever the requisite percent agreement was not met, the chair modified the recommendation, and the vote was rerun for that recommendation until sufficient agreement was reached.

## RESULTS

**Figure 4:**
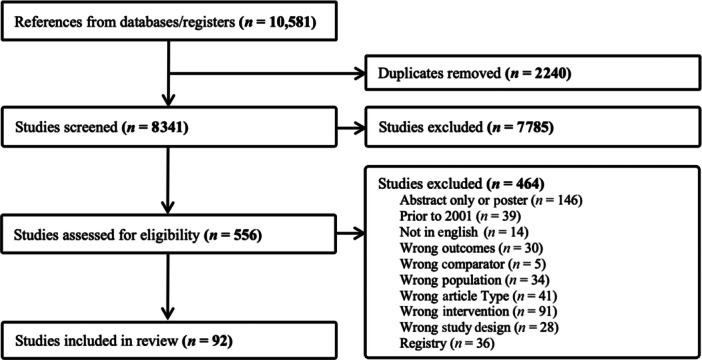
Inclusion flow diagram.

The search strategies yielded 10,581 citations. Of these, 2240 were duplicates and were removed. After review, 8249 were removed for not meeting the inclusion criteria. This left 92 studies for data extraction and inclusion in this guideline (Figure 4).

### Question 1a

In adult patients with head and neck cancer receiving chemoradiation or radiation, does earlier enteral nutrition (EN) vs later EN change progression‐free survival, overall survival, nutrition intake, nutrition status, weight, muscle mass, sarcopenia, myosteatosis, global quality of life, fatigue, return to work, performance status, treatment completion, treatment interruptions, treatment toxicities, or unplanned hospital admission?

*Recommendation*: In adults with head and neck cancer planned for or receiving radiotherapy with or without chemotherapy or other systemic therapy, we suggest initiation of enteral feeding when there is clinical evidence that nutrition intake or status is compromised, despite other strategies (oral nutrition supplements or food fortification) having been attempted. This should be individualized to oral nutrition intake, symptom burden, and nutrition status while considering individual treatment plans and clinical, psychosocial, and socioeconomic status.

**Certainty of Evidence:** Moderate

**Strength:** Strong

**Clinical Panel Delphi Agreement:** 100%

**External Validation Panel Delphi Agreement:** 100%

**Figure 5:**
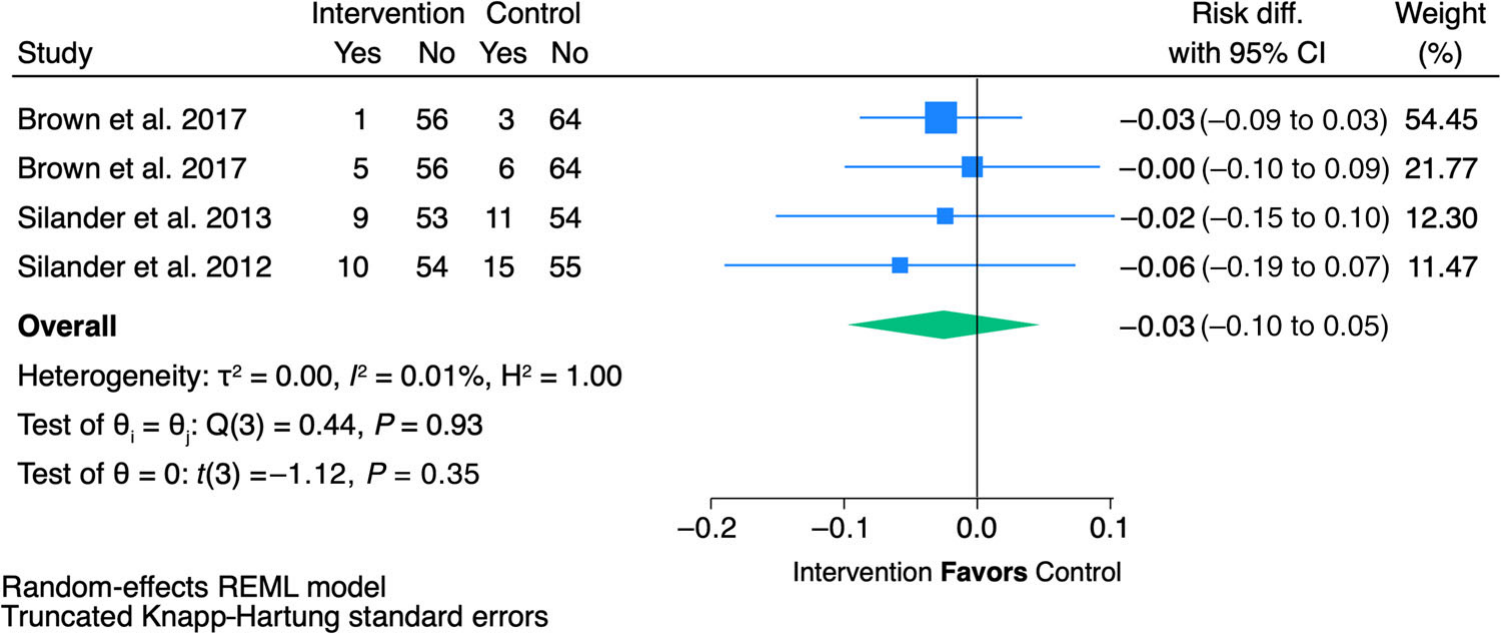
Forest plot for question 1a—1‐year mortality. diff., difference; REML, restricted maximum likelihood.

Six studies met the inclusion criteria for this PICOT question.^11^, ^12^, ^13^, ^14^, ^15^, ^16^ Four were RCTs and two were secondary analyses of RCTs examining different outcomes. Three studies demonstrated a significant benefit in the intervention vs the control. One study reported improved dysphagia at 1 year,^15^ another reported improved quality of life,^13^ and a third reported decreased length of hospital stay.^16^ The remainder found no significant differences between the two groups for any outcome (Tables 2 and 3). No studies favored the control. On average, patients in the intervention group had a 3% reduction in 1‐year mortality compared with the control, but this was not statistically significant (Risk Difference (RD) = −0.03, 95% CI = −0.1 to 0.05; *P* = 0.35) (Figure 5). Brown et al^11^ and its secondary analysis were found to be low risk of bias, whereas the other five studies were rated as having some concerns for bias (Tables S1 and S2).

**Table 2:** Summary of question 1a studies.

Study	Sample size	Population	Intervention	Comparator	Risk of bias
Brown et al^11^	124	Adult patients with HNSCC receiving radiation referred for prophylactic G‐tube	Supplemental prophylactic EN immediately following prophylactic G‐tube placement (bolus feeds [2 × 200 ml] with 1.5‐kcal/ml polymeric formula with fiber) per day, increased as needed	Standard care (EN following standard dietitian assessment)	Some concerns
Brown et al^12^	131	Adult patients with HNSCC referred for a prophylactic gastrostomy prior to treatment	Supplemental prophylactic EN immediately following prophylactic G‐tube placement (bolus feeds [2 × 200 ml] with 1.5‐kcal/ml polymeric formula with fiber) per day, increased as needed	Standard care	Low
Salas et al^13^	35	Adult patients with HNSCC (stages III and IV according to UICC 1997)	Percutaneous G‐tube systematically used prior to therapy	G‐tube only used when indicated	Some concerns
Silander et al^15^	134	Newly diagnosed, untreated, pharyngeal or oral cancer or malignant neck nodes with unknown primary in stage III or IV treated with curative intent. Intravenously treated with curative intent	Prophylactic PEG for early enteral feeding and nutrition advice when needed	Standard care	Some concerns
Silander et al^14^	65	Patients newly diagnosed with oral or pharyngeal cancer or neck lymph node metastases with unknown primary in stage III or IV	Prophylactic PEG for early enteral feeding and nutrition advice when needed	Standard care	Some concerns
Zeng et al^16^	80	Patients with oral and maxillofacial tumor	NGT placed 6 h before surgery	G‐tube placed POD 1	Some concerns

Abbreviations: EN, enteral nutrition; G‐tube, gastrostomy tube; HNSCC, head and neck squamous cell carcinoma; NGT, nasogastric tube; PEG, percutaneous endoscopic gastrostomy; POD, postoperative day; UICC, Union for International Cancer Control.

**Table 3:** Outcomes data for question 1a studies.

Topic	Outcome	Study	Timing of data collection	Results
Body composition/weight	% Weight change	Brown et al^12^	12 months	No difference between groups (*P* = 0.62)
	BMI change	Salas et al^13^	Baseline, during treatment (first day of the fourth week of radiotherapy), at the end of the treatment period, and 6 months after inclusion	Change in BMI was not significantly different between groups at any time point
	Weight loss	Silander et al^15^	6 months	By 6 months after treatment, the study group had lost 8.8 kg (11.2%), and the control group had lost 9.6 kg (12.4%; *P* = 0.08). When only patients who lost weight were included, the difference became significant (*P* < 0.05). At year 1 and year 2, the overall weight loss was not significantly different between groups (*P* = 0.52 and 0.33, respectively)
	Weight loss	Silander et al^14^	Baseline and 1, 2, 3, 6, 12, and 24 months	Weight loss was not significantly different between groups at any time point (*P* > 0.05)
	Weight loss	Zeng et al^16^	Baseline, discharge	Weight loss was not significantly different between groups (*P* = 0.145)
	Fat‐free mass % change	Brown et al^12^	12 months	Adjusted model, no difference (*P* = 0.19)
	Fat mass % change	Brown et al^12^	12 months	Adjusted model, no difference (*P* = 0.52)
	PG‐SGA risk score change	Brown et al^12^	12 months	Adjusted model, no difference (*P* = 0.74)
	SGA nutrition status % decline	Brown et al^12^	12 months	Adjusted model, no difference (*P* = 0.57)
	Malnutrition	Silander et al^15^	1, 2, 3, 6, 12, and 24 months	Percentage of malnutrition was directionally higher in the control group at most time points (2, 3, 4, and 12 months) but insignificant. The largest difference between groups occurred in month 2 (*P* = 0.06)
Length of stay	Hospital length of stay	Zeng et al^16^	N/A	Length of stay was 5.85 days shorter in the intervention vs the control group (*P* = 0.001)
Complications	Gastrostomy outcomes and complication	Brown et al^12^	12 months	No differences between groups
	Unplanned admissions	Brown et al^12^	12 months	No differences between groups
Dysphagia/swallowing issues	Dysphagia scale	Silander et al^15^	2 months, 6 months, 1 year	Dysphagia rose in both groups to 28%–29% after month 3. After 1 year, one patient still had the problem in the intervention compared with nine in the control group (*P* = 0.047). At 1‐year follow‐up, 20% in the control compared with 7% in the study group still had dysphagia
	Percentage of patients reporting problems swallowing solid food (EORTC QLQ‐H&N35 swallowing scale)	Silander et al^14^	Baseline and 1, 2, 3, 6, 12, and 24 months	No statistically significant differences were reported, and no consistent pattern of difference between the two groups is visible from the charts presented
Energy intake/EN	Energy intake	Silander et al^14^	Baseline, 1, 2, 3, 6, 12, and 24 months	Energy intake was not significantly different between groups at any time points
QOL	QOL: EORTC QLQ‐C30 and QLQ‐H&N35	Silander et al^15^	Baseline; 2, 3, and 6 months; and 1 and 2 years	Baseline and 1‐year follow‐up: No significant differences were observed between the groups at the start of the study. Although QOL deteriorated during treatment in both groups, most functions returned to baseline by the 1‐year follow‐up, except for persistent issues related to treatment side effects (eg, dry mouth, sticky saliva, mouth opening, and taste/sensory problems) 2‐month follow‐up: The study group showed better physical functioning (75 vs 66 points, *P* < 0.05) and less constipation, whereas the control group had better sensory function and less local pain 3‐month follow‐up: The study group significantly outperformed the control group in role and social functioning, appetite loss, constipation, and diarrhea. The control group had fewer problems with dry mouth (*P* < 0.05) 6‐month follow‐up: The most notable differences were observed, with 10 significant improvements in the study group, including global QOL; physical, cognitive, and role functioning; and reduced fatigue, dyspnea, feeling ill, coughing, mouth opening, and sexuality (*P* < 0.05) 1‐year follow‐up: The study group showed improvements in social functioning and dyspnea (*P* < 0.05) 2‐year follow‐up: The study group continued to show clinically significant improvements in mouth opening and sticky saliva (*P* < 0.05)
	GHS EORTC	Salas et al^13^	Baseline, during treatment (first day of the fourth week of radiotherapy), at the end of the treatment period, and 6 months after inclusion	No significant difference in GHS was noted at any time point
	EORTC QLQ‐C30	Brown et al^12^	12 months	No differences were seen for any domain
	PCS‐SF36	Salas et al^13^	Baseline, during treatment (first day of the fourth week of radiotherapy), at the end of the treatment period, and 6 months after inclusion	Patients with systematic gastrostomy experienced a greater decline in physical QOL at the start of radiotherapy compared with those without the procedure (*P* = 0.04). The rest of the time points were not significant
	MCS‐SF36	Salas et al^13^	Baseline, during treatment (first day of the fourth week of radiotherapy), at the end of the treatment period, and 6 months after inclusion	At the end of treatment, patients who underwent systematic gastrostomy had better mental QOL improvement from baseline compared with those who did not (*P* = 0.04). No significant difference was noted for any other time point
Survival/mortality	Mortality	Brown et al^11^	People died at month 3, 5, and 12	There were no significant differences, although more died in the control group. Two vs zero at month 3 (*P* = 0.21), threevs 0 by month 5 (*P* = 0.09), and three vs one by month 12 (*P* = 0.33). Statistic used was Fisher exact test
	Mortality	Brown et al^12^	12 months	No differences between groups. Six deaths in standard‐care group and five in the intervention group
	Mortality	Silander et al^14^	12 months, 24 months	No difference in mortality at 12 months (*P* = 0.81) or at 24 months (*P* = 0.41). Statistic used was Fisher exact test
	6‐month mortality	Salas et al^13^	6 months	No significant difference in mortality between intervention (10%) vs control (11%) (*P* = 0.64)
	1‐ and 2‐year survival	Silander et al^15^	1 year, 2 years	The 1‐year survival rate is 84% in the study group and 79% in the control group, with no significant difference between them (*P* = 0.52). The 2‐year survival rate is 77% in the study group and 69% in the control group, with no significant difference between them (*P* = 0.40)
	60‐month survival	Silander et al^15^	60 months	Kaplan‐Meier curve showed no difference in survival over 60 months (*P* = 0.41)
	Disease‐free survival	Brown et al^12^	12 months	Nineteen cases of disease relapse in the standard‐care group and 10 cases in the intervention group (*P* = 0.135)
Treatment tolerance/completion	Radiotherapy tolerance	Brown et al^12^	12 months	No differences between groups; 100% completion in standard care vs 95% in the intervention (*P* = 0.102)
	Chemotherapy tolerance	Brown et al^12^	12 months	No differences between groups; 59% completion in standard care vs 51% in intervention (*P* = 0.361)
	Treatment interruption	Silander et al^15^	Baseline, 12 months, 24 months	There was one treatment interruption in the study group vs seven in the control (*P* = 0.08)
Tube removal	Median days to tube removal after treatment	Brown et al^11^	1 year, baseline, 12 months	No meaningful differences were found in rate of tube removal between groups. However, a post hoc analysis found that among disease‐free patients, the prophylactic G‐tube group had higher tube usage at 4 months and slower removal rates
	Mean day of tube removal after treatment	Brown et al^12^	12 months	No differences between groups, (*P* = 0.33)

Abbreviations: BMI, body mass index; EN, enteral nutrition; EORTC, European Organisation for Research and Treatment of Cancer; GHS, Global Health Status; G‐tube, gastrostomy tube; MCS‐SF36, Mental Component Summary of the 36‐Item Short Form Survey; N/A, not applicable; PCS‐SF36, Physical Component Summary of the 36‐Item Short Form Survey; PG‐SGA, Patient‐Generated Subjective Global Assessment; QLQ‐C30, Quality of Life Questionnaire–Core 30; QLQ‐H&N35, Quality of Life Questionnaire–Head and Neck 35; QOL, quality of life; SGA, Subjective Global Assessment.

### Rationale and discussion

Patients with head and neck cancer have a high prevalence of malnutrition and muscle loss, which has been associated with worse outcomes.^17^, ^18^, ^19^ Proactive nutrition intervention may minimize or prevent the decline in nutrition status for these patients.^20^ Early commencement of EN may, therefore, be an appropriate strategy to meet energy and protein requirements while preventing nutrition decline. The current recommendation is based on this potential benefit, along with some evidence of benefit from the literature, and low risk of harm and aligns with a similar recommendation in the COSA guideline.^5^

The timing and amount of nutrition received varied considerably between the studies. Two studies placed their percutaneous endoscopic gastrostomy (PEG) tubes prophylactically in both groups and commenced feeding the intervention group immediately, whereas the control group did not commence until weight loss or reduced intake were noted. The third study restricted PEG placement to the intervention group. Feeding in the intervention group commenced only if inadequate intake was noted, whereas the control group was left to standard care. It is important to note that, in all studies, adherence to EN was poor and patients were unable to achieve their recommended energy intake. So, aiming to commence early EN does not necessarily equate to achieving energy goals, and the one study that tracked energy intake reported no differences between the groups.^14^

### Future research

The true effect of achieving the interventions in the studies for this PICOT question is unknown because of poor adherence to the interventions and variability in implementation. Randomized trials of interventions using structured protocols to achieve energy and protein requirements are needed, with the ability to tailor the intervention to individual patients. These protocols should use strategies that are common in clinical practice (eg, food fortification or modification initially) and should describe criteria for when oral nutrition supplements or EN should be initiated. They should assess the effect on nutrition status, treatment completion/interruptions, treatment toxicities, unplanned hospital admissions, and survival. Similar interventions in nonrandomized studies have shown benefit for some of these outcomes.^21^, ^22^ Codesigning the protocol with patients and other stakeholders is suggested to ensure patient and healthcare provider perspectives and the barriers to early EN are considered. This is consistent with suggestions in a recently published roadmap for nutrition research on head and neck cancer care.^23^

#### Question 1b

In adult patients with head and neck cancer, does longer postoperative nutrition (EN or oral nutrition supplements) vs shorter duration of nutrition support change progression‐free survival, overall survival, nutrition intake, time to transition to full oral diet, nutrition status, weight, muscle mass, sarcopenia, myosteatosis, global quality of life, fatigue, return to work, performance status, length of stay, surgical complications, or hospital readmissions?

*Recommendation*: In adults with head and neck cancer, we suggest early commencement (within 24 h) of postoperative nutrition intake (EN, oral, or otherwise) to meet estimated nutrition requirements. Early initiation of oral intake should occur in consultation with the surgical team, dietitian, and speech pathologist. To meet nutrition requirements, oral intake should be increased gradually, and supplemental enteral feeding should be maintained until sufficient oral intake is established. Early initiation of oral intake may be inappropriate in certain situations, making consultation with surgical, speech, and nutrition teams critical.

**Certainty of Evidence:** Very low

**Strength:** Weak

**Clinical Panel Delphi Agreement:** 100%

**External Validation Panel Delphi Agreement:** 100%

Two studies met the inclusion criteria for this question.^24^, ^25^ No studies found significant differences between the intervention and control for any outcome (Tables 4 and 5). One study reported a longer length of hospital stay in the control group, but did not provide statistics.^24^ No studies favored the control. One study was found to have “some concerns” for bias, and the other, a quasi‐experimental design, was found to be a “serious” risk for bias (Tables S3–S5).

**Table 4:** Summary of question 1b studies.

Study	Sample size	Population	Intervention	Comparator	Risk of bias
Prasad et al^24^	78 Quasi‐experimental	Patients who underwent laryngectomy	Oral feeds initiated at POD 2	Oral feeds initiated at POD 10	Serious risk
Sharifian et al^25^	25	Patients who underwent total laryngectomy for a malignant tumor of the larynx	Feeds initiated orally with sips of water on POD 3	NGT placed and oral feeds not initiated until POD 7	Some concerns

Abbreviations: NGT, nasogastric tube; POD, postoperative day.

**Table 5:** Outcomes data for question 1b studies.

Topic	Outcome	Study	Timing of data collection	Results
Complications	Aspiration	Prasad et al^24^	Patients followed until they left the hospital	Aspiration was 5.26% in the intervention vs 2.5% in the control (*P* = 0.61)
	Flap necrosis	Prasad et al^24^	Patients followed until they left the hospital	Flap necrosis was not different between the groups (*P* = 0.61)
	Wound infection	Prasad et al^24^	Patients followed until they left the hospital	Wound infection was not different between groups (*P* = 1.0)
	Pharyngeocutaneous fistulas	Sharifian et al^25^	Weekly follow‐up for first month, then monthly for 4 months	No difference clinically or statistically between groups
	Pharyngeocutaneous fistulas	Prasad et al^24^	Patients followed until they left the hospital	Two patients in control group (0.76%) and one in the study group (0.8) had a pharyngeocutaneous fistula. Statistics were not presented, but *P* = 0.61 by Fisher exact test
Length of stay	Hospital length of stay	Prasad et al^24^	Patients followed until they left the hospital	Authors do not provide statistics or enough data to analyze, but they state that hospital length of stay was longer in the control group

### Rationale and discussion

Patients with head and neck cancer are at risk of malnutrition and muscle loss preoperatively and postoperatively.^26^ Malnutrition and low muscle mass are associated with adverse postoperative outcomes, such as surgical complications, delayed wound healing, longer hospital stay, and poorer survival.^19^, ^26^ The timing of initiating nutrition support, including oral nutrition support, may be important in preventing decline in nutrition status and, therefore, adverse postoperative outcomes. The current recommendation is based on this potential benefit, along with no evidence of harm from early oral nutrition in the two included studies, and aligns with similar recommendations in the COSA guideline and other guidelines.^5^, ^27^

Whether or not commencing oral intake is appropriate depends on factors such as the extent of surgical resection and/or reconstruction, severity of dysphagia, risk of aspiration, and other symptoms. Some patients will require long‐term EN and may be unable to tolerate oral intake. Therefore, the timing of the introduction of oral intake and the duration of EN should be determined collaboratively through consultation among the surgical team, speech and language pathologist, and dietitian.

### Future research

No studies specifically addressed the optimal duration of postoperative EN or oral nutrition supplements. In clinical practice, these therapies may stop before postoperative discharge regardless of the patient's nutrition intake and/or nutrition status. Randomized trials are needed to compare continuation of EN or oral nutrition supplements until oral intake is adequate to meet energy and protein requirements to standard care and assess the effect on nutrition status, time to transition to full oral diet, surgical complications, postoperative length of stay, hospital readmissions, and survival. Such studies should stratify participants by the site and extent of surgical resection and/or reconstruction.

#### Question 1c

In adult patients with head and neck cancer, does increasing the frequency of dietetic intervention vs standard care during chemoradiation or radiation and up to 3 months posttreatment change progression‐free survival, overall survival, nutrition intake, time to transition to full oral diet, nutrition status, weight, muscle mass, sarcopenia, myosteatosis, global quality of life, fatigue, return to work, performance status, treatment completion, treatment interruptions, treatment toxicities, or unplanned hospital admissions?

*Recommendation*: In adults with head and neck cancer receiving radiotherapy with or without chemotherapy or other systemic therapy, we recommend weekly consultation with a dietitian during treatment and every 2 weeks for up to 6 weeks after treatment to maintain nutrition status and quality of life while preventing unplanned hospital admissions and early cessation of treatment.

**Certainty of Evidence:** High

**Strength:** Strong

**Clinical Panel Delphi Agreement:** 100%

**External Validation Panel Delphi Agreement:** 100%

Thirteen studies met the inclusion criteria for this question.^28^, ^29^, ^30^, ^31^, ^32^, ^33^, ^34^, ^35^, ^36^, ^37^, ^38^, ^39^, ^40^ Nine studies demonstrated a significant benefit in the intervention compared with the control.^28^, ^29^, ^33^, ^34^, ^36^, ^37^, ^38^, ^39^, ^40^ Four studies reported lower rates of malnutrition,^28^, ^29^, ^34^, ^36^ seven found less weight loss,^28^, ^33^, ^36^, ^37^, ^38^, ^39^, ^40^ two found fewer treatment interruptions,^28^, ^33^ five found improved quality of life,^28^, ^34^, ^37^, ^38^, ^39^ four reported higher energy intake,^29^, ^34^, ^38^, ^39^ two reported higher protein intake,^34^, ^38^ one reported reduced incidence of symptoms at 3‐months,^34^ and one reported fewer hospital admissions due to mucositis.^33^ The remainder found no significant differences between the two groups. No studies favored the control. Two studies were rated as having “low” risk of bias. Seven had “some concerns” for bias. One RCT was rated at “high” risk for bias, and three quasi‐experimental designs were rated at “serious” risk for bias (Tables 6, 7, and S6–S9).

**Table 6:** Summary of question 1c studies.

Study	Sample size	Population	Intervention	Comparator	Risk of bias
Britton et al^28^	307	Adult patients with HNC requiring definitive or postoperative radiation therapy with curative intent	Motivational interviewing and cognitive behavioral therapy delivered by RDs	Standard care	Low
Chen et al^37^	108	Adult patients with HNC	Individualized nutrition intervention tailored to patient needs during RT	Standard nutrition care	Some concerns
Dai et al^38^	72	Adult patients with HNC receiving radical CCRT	Individualized counseling with a dietitian counseling every 2 weeks during CCRT with algorithm for ONS or EN where needed	Standard care	Low
Kang et al^29^	40	Patients with HNC who underwent RT in 323 Hospital of Chinese People's Liberation Army (Xi'an, China)	Intensive dietary counseling before and after radiation therapy but little detail provided as to what that entailed	Standard care	Some concerns
Leistra et al^30^	190 Quasi‐experimental	Patients with HNC treated with either RT, chemoradiotherapy, or surgery	Patients were consulted by a dietitian within 1 week after their first outpatient visit (in pretreatment period)	Standard care in which dietetic consultation begins at start of antitumor treatment	Serious
Li et al^39^	103 Quasi‐experimental	Adult patients with HNC after RT	Personalized nutrition plans and education with the help of dietitians	Standard care	Serious
Löser et al,^31^	61 As‐treated design (ie, not ITT)	Adult patients with squamous cell carcinoma of the oropharynx, oral cavity, hypopharynx, larynx, and salivary glands, without distant metastases referred for CRT or RT with curative intent	The intervention (arm A) consisted of individualized nutrition counseling (by RD) based on the results of the diet diary, BIA, blood cell counts, and the patient's clinical condition (eg, presence of gastric feeding tube); reassessed every 2 weeks	No nutrition counseling	Some concerns
Orell et al^32^	58	Patients with locally advanced (stage III or IV) squamous cell carcinoma of the oral cavity, oropharynx, hypopharynx, nasopharynx, or larynx, referred for a curative treatment	Intensive nutrition counseling	On‐demand counseling	Some concerns
Paccagnella et al^33^ Quasi‐experimental	66	Adult patients with HNC receiving CRT	Intervention received nutrition assessment before the beginning of the RT. Ambulatory visits were initially every 7 days throughout the CRT period, followed by 14–28 days of control visits	Standard care before 2006	Serious
Ravasco et al^34^	75	Ambulatory adult patients with HNC	Patients who received dietary counseling with regular food (intervention 1) and ONS (intervention 2)	Patients who maintained intake ad libitum	Some concerns
Roussel et al^35^	87	Adults with HNC treated with RT	Intensive nutrition care (care based on protocols plus six meetings with RD)	Standard care based on European protocols	Some concerns
Sykes et al^40^	49	Adults with HNC undergoing free flap reconstruction	Weekly dietitian consultations for 3 weeks before surgery	Standard care with no dietitian consult	Some concerns
Van den Berg^36^	38	Adult patients with primary squamous cell carcinoma in the oral cavity, oropharynx, or hypopharynx stages II–IV	Individual dietary counseling with RD through treatment and after	Standard care	High

Abbreviations: BIA, bioelectrical impedance analysis; CCRT, concurrent chemoradiotherapy; CRT, chemoradiation therapy; EN, enteral nutrition; HNC, head and neck cancer; ITT, intention‐to‐treat; ONS, oral nutrition supplement; RD, registered dietitian; RT, radiotherapy.

**Table 7:** Outcomes data for question 1c studies.

Topic	Outcome	Study	Timing of data collection	Results
Complications, treatment toxicities, and unplanned events	Percentage of patients with grade 3–4 mucositis	Paccagnella et al^33^	Baseline: fourth week of RT; at completion of chemoradiation; and at 1, 3, and 6 months after the end of chemoradiation	The percentage of patients with grade 3–4 mucositis was higher in the intervention vs the control group but this was NS (45.5 vs 39.4%)
	Percentage of patients admitted to hospital for mucositis	Paccagnella et al^33^	Baseline: fourth week of RT; at completion of chemoradiation; and at 1, 3, and 6 months after the end of chemoradiation	A lower percentage of patients were admitted to the hospital with mucositis in the intervention vs the control group (16.1 vs 41.4%, *P* = 0.03)
	Overall major complications	Leistra et al^30^	First outpatient visit, start of primary treatment, and end of primary treatment	There were no notable differences in major complications (*P* = 0.49), such as reoperation, readmission, ICU admission, or hospital mortality
	Symptom‐induced morbidity	Ravasco et al^34^	Baseline to end of radiation treatment, and 3 months	By 3 months, the decrease in both the incidence and severity of grade 1 and 2 symptoms (anorexia, nausea/vomiting, xerostomia, and dysgeusia) varied between the groups. Improvement was observed in 90% of patients in group 1, compared with 67% in group 2 and 51% in group 3 (*P* < 0.0001), with group 1 showing greater improvement than groups 2 and 3 (*P* < 0.07). The reduction in the incidence and severity of grade 1 and 2 dysphagia/odynophagia did not differ significantly between the groups (*P* < 0.09)
	Unplanned admissions	Britton et al^28^	First week RT, last week RT, 1 month post‐RT, 3 months post‐RT	There were more unplanned admissions in the control vs intervention group (130 vs 100, respectively), but this did not achieve significance (*P* = 0.07)
	Complications Wounds, infections, surgical complications, 30‐day readmission	Sykes et al^40^	Not applicable	No difference was found between groups for wound breakdown, infections requiring antibiotics, surgical complications, or 30‐day readmissions
Length of stay	Hospital length of stay	Britton et al^28^	Not applicable	Mean length of stay was less in the intervention group vs the control but did not achieve significance (β = −1.80, *P* = 0.13).
	Hospital length of stay	Leistra et al^30^	Not applicable	Length of stay was similar between intervention and control (18.5 vs 19.3 days, *P* = 0.79)
	Hospital length of stay	Sykes et al^40^	Not applicable	Length of stay was similar between intervention and control (8.0 vs 7.0 days, *P* = 0.20)
Malnutrition	SGA	Britton et al^28^	First week RT, last week RT, 1 month post‐RT, 3 months post‐RT	Intervention group was more likely to be labeled SGA category A (well‐nourished) across all time points (*P* < 0.01) and in the final week (*P* = 0.03)
	SGA	Kang et al^29^	Unclear	The intervention group had fewer patients categorized at severely malnourished compared with the control (*P* < 0.05)
	Patient‐Generated SGA	Orell et al^32^	Baseline and end of treatment	No significant differences between groups
	Patient‐Generated SGA	Ravasco et al^34^	Baseline to end of radiation treatment, and 3 months	More patients improved their nutrition status in the intervention group compared with intervention 2 and the control (*P* < 0.001) Fewer patients in the intervention group declined in nutrition status compared with intervention 2 and the control (*P* = 0.001)
	Nutrition risk	Löser et al,^31^	End of therapy	No differences were found in nutrition risk between groups using any of the following tools/methods: MUST, NRS‐2002, Nutri‐Score, BMI <18, low fat‐free mass index (however, nothing was done to adjust for baseline differences, which were considerable)
	Malnutrition	Van den Berg^36^	Before, during, 2 weeks after, and 2 months after treatment	Malnutrition was significantly less in the intervention group vs the control group by week 2 (*P* = 0.02). Malnutrition continued to increase over the time points in the control group, but no statistical analyses were performed to determine significance to corroborate
Nutrition intake	Energy intake	Kang et al^29^	Unclear	The intervention group received more energy vs the control (1691 vs 1066 kcal, *P* < 0.05)
	Energy intake	Roussel et al^35^	1 and 3 months post‐RT	No difference in energy intake at either time point (1 month, *P* = 0.41; 3 months, *P* = 0.07)
	Mean energy (kcal) increase per day	Ravasco et al^34^	Baseline to end of radiation treatment, and 3 months	By end of RT, intervention 1 increased by 521 kcal/day (*P* = 0.002). Intervention 2 increased by 322 kcal/day (*P* = 0.05). The control decreased by 400 kcal/day (*P* < 0.01). Intervention 1 is the only group that maintained its change at month 3. The others decreased back toward baseline
	Energy intake	Li et al^39^	Start of RT to end of RT	The intervention received 522.28 kcal more than the control group (*P* < 0.001)
	Energy intake	Dai et al^38^	Baseline and 2, 4, and 6 weeks	Energy intake was significantly high in the intervention vs the control starting by week 4–6 (*P* < 0.001)
	Change in energy deficit	Löser et al,^31^	End of therapy	By the end of treatment, the intervention group had accumulated a lower energy deficit compared with the control, but analysis of significance was not performed or possible based on the data provided
	Percentage of patients reaching >90% of estimated energy needs	Orell et al^32^	Baseline and end of treatment	More people in the intervention group reached >90% of estimated energy needs compared with the control group (31% vs 19%, *P* = 0.06)
	Protein intake	Dai et al^38^	Baseline and 2, 4, and 6 weeks	Protein intake was significantly higher in the intervention vs the control at every time point (*P* < 0.001)
	Protein intake	Roussel et al^35^	1 and 3 months post‐RT	No difference in protein intake at either time point (1 month, *P* = 0.50; 3 months, *P* = 0.79)
	Mean protein intake increase per day	Ravasco et al^34^	Baseline to end of radiation treatment and 3 months	By end of RT, intervention 1 increased protein by 26 g/day (*P* = 0.006). Intervention 2 increased by 35 g/day (*P* = 0.001). Protein intake decreased in the control by 15 g/day (*P* = 0.01). Intervention 1 is the only group that maintained its change at month 3. The others decreased back toward baseline
	Percent reaching >90% of estimated protein needs	Orell et al^32^	Baseline and end of treatment	Fewer people in the intervention group reached >90% of estimated protein needs compared with the control group, but this was statistically insignificant (11.5% vs 12.5%, *P* = 0.24)
Survival	2‐year survival	Löser et al,^31^	2 years	Survival was given as percentages (79% in the intervention and 70% in the control), but this was not statistically significant (*P* = 0.79)
	5‐year overall survival	Orell et al^32^	Median follow‐up was 43 months (range, 6–63)	Overall survival was 69% (18/26) in the intervention group and 53% (17/32) in the control group (*P* = 0.81)
	Disease‐specific survival (ie, death was not from cancer)	Orell et al^32^	Median follow‐up was 43 months (range, 6–63)	Disease‐specific survival was 75% (18/24) in the intervention group and 68% (17/25) in the control group (*P* = 0.56)
	Disease‐free survival (ie, death was from cancer)	Orell et al^32^	Median follow‐up was 43 months (range, 6–63)	Disease‐free survival was 65% in the intervention group (17/26) and 41% (13/32) in the control group (*P* = 0.94)
Treatment adherence	Chemotherapy completion	Orell et al^32^	Baseline and end of treatment	61% in the intervention vs 60% in the control completed chemotherapy (*P* = 0.33)
	Percentage of patients completing chemotherapy	Paccagnella et al^33^	Baseline, fourth week of RT, at completion of chemoradiation, and at 1, 3, and 6 months after the end of chemoradiation	The intervention group had a higher percentage complete chemotherapy compared with the control, but this was NS (96.7% vs 93.9%)
	Radiation therapy interruptions	Britton et al^28^	First week RT, last week RT, 1 month post RT, 3 months post RT	Decreased treatment interruptions in the intervention group: 8% vs 14% (*P* = 0.04).
	Radiation completion	Orell et al^32^	Baseline and end of treatment	92% in the intervention vs 91% in the control completed radiation (*P* = 0.38)
	Days of radiation therapy delayed for toxicity	Paccagnella et al^33^	Baseline, fourth week of RT, at completion of chemoradiation, and at 1, 3, and 6 months after the end of chemoradiation	Patients in the intervention group had shorter delays to their radiation therapy compared with the control group (4.4 vs 7.6 days, *P* = 0.04)
	Patients who had RT breaks (>5 days) for toxicity	Paccagnella et al^33^	Baseline, fourth week of RT, at completion of chemoradiation, and at 1, 3, and 6 months after the end of chemoradiation	A lower percentage of patients in the intervention required radiation breaks >5 days compared with the control group (30.3 vs 63.6%, *P* = 0.007)
QOL	EORTC QLQ‐C30 version 3 EORTC QLQ‐H&N35 EQ‐5D‐3L EQ VAS	Roussel et al^35^	Baseline to 3 months post‐RT	No differences on any domain of either scale except that there was more coughing (*P* = 0.04) and deterioration in sleep (*P* = 0.04) in the control group at 3 months but improvement in the intervention group
	EORTC QLQ‐C30	Ravasco et al^34^	Baseline to end of radiation treatment and 3 months	Pairwise comparisons were not calculated. This precludes group comparison, but in general, by the end of RT, all QOL function scores improved significantly in intervention 1 and were maintained at month 3. The opposite was true for the control. Intervention 2 saw similar improvements in function scores, but these were only proportional to protein intake
	HRQOL score	Britton et al^28^	First week RT, last week RT, 1 month post‐RT, 3 months post‐RT	Overall QOL was better in the intervention group vs the control (β = 0.45, *P* < 0.01). Significant domains included physical, emotional, and cognitive functioning. The intervention group also has decreased nausea and vomiting (*P* < 0.01) and appetite loss (*P* = 0.02)
	HRQOL C30 score	Chen et al^37^	Baseline to 6 months	QOL was improved for every domain in the intervention vs the control (*P* < 0.05)
	Mean HRQOL C30 score	Li et al^39^	Start of RT to 3 months post‐RT	At 3 months post‐RT, the intervention group had better mean scores on HRQOL C30 compared with the control (*P* < 0.001)
	Anxiety, depression, and mental health: SAS, SDS, SCL‐90	Chen et al^37^	Baseline to 6 months	At the 6‐month mark, relative to the control, the intervention group had improved anxiety (*P* = 0.007), depression (*P* = 0.022), and broad mental health (*P* = 0.042)
	Anxiety and depression: HADS‐A and HADS‐D	Dai et al^38^	Baseline and 2, 4, and 6 weeks	The intervention group, relative to the control, had lower anxiety (*P* < 0.001) and depression (*P* = 0.01) at every time point
	Anxiety and depression scores: SAS, SDS	Li et al^39^	Start of RT to 3 months post‐RT	At 3 months post‐RT, the intervention group had better scores than the control on both anxiety (*P* = 0.011) and depression (*P* = 0.001)
Weight, body composition, strength	Weight loss	Roussel et al^35^	Baseline to 3 months post‐RT	The intervention group lost less weight over 3 months compared with the control, but this was NS (3.6 vs 4.4 kg, *P* = 0.59)
	Weight loss	Van den Berg^36^	2‐month weight loss	More weight was lost in the control group, which continued to lose weight, whereas the intervention group gained weight (*P* = 0.03)
	Weight loss	Leistra et al^30^	First outpatient visit, start of primary treatment, and end of primary treatment	No significant differences were found between groups. The intervention lost 0.7% body weight, whereas the control lost 0.2% body weight (*P* = 0.53)
	Weight loss	Paccagnella et al^33^	Baseline, fourth week of RT, at completion of chemoradiation, and at 1, 3, and 6 months after the end of chemoradiation	The control group lost significantly more weight over all time points (*P* = 0.02) and at every individual time point compared with patients in the intervention group. The intervention regained much of the weight lost over the time points, whereas the control group lost weight at every subsequent time point
	Weight loss	Dai et al^38^	Baseline 2,4, and 6 weeks	Weight loss is significantly more severe in the control vs the intervention group starting week 4–6 (*P* < 0.001)
	Weight change	Orell et al^32^	Baseline and end of treatment	No difference between groups (*P* = 0.69)
	Percent weight loss	Britton et al^28^	First week RT, last week RT, 1 month post‐RT, 3 months post‐RT	Intervention group less likely to lose weight compared with intervention (β = −1.24, *P* = 0.04)
	Median percent weight loss	Löser et al,^31^	Beginning of therapy to end of therapy	Weight loss difference between the two groups was highly insignificant (*P* = 0.82)
	Weight/BMI	Sykes et al^40^	Baseline, day of surgery	No changes were found for weight or BMI in the intervention group, but significant decreases across time points were found for both weight (*P* = 0.001) and BMI (*P* = 0.003) in the control
	Changes in BMI	Chen et al^37^	Baseline to 6 months	BMI was higher at 6 months in the intervention vs the control group (*P* = 0.03)
	Changes in BMI	Li et al^39^	Start of RT to end of RT	BMI was high at end of RT in the intervention vs the control group (*P* = 0.004)
	Changes in BMI	Löser et al,^31^	Beginning of therapy to end of therapy	Exact values are not given (only CIs), but this was not statistically significant (*P* = 0.46)
	BMI change	Orell et al^32^	Baseline and end of treatment	No difference between groups (*P* = 0.66)
	BMI loss	Roussel et al^35^	Baseline to 3 months post‐RT	The intervention group has less BMI deterioration over 3 months compared with the control, but this was NS (1.2 vs 1.5 kg, *P* = 0.69)
	BMI loss	Van den Berg^36^	2‐month weight loss	No difference in BMI loss over the 2‐month period; no statistics provided
	Fat mass change	Orell et al^32^	Baseline and end of treatment	Fat mass was 0.6 kg lower in the control group, but this was NS (*P* = 0.33)
	Fat‐free muscle index change	Orell et al^32^	Baseline and end of treatment	No difference between groups (*P* = 0.74)
	Fat‐free muscle change	Orell et al^32^	Baseline and end of treatment	No difference between groups (*P* = 0.68)
	Handgrip strength	Orell et al^32^	Baseline and end of treatment	No difference between groups (*P* = 0.80)

Abbreviations: BMI, body mass index; EORTC, European Organisation for Research and Treatment of Cancer; EQ‐5D‐3L, EuroQol 5‐Dimension 3‐Level questionnaire; EQ VAS, EuroQol Visual Analog Scale; HADS‐A, Hospital Anxiety and Depression Scale–Anxiety subscale; HADS‐D, Hospital Anxiety and Depression Scale–Depression subscale; HRQOL, health‐related quality of life; ICU, intensive care unit; MUST, Malnutrition Universal Screening Tool; NRS‐2002, Nutrition Risk Screening 2002; NS, not significant; QLQ‐C30, Quality of Life Questionnaire–Core 30; QLQ‐H&N35, Quality of Life Questionnaire–Head and Neck 35; QOL, quality of life; RT, radiotherapy; SAS, Self‐Rating Anxiety Scale; SCL‐90, Symptom Checklist‐90; SDS, Self‐Rating Depression Scale; SGA, Subjective Global Assessment.

### Rationale and discussion

Patients with head and neck cancer are at high risk of malnutrition and muscle loss during and following radiotherapy with or without chemotherapy.^17^ Observational studies show that malnutrition and low muscle mass are associated with unplanned hospital admission, poorer quality of life, and reduced survival.^17^, ^41^, ^42^ The frequency of dietitian intervention may be important to minimize this decline in nutrition status and prevent adverse outcomes. The current recommendation is based on consistent evidence of benefit from the literature and no evidence of harm other than the possible inconvenience of additional appointments and aligns with a similar recommendation in the COSA guideline.^5^

The frequency of dietitian interventions varied throughout the studies, with the most common schedule being weekly during treatment and every second week for 6 weeks following treatment. Cancer services should consider how they can provide this level of nutrition care while minimizing appointment fatigue for patients. Colocated interdisciplinary clinics, such as combined dietitian and speech‐language therapy clinics, may be beneficial to streamline care. Cancer services with limited dietetic resources may need to evaluate how best to provide this care to patients receiving radiotherapy with or without chemotherapy while also ensuring equitable access to dietetic care for patients undergoing surgery, which may require prioritizing patients at the highest risk of malnutrition.

### Future research

The current evidence is sufficient to conclude that increased frequency of dietetic interventions improves important clinical outcomes. Future research in this area should focus on how best to deliver this care equitably, efficiently, and sustainably in routine practice. This includes testing models of care, for example, telehealth‐enabled approaches, codesigned care pathways, workforce models that optimize specialist availability, and risk‐based triage tools to support prioritization and access. Implementation‐effectiveness studies should evaluate clinical outcomes such as treatment completion, unplanned hospitalization, nutrition status, quality of life, and long‐term survival alongside system‐level outcomes including economic evaluation, resource utilization, and value‐based care measures. There is also more to discover regarding the optimal timing of nutrition consultation, which would require RCTs.

#### Question 1d

In adult patients with head and neck cancer, does longer preoperative and postoperative intervention by a dietitian compared with shorter intervention duration change progression‐free survival, overall survival, nutrition intake, time to transition to full oral diet nutrition status, weight, muscle mass, sarcopenia, myosteatosis, global quality of life, fatigue, return to work, performance status, length of stay, surgical complications, or hospital readmissions?

*Recommendation*: In adults with head and neck cancer, we recommend that the duration of dietitian intervention preoperatively and postoperatively should be individualized according to the patient's nutrition status, swallowing function, symptom burden, and clinical, psychosocial, and socioeconomic status. Intervention should continue while there remains a risk to nutrition status from nutrition‐impact symptoms, reduced dietary intake, continued indication for nutrition support, and/or other factors relating to nutrition risk. Patients undergoing certain surgical procedures, such as those with extensive surgical resections (eg, free flap reconstructions) or with dysphagia, may require dietetic support for a longer duration.

**Certainty of Evidence:** Very low/expert opinion

**Strength:** Strong

**Clinical Panel Delphi Agreement:** 100%

**External Validation Panel Delphi Agreement:** 100%

No studies met the inclusion criteria for this question.

### Rationale and discussion

In patients treated with surgery for head and neck cancer, malnutrition and low muscle mass are associated with prolonged length of hospital stay, more surgical complications, delayed wound healing, and poorer survival.^19^, ^26^ The duration of nutrition intervention before and after surgery may be important to minimize or prevent the decline in nutrition status and the associated adverse outcomes. Although no literature was found to answer this question, the current recommendation is based on this potential benefit and no known harm other than the possible inconvenience of additional appointments. This confidence that the potential benefits outweigh the potential harms necessitated a strong recommendation.

### Future research

RCTs examining the effect of longer durations of dietetic intervention before and after surgery are needed. Relevant outcomes of importance are nutrition status, hospital length of stay, surgical complications, unplanned readmission, quality of life, and survival. Studies should also stratify patients by nutrition status to account for the known adverse impact of malnutrition on these outcomes.

#### Question 2a

In adult patients with head and neck cancer receiving any treatment modality, does nutrition screening vs not screening impact progression‐free survival, overall survival, nutrition intake, nutrition status, weight, muscle mass, sarcopenia, myosteatosis, global quality of life, fatigue, return to work, performance status, treatment completion treatment interruptions, treatment toxicities, surgical complications, length of stay, or unplanned hospital admission or readmission?

*Recommendation*: In adults with head and neck cancer, we recommend that all patients be screened for malnutrition using a validated tool at their first presentation to the healthcare facility and regularly throughout treatment and recovery to facilitate timely referral for nutrition intervention. In certain patient subgroups who are at higher risk of malnutrition, screening may be bypassed if processes are established for automatic referral to a dietitian.

**Certainty of Evidence:** Very low/expert opinion

**Strength:** Strong

**Clinical Panel Delphi Agreement:** 100%

**External Validation Panel Delphi Agreement:** 100%

No studies met the inclusion criteria for this question.

### Rationale and discussion

Malnutrition is prevalent in patients with head and neck cancer.^18^ Early identification of malnutrition risk may facilitate prompt referral of at‐risk patients to a dietitian for comprehensive nutrition assessment and intervention. Therefore, screening for malnutrition risk may improve patient outcomes. Although no literature was found to answer this question, the current recommendation is based on this potential benefit and no known harm other than the possible inconvenience of participating in screening and aligns with a similar recommendation in the COSA guideline.^5^

In health services where access to a dietitian is dependent on a referral, or where dietitian resources are limited, screening using validated tools is an appropriate strategy to triage patients at risk of malnutrition to a dietitian. However, some patients with head and neck cancer (eg, those planned for combined chemoradiotherapy) are already considered at high risk of malnutrition because of treatment‐related factors. These patients may not be identified as being at risk of malnutrition through malnutrition screening processes but are at high risk of becoming malnourished. For these patients, automatic referral to a dietitian may be more appropriate, where resources permit.

### Future research

The value of screening as a triage tool is widely accepted. Randomized trials to prove that screening identifies risk of malnutrition or prompts referral to a dietitian are likely unnecessary. Instead, future research should investigate the impact of implementing nutrition screening on referral to a dietitian, specifically in health services where patients are not automatically referred to a dietitian. Screening usually involves the implementation of a new model of care, policy, or procedure. Therefore, randomization to screening vs no screening is likely to be difficult. Stepped‐wedge randomized trials or interrupted time series analysis may be appropriate study designs to assess the effect of malnutrition screening on the rate of referral to a dietitian, nutrition status, treatment completion/interruptions, surgical complications, unplanned admission or readmission, survival, cost effectiveness, and impact on the workforce.

#### Question 2b

In adult patients with head and neck cancer receiving any treatment modality, does nutrition assessment vs no nutrition assessment change progression‐free survival, overall survival, nutrition intake, nutrition status, weight, muscle mass, sarcopenia, myosteatosis, global quality of life, fatigue, return to work, performance status, treatment completion, treatment interruptions, treatment toxicities, surgical complications, length of stay, or unplanned hospital admission or readmission?

*Recommendation*: In adults with head and neck cancer, we recommend that patients undergo a comprehensive nutrition assessment if they have been screened and found to be at risk of malnutrition, if they are automatically referred through established protocols because of high malnutrition risk, or if they present with an enteral access device, either planned or already in situ. We further recommend this assessment be performed by a dietitian or other qualified nutrition professional using a tool that has been validated in the oncology population (eg, Patient‐Generated Subjective Global Assessment (PG‐SGA) or SGA).

**Certainty of Evidence:** Very low/expert opinion

**Strength:** Strong

**Clinical Panel Delphi Agreement:** 100%

**External Validation Panel Delphi Agreement:** 100%

One quasi‐experimental study^43^ met the inclusion criteria for this question (Tables 8, 9, S10, and S11).

**Table 8:** Summary of question 2b studies.

Study	Sample size	Population	Intervention	Comparator	Risk of bias
Kakati et al^43^ Quasi‐experimental	82	Adult preoperative patients with head and neck cancer	Nutrition assessment via SGA and dietetic counseling when needed (minimum 3 weeks)	No active nutrition assessment	Serious

Abbreviation: SGA, Subjective Global Assessment.

**Table 9:** Outcomes for question 2b studies.

Topic	Outcome	Study	Timing of data collection	Results
Complications	Delay in adjuvant therapy, wound infection and dehiscence, flap failure, delay in oral feeding, and prolonged hospital stay, with no difference in wound reexploration and readmission	Kakati et al^43^	From admission to 1 month post‐op	Patients in the intervention group had decreased delay in adjuvant therapy (*P* = 0.0076), wound infection and dehiscence (*P* = 0.0065), flap failure (*P* = 0.0031), delay in oral feeding (*P* = 0.0115), and prolonged hospital stay >6 days (*P* = 0.0121), with no significant difference in wound reexploration and readmission (*P* = 0.6144)
Body composition	Arm circumference, triceps skinfold, arm muscle circumference, arm fat area	Kakati et al^43^	From admission to 1 month post‐op	Patients in the intervention group had increased arm circumference (*P* = 0.00192), triceps skinfold thickness (*P* = 0.000548), arm muscle circumference (*P* = 0.013452), and arm fat area (*P* = 0.000652)
Length of stay	Hospital length of stay	Kakati et al^43^	From admission to 1 month post‐op	Patients in the intervention group had decreased hospital length of stay (*P* = 0.0121)

Abbreviation: post‐op, postoperatively.

### Rationale and discussion

One nonrandomized matched‐pair study (*n* = 82) comparing nutrition assessment via SGA with dietetic counseling when needed against a group receiving no active nutrition assessment reported decreased delay in adjuvant therapy (*P* = 0.0076), wound infection and dehiscence (*P* = 0.0065), flap failure (*P* = 0.0031), delay in oral feeding (*P* = 0.0115), a decrease in prolonged hospital stay >6 days (*P* = 0.0121), and improvement in body composition metrics.^43^ Although the bias risk was rated as “serious” for this study, nutrition assessment is a key step in the nutrition care process, required to determine nutrition status and guide interventions.^44^ It is also important for monitoring nutrition status and may therefore be a strategy to prevent nutrition decline. The current recommendation is based on this potential benefit and no known harms and aligns with a similar recommendation in the COSA guideline.^5^

### Future research

Although nutrition assessment is a key step in the nutrition care process, the optimal frequency of nutrition assessment required to minimize decline in nutrition status is unknown. RCTs are required to examine the effect of nutrition assessment frequency (before, during, and after treatment) on nutrition status in patients with head and neck cancer. Other relevant outcomes include the number of treatment completions/interruptions, surgical complications, unplanned admission/readmission, and survival.

#### Question 3a

In adult patients with head and neck cancer receiving any treatment modality, does intensive nutrition therapy designed to meet current recommendations for protein intake vs standard care change progression‐free survival, overall survival, nutrition status, weight, muscle mass, sarcopenia, myosteatosis, global quality of life, fatigue, return to work, performance status, treatment completion, treatment interruptions, treatment toxicity, surgical complications, length of stay, or unplanned hospital admission or readmission?

*Recommendation*: In adults with head and neck cancer receiving any modality of treatment, we recommend a protein intake of 1.2–1.5 g/kg/day, which would meet the needs of most patients with head and neck cancer. This may be met and maintained through one or a combination of oral intake, oral nutrition supplements, or EN to meet protein requirements and should be tailored to symptom burden and nutrition status while considering individual treatment plans and clinical, psychosocial, and socioeconomic status.

**Certainty of Evidence:** Very low/expert opinion

**Strength:** Strong

**Clinical Panel Delphi Agreement:** 100%

**External Validation Panel Delphi Agreement:** 100%

One RCT^45^ met the inclusion criteria for this question (Tables 10, 11 S12, and S13).

**Table 10:** Summary of question 3a.

Study	Sample size	Population	Intervention	Comparator	Risk of bias
de Carvalho et al^45^	49	Adults with HNC who were candidates for elective surgery	Provided 7 g of whey protein 7 h before surgery	Standard care	Some concerns

Abbreviation: HNC, head and neck cancer.

**Table 11:** Outcomes for question 3a.

Topic	Outcome	Study	Timing of data collection	Results
Complications	Post‐op complications	de Carvalho et al^45^	Time of surgery, hospital discharge	Patients in the intervention group had decreased post‐op complications (*P* < 0.001)^a^
Length of stay	Hospital length of stay	de Carvalho et al^45^	Time of surgery, hospital discharge	Patients in the intervention group had no difference in hospitalization days vs the control (*P* = 0.769)
Mortality	Hospital mortality	de Carvalho et al^45^	Time of surgery, hospital discharge	Patients in the intervention group had no difference in mortality vs the control (*P* = 0.301)

Abbreviation: post‐op, postoperative.

^a^ The control group had higher age and hypertension, and this was not accounted for in the analysis. This finding may simply reflect these between‐group baseline differences.

### Rationale and discussion

Muscle loss is common in patients with head and neck cancer, and observational studies show it is associated with poor outcomes.^17^, ^18^ Adequate protein is important to maintain muscle mass or minimize muscle loss. The one study that met our inclusion criteria (*n* = 49) examined the presurgical provision of whey protein against standard care and reported decreased postoperative complications in the whey protein group (*P* < 0.001), although unmanaged between‐group differences at baseline make this statistic difficult to interpret.^45^ It was rated as having “some concerns” for bias risk. Further, studies that did not meet our inclusion criteria because of differences in design, time period, or rigor of intervention, but that examined protein delivery at different levels in patients with cancer, have not reported any harms in protein delivery at these levels. One meta‐analysis examined eight studies in patients with cancer with a high prevalence of sarcopenia. Five of these were in patients with head and neck cancer. One RCT and four pre‐post studies in head and neck cancer supported the benefit of protein for maintaining muscle mass. The authors found that, taking all eight studies into account, lean muscle wasting during treatment was found in patient groups that maintained an intake of <1.2 g/day of protein. Groups receiving >1.4 g/day experienced lean muscle maintenance.^46^ It is important to note that the inclusion criteria for the systematic review only required protein to have been tracked between groups. Protein did not need to be the primary intervention, and co‐interventions were not considered. Therefore, we cannot be confident that protein was the cause of the outcome, but it does imply that protein at those levels was not harmful. Although the effect of supplementing protein in these patients is unknown, one meta‐analysis of 11 retrospective studies in head and neck cancer found sarcopenia to be associated with decreased overall survival and increased relapse‐free survival.^47^ Although neither of these reviews can be considered definitive and no studies specifically designed to answer this question were found, the current recommendation has no reported harms, and the idea that it may be somewhat protective against severe muscle wasting is biologically plausible, supported by these meta‐analyses, and is consistent with the COSA guideline and other guidelines.^5^, ^48^

### Future research

The effect of intensive nutrition therapy, using one or a combination of oral intake, oral nutrition supplements, or EN, on protein intake has not been established. RCTs designed to achieve and maintain protein intake using dietary counseling, escalating to oral nutrition supplements and/or EN as clinically indicated, are needed to assess the effect on muscle mass, nutrition status, treatment completion/interruptions, treatment toxicities, surgical complications, postoperative length of stay, unplanned hospital admissions, and survival. The exploration of protein intakes up to 2 g/kg/day has occurred in other cancer populations.^49^

#### Question 3b

In adult patients with head and neck cancer receiving any treatment modality, does intensive nutrition therapy designed to meet current recommendations for energy intake vs standard care change progression‐free survival, overall survival, nutrition status, weight, muscle mass, sarcopenia, myosteatosis, global quality of life, fatigue, return to work, performance status, treatment completion, treatment interruptions, treatment toxicity, surgical complications, length of stay, or unplanned hospital admission or readmission?

*Recommendation*: In adults with head and neck cancer receiving any modality of treatment, we recommend an energy intake of ≥30 kcal/kg/day. This may be met and maintained through one or a combination of oral intake, oral nutrition supplements, or EN to meet energy requirements and should be tailored to symptom burden and nutrition status while considering individual treatment plans and clinical, psychosocial, and socioeconomic status. Nutrition status should be monitored regularly to determine if energy intake is sufficient, noting that sufficient energy intake is also important to ensure protein intake is used for the preservation of muscle mass.

**Certainty of Evidence:** High

**Strength:** Strong

**Clinical Panel Delphi Agreement:** 100%

**External Validation Panel Delphi Agreement:** 100%

Seven studies met the inclusion criteria for this question.^24^, ^50^, ^51^, ^52^, ^53^, ^54^, ^55^ Five demonstrated a significant benefit in the intervention compared with the control group,^50^, ^51^, ^53^, ^54^, ^55^ with one study reporting less chemotherapy and radiotherapy interruptions in the intervention group^50^; one reporting less flap failure, flap dehiscence, and neck hematoma and decreased hospital length of stay^54^; three studies reporting better weight/body mass index (BMI) maintenance in the intervention group^51^, ^53^, ^55^; two reporting improved measures of body composition^51^, ^55^; and one reporting improved PG‐SGA scores in the intervention group.^53^ Of these, three had “some concerns.” Two were “low” risk of bias and two quasi‐experimental designs were reported at “serious” risk for bias. No studies reported worse outcomes in the intervention vs the control (Tables 12, 13, and S14–S16).

**Table 12:** Summary of question 3b studies.

Study	Sample size	Population	Intervention	Comparator	Risk of bias
Huang et al^50^	119	Adults with advanced nasopharyngeal cancer treated with chemoradiation	Prophylactic ONS	Standard care	Some concerns
Prasad et al^24^ Quasi‐experimental	78	Adult patients who underwent different types of laryngeal surgeries	Oral feeds initiated POD 2	Oral feeds initiated POD 10	Serious
Jiang et al^51^	63	Adult, surgically treated patients with HNSCC	ONS 2 weeks before RT	Standard care	Low
Laskar et al^52^	84	Adult patients with HNC	Prophylactic NGT placed at least 1 day before RT	Reactive NGT placement as needed	Low
Dou et al^53^	52	Adult patients with nasopharyngeal carcinoma undergoing CCRT	ONS from treatment initiation to week 2	No ONS	Some concerns
Kerawala et al^54^ Quasi‐experimental	200	Adult patients with HNC undergoing head and neck reconstruction	Early‐feeding group: start fluids/soft diet POD 1	Late‐feeding group: 5 days nil per os before initiating progressive feeding	Serious
Abouegylah et al^55^	30	Adult patients with HNC undergoing chemotherapy.	Received 200 ml of ONS twice daily	No ONS	Some concerns

Abbreviations: CCRT, concurrent chemoradiotherapy; HNC, head and neck cancer; HNSCC, head and neck squamous cell carcinoma; NGT, nasogastric tube; ONS, oral nutrition supplement; POD, postoperative day; RT, radiotherapy.

**Table 13:** Outcomes data for question 3b studies.

Topic	Outcome	Study	Timing of data collection	Results
Complications	Fistulas	Prasad et al^24^	Unclear. Presumably the end of their hospital stay	No significant differences were found between groups for fistulas in the larynx (*P* = 1.0), hypopharynx (*P* = 1.0), or laryngopharynx (*P* = 0.23)
	Wound infection, flap necrosis, aspiration	Prasad et al^24^	Unclear. Presumably the end of their hospital stay	No differences were found between groups for wound infection (*P* = 1.0), flap necrosis (*P* = 0.61), or aspiration (*P* = 0.61)
	Flap failure, neck hematoma/seroma, flap dehiscence, neck fistula	Kerewala et al, 2001	Baseline to hospital discharge	In the intervention group (early feeding), the incidence of flap failure was 1% compared with 1.5% in the control group (late feeding, *P* < 0.0001). Neck hematoma or seroma occurred in 2% of the intervention group vs 3% of the control group (*P* < 0.0001). Flap dehiscence was observed in 4% of the intervention group and 5% of the control group (*P* < 0.0001). The incidence of neck fistula was the same in both groups at 4% (*P* = NS).
Length of stay	Hospital length of stay	Prasad et al^24^	Unclear. Presumably the end of their hospital stay	Hospital length of stay is significantly longer in control vs intervention groups (χ^2^ = 15.34, *P* = 0.0015)
	Hospital length of stay	Kerewala et al, 2001	Baseline to hospital discharge	The intervention group had decreased length of hospital stay vs the control (11.6 vs 20.6 days, *P* < 0.01)
Nutrition status	NRS‐2002 and PG‐SGA	Huang et al^50^	Six time points over 3 months	No difference in nutrition status on either measure between groups
Quality of life	EORTC QLQ‐C30	Huang et al^50^	Six time points over 3 months	No difference in quality of life between groups
	EORTC QLQ‐C30 and QLQ‐H&N35	Jiang et al^51^	2 weeks before RT, beginning of RT, 3 weeks after starting RT, end of RT, 2 weeks post‐RT	No difference reported for any domain
Treatment interruptions	Chemotherapy interruptions	Huang et al^50^	Six time points over 3 months	Chemotherapy interruption was less in the intervention group vs control (10.34% vs 28.57%, *P* = 0.01)
	RT interruption	Huang et al^50^	Six time points over 3 months	RT interruption was less in the intervention group vs control (0% vs 7.14%, *P* = 0.04).
	RT interruption	Jiang et al^51^	2 weeks before RT through 2 weeks after RT	No difference reported between groups
Treatment toxicities	Mucositis	Huang et al^50^	Six time points over 3 months	The intervention group had lower mucositis for both stage 3/4 and stage ½, but this was not statistically significant (*P* = 0.34 and 0.45, respectively)
	Oral mucositis (grades 3–4), nausea, xerostomia, neutropenia	Jiang et al^51^	2 weeks before RT through 2 weeks after RT	No differences were reported for these outcomes
	Dermatitis, mucositis, xerostomia, nausea, vomiting, neutropenia, fever, pain, diarrhea, hyponatremia, hypokalemia, hypomagnesemia	Laskar et al^52^	Baseline to 6 months posttreatment (10 time points)	No difference reported for any toxicity between groups
	Oral mucositis, radiation dermatitis, hematologic, and gastrointestinal issues	Dou et al^53^	Baseline and weeks 1, 2, and 3	No differences were reported for treatment toxicities between groups
	Nausea, dysphagia, food intake dermatitis, mucositis grade, and xerostomia	Abouegylah et al^55^	Weeks 1, 3–4, and 6–7	No differences were found between groups for radiation treatment toxicities except that the ONS group ate more solid food than the no‐ONS group (*P* = 0.058)
Weight	Percent with >5% or >10% weight loss	Huang et al^50^	Six time points over 3 months	No difference in weight loss on either measure between groups
	Weight loss/BMI	Jiang et al^51^	2 weeks before RT, beginning of RT, 3 weeks after starting RT, end of RT, 2 weeks after RT	The intervention group lost significant less weight (or gained weight) vs the control at every time point (*P* < 0.001) BMI was significantly higher in the intervention vs the control starting by 3 weeks after starting RT and stayed that way through 3 weeks post‐RT (*P* < 0.05)
	Weight	Laskar et al^52^	Baseline to 6 months posttreatment (10 time points)	No differences between groups
	Weight/BMI	Dou et al^53^	Baseline and weeks 1, 2, and 3	No difference reported for weight but intervention group had better BMI maintenance at week 2 (*P* = 0.024)
	Weight loss/BMI loss	Abouegylah et al^55^	Weeks 1, 3–4, and 6–7	No differences between groups, but the control group lost significant weight and BMI over 7 weeks (*P* < 0.001), whereas the intervention group did not
Nutrition intake	Energy intake from protein	Jiang et al^51^	2 weeks before RT, beginning of RT, 3 weeks after starting RT, end of RT, 2 weeks after RT	The intervention group received more protein relative to the control, but this was only significant at the beginning of RT (*P* = 0.028)
	Energy intake	Jiang et al^51^	2 weeks before RT, beginning of RT, 3 weeks after starting RT, end of RT, 2 weeks after RT	The intervention group received more energy intake relative to the control, but this was only significant at 2 weeks after RT (*P* = 0.011)
Nutrition status	PG‐SGA	Jiang et al^51^	2 weeks before RT, beginning of RT, 3 weeks after starting RT, end of RT, 2 weeks after RT	No difference was reported between groups
	PG‐SGA	Dou et al^53^	Baseline and weeks 1, 2, and 3	Intervention group had higher PG‐SGA scores relative to control by the end of the CCRT (*P* = 0.053)
	NRS‐2002 PG‐SGA	Huang et al^50^	Six time points over 3 months	No difference in nutrition status on either measure between groups.
Body composition	Calf circumference, FFM, FFMI, SMM, FM	Jiang et al^51^	2 weeks before RT, beginning of RT, 3 weeks after starting RT, end of RT, 2 weeks after RT	Calf circumference, FFM, FFMI, SMM, and FM improved in the intervention vs the control at all time points but did not achieve significance for most time points. Calf circumference was significantly higher in the intervention at end of RT (*P* = 0.049). FM was higher at 3 weeks after starting RT (*P* < 0.001) and 2 weeks after ending RT (*P* = 0.011)
	FM, FFM	Abouegylah et al^55^	Weeks 1, 3–4, and 6–7	No differences between groups, but the control group lost significant FM and FFM over 7 weeks (*P* < 0.001), whereas the intervention group did not
Survival	Disease‐free and overall survival	Jiang et al^51^	3 years	Three‐year disease‐free survival for patients in the intervention vs control was 74.1% vs 67.4% (*P* = 0.315), respectively. Three‐year overall survival rates in the CNI and ENI groups were 77.1% vs 71.0% (*P* = 0.722)
	Progression‐free and overall survival	Laskar et al^52^	Baseline to 6 months posttreatment (10 time points)	No differences reported between groups

Abbreviations: BMI, body mass index; CCRT, concurrent chemoradiotherapy; CNI, conventional nutrition intervention; ENI, enteral nutrition intervention; EORTC, European Organisation for Research and Treatment of Cancer; FFM, fat‐free mass; FFMI, fat‐free mass index; FM, fat mass; NRS‐2002, Nutrition Risk Screening 2002; NS, not significant; ONS, oral nutrition supplement; PG‐SGA, Patient‐Generated Subjective Global Assessment; QLQ‐C30, Quality of Life Questionnaire–Core 30; QLQ‐H&N35, Quality of Life Questionnaire–Head and Neck 35; RT, radiotherapy; SMM, skeletal muscle mass.

### Rationale and discussion

Malnutrition is common in patients with head and neck cancer and is associated with poor outcomes.^17^, ^18^ Observational studies show that adequate energy intake is vital for maintaining nutrition stores and minimizing a decline in nutrition status.^56^ With energy needs repleted, protein utilization strategies can shift to prioritize maintenance of lean muscle mass. One small observational study in 41 patients with head and neck cancer found that an energy intake of 30 kcal/kg/day was required to attenuate muscle loss, whereas an energy intake of 25 kcal/kg/day was associated with muscle loss.^56^ The current recommendation is based on this potential benefit, along with clear evidence of benefit concerning weight maintenance and no evidence of harm. It is also consistent with the COSA guideline and other guidelines.^5^, ^48^

### Future research

The effect of intensive nutrition therapy on energy intake, using one or a combination of oral intake, oral nutrition supplements, or EN, has not been established. Randomized trials designed to achieve and maintain energy intake using dietary counseling and escalating to oral nutrition supplements and/or EN if clinically indicated, are needed. Relevant outcomes for these trials include energy intake, muscle mass, nutrition status, treatment completion/interruptions, treatment toxicities, surgical complications, postoperative length of stay, unplanned hospital admissions, and survival.

#### Question 4a

In adult patients with head and neck cancer receiving any treatment modality, does estimating protein requirements based on an alternate body weight or composition vs standard care (actual weight) change progression‐free survival, overall survival, nutrition status, weight, muscle mass, sarcopenia, myosteatosis, global quality of life, fatigue, return to work, performance status, treatment completion, treatment interruptions, treatment toxicity, surgical complications, length of stay, or unplanned hospital admissions or readmission?

*Recommendation*: In adults with head and neck cancer receiving any treatment modality, owing to insufficient evidence at present to demonstrate a benefit from individualizing protein requirements based on body composition, we suggest estimating protein requirements based on actual body weight. However, the risk of overestimating protein requirements in patients with obesity is higher. An acceptable solution to this may be to use the higher end of ideal body weight or to use actual body weight while using clinical judgment to determine if the resulting target protein amount is achievable. We recommend ongoing monitoring of nutrition intake alongside nutrition status, muscle mass, muscle strength, and physical performance as an indication of the adequacy of protein intake.

**Certainty of Evidence:** Very low/expert opinion

**Strength:** Weak

**Clinical Panel Delphi Agreement:** 100%

**External Validation Panel Delphi Agreement:** 100%

No studies met the inclusion criteria for this question. See Question 4b for discussion.

The rationale, discussion, and future research directions for this recommendation are included in Question 4b because of similarities.

#### Question 4b

In adult patients with head and neck cancer receiving any treatment modality, does estimating energy requirements based on an alternate body weight or composition vs standard care (actual weight) change progression‐free survival, overall survival, nutrition status, weight, muscle mass, sarcopenia, myosteatosis, global quality of life, fatigue, return to work, performance status, treatment completion, treatment interruptions, treatment toxicity, surgical complications, length of stay, or unplanned hospital admission or readmission?

*Recommendation*: In adults with head and neck cancer receiving any treatment modality, we suggest estimating energy requirements based on actual body weight because of insufficient evidence at present to demonstrate a benefit from individualizing energy requirements based on body composition. However, the risk of overestimating energy requirements in patients with obesity is higher. In this situation, either ideal body weight or actual body weight may be used with clinical judgment to determine if the resulting target energy requirement is achievable. We recommend ongoing monitoring of nutrition intake alongside weight, nutrition status, muscle mass, muscle strength, and physical performance as an indication of adequacy of energy intake.

**Certainty of Evidence:** Very low/expert opinion

**Strength:** Weak

**Clinical Panel Delphi Agreement:** 100%

**External Validation Panel Delphi Agreement:** 100%

No studies met the inclusion criteria for this question.

### Rationale and discussion

Current methods to estimate protein requirements are based on body weight and do not consider muscle mass or body composition. Observational studies show that protein requirements that are estimated using body weight may underestimate or overestimate protein requirements, particularly in patients with obesity.^57^ Protein intake that is individualized to body composition may prevent underfeeding or overfeeding of protein. Inadequate energy intake may lead to the utilization of muscle protein as an energy source and result in muscle loss. However, a narrative review suggests excess energy intake leads to increased fat mass, which may increase the risk of cancer recurrence and other chronic health conditions.^58^ Individualizing estimation of energy requirements may, therefore, be important to prevent adverse changes to body composition (eg, muscle loss or increased fat mass) or exacerbation of existing adverse body composition. However, individualizing protein and energy requirements relies on accurate assessment of body composition, in particular muscle mass, and most assessment techniques have limitations at this time.^59^ Inaccurate assessment of muscle mass could result in underestimation or overestimation of protein and/or energy requirements, with underestimation having an undesirable effect on muscle loss and overestimation having an undesirable effect on fat mass. The current recommendation to use actual body weight or ideal body weight in obesity is based on an effort to avoid this potential harm.

It should be noted that the method to calculate ideal body weight for patients with obesity may vary regionally. In the United States, it is common to use the Hamwi equation.^60^ In Australia, it is common to define ideal body weight as the weight at the upper end of the range for BMI within the healthy reference range, noting that ranges appropriate to ethnicity should be used. Patients with head and neck cancer who are underweight are likely to require the higher end of the recommended range for protein intake (1.5 g/kg/day). If weight gain occurs, requirements may need to be adjusted.

In patients with head and neck cancer who are underweight, we have not suggested alternatives to using actual body weight. This is because using actual body weight to estimate energy requirements is expected to lead to weight gain.

### Future research

Individualizing protein and energy requirements based on body composition should be investigated in randomized trials to determine the effect on muscle mass, fat mass, nutrition status, treatment toxicities, treatment completion/interruptions, surgical complications, postoperative length of stay, unplanned hospital admissions, and survival. There is potential for findings to vary depending on which body composition assessment technique is used. Therefore, to ensure comparability across studies, a discussion needs to occur regarding which techniques should be used. Priority should be given to techniques that are both feasible in clinical practice and validated in a cancer population.

#### Question 5

In adult patients with head and neck cancer receiving any treatment modality, does gastrostomy feeding (via PEG or radiologically inserted gastrostomy [RIG]) vs nasogastric tube (NGT) feeding change progression‐free survival, overall survival, nutrition intake, nutrition status, weight, muscle mass, sarcopenia, myosteatosis, dysphagia, incidence of stricture, fistula development, global quality of life, fatigue, return to work, performance status, treatment completion, feeding tube dependence, time of transition to full oral diet, treatment interruptions, treatment toxicity, surgical complications, length of stay, or unplanned hospital admission or readmission?

*Recommendation*: In adults with head and neck cancer receiving any treatment modality, we suggest that the decision to place a PEG or RIG tube vs an NGT is made through discussion among interdisciplinary team members including a dietitian or another member with nutrition training. The decision regarding the type of enteral access device should be based on the clinical situation (including tumor location and stage), symptom burden (especially preexisting dysphagia), treatment plan, psychosocial situation, and the anticipated duration of enteral feeding. If EN is indicated, feeding via PEG/RIG may be more appropriate when anticipated for longer durations (commonly >4–6 weeks); otherwise, an NGT should be considered.

**Certainty of Evidence:** Moderate

**Strength:** Weak

**Clinical Panel Delphi Agreement:** 100%

**External Validation Panel Delphi Agreement:** 100%

Four studies met the inclusion criteria for this PICOT question. Three studies demonstrated a significant benefit in the intervention compared with the NGT feeding control group.^61^, ^62^, ^63^ Three studies reported less weight loss,^61^, ^62^, ^63^ two found less tube dislodgement,^61^, ^62^ one reported less surgical site dehiscence,^63^ one reported less infections,^62^ one found improved quality of life,^62^ one reported less altered body image,^61^ one reported less loss of mid‐upper arm circumference,^62^ and one study reported higher triceps skinfold thickness.^61^ The remaining study found no significant differences between the two groups for any outcome (Tables 14, 15, S17, and S18). Although no studies favored the control, one study reported a longer duration of enteral feeding, more pain, and more inconvenience in the intervention group.^61^ All four studies were rated as having some concerns for risk of bias. This position is also consistent with observational studies that did not meet our inclusion criteria but found that using protocols to guide decisions regarding the route of enteral feeding improved clinical outcomes.^21^, ^22^, ^65^

**Table 14:** Summary of question 5 studies.

Study	Sample size	Population	Intervention	Comparator	Risk of bias
Axelsson et al^64^	134	Adult patients initially diagnosed with advanced oral, hypopharyngeal, oropharyngeal, or nasopharyngeal cancer or malignant neck nodes from an unknown primary cancer treated with curative intent	Patients received prophylactic PEG	Standard care	Some concerns
Corry et al^61^	33	Adult patients with squamous cell carcinoma of the head and neck planned for radiotherapy or chemoradiation with curative intent	PEG	NGT	Some concerns
Sadasivan et al^62^	100	Adult patients with head and neck malignancy	Patient had a PEG	Patients had an NGT	Some concerns
Tabrizi et al^63^	40	Adult patients with stage III or IV oral squamous cell carcinoma who underwent hemimandibulectomy and a selective neck dissection	PEG for 4 weeks	NGT for 4 weeks	Low

Abbreviations: NGT, nasogastric tube; PEG, percutaneous endoscopic gastrostomy.

**Table 15:** Outcomes data for question 5 studies.

Topic	Outcome	Study	Timing of data collection	Results
Complications	Tube dislodgment	Sadasivan et al^62^	Baseline to 6 weeks	The PEG group had no tube dislodgment vs 16 patients in the NGT group (*P* < 0.001)
	Tube dislodgment	Corry et al^61^	Tube insertion to week 6	The PEG group had no tube dislodgment vs 12 patients in the NGT group (*P* = 0.0001)
	Surgical site dehiscence	Tabrizi et al^63^	Baseline to week 4	The PEG group had less surgical site dehiscence compared with the NGT group (15% vs 50%, *P* = 0.04)
Dysphagia	CTC v2.0 grade 3 dysphagia at 6 months post‐RT	Corry et al^61^	6 months	No difference in CTC v2.0 grade 3 dysphagia at 6 months post‐RT (4/15 PEG vs 1/18 NGT, *P* = 0.15)
	Oral intake scale Swallowing Swallowing liquids Swallowing solids	Axelsson et al^64^	Inclusion, 12 months, 24 months, 8 years	No between‐group difference was found for any variable of oral intake or swallowing at any time point
Feeding tube dependence	Median duration of tube feeding use	Corry et al^61^	Tube insertion to week 6	PEG group had higher duration of tube feeding than NGT group (139 vs 66 days, *P* = 0.0006)
Infection	Total infections	Sadasivan et al^62^	Baseline to 6 weeks	The PEG group had fewer infections than the NGT group (2 vs 28, *P* < 0.001)
	Total infections	Tabrizi et al^63^	Baseline to week 4	The PEG group had fewer infections than the NGT group (5% vs 35%, *P* = 0.044)
	Infections	Corry et al^61^	Tube insertion to week 6	Twenty‐seven percent of PEG group experienced PEG site infections. Chest infections did not differ between the groups (*P* = 0.722)
Mortality	Overall survival	Axelsson et al^64^	Followed 12 years Checked at 2, 5, and 10 years	No between‐group differences were found for overall survival at any time point
QOL	EORTC QLQ‐C30 EORTC QLQ‐H&N35	Axelsson et al^64^	Inclusion, 12 months, 24 months, 8 years	No between‐group differences were found in any domain for QOL
	Modified QOL assessment	Sadasivan et al^62^	Baseline and 6 weeks	The PEG group fared significantly better in every QOL domain (pain, learning to use, inconvenience, uncomfortable feeds, altered body image, family life, and social activities; *P* < 0.01 for each domain)
	Modified QOL assessment	Corry et al^61^	Tube insertion to week 6	In general, patients in the PEG group experienced more pain (*P* = 0.03) and more inconvenience (*P* = 0.02) but less altered body image (*P* = 0.05) than the NGT group
Weight and body composition	Mean weight loss	Tabrizi et al^63^	Baseline to week 4	The PEG group lost less weight than the NGT group (5.3 vs 7.9 kg, *P* = 0.001)
	BMI Median weight	Axelsson et al^64^	Inclusion, 12 months, 24 months, 8 years	No between‐group differences were found for BMI at any time point
	6‐week % mean weight change	Sadasivan et al^62^	Baseline and 6 weeks	The PEG group lost less weight compared with the NGT group (−3.31% vs −11.0%, *P* < 0.001)
	6‐week absolute weight	Corry et al^61^	Week 6	No difference was found in PEG vs NGT groups (59 vs 62 kg, *P* = 0.17)
	6‐week weight change	Corry et al^61^	Tube insertion to week 6	PEG group lost less weight than NGT group (1.29 vs 3 kg, *P* = 0.001)
	6‐week % mean MAC change	Sadasivan et al^62^	Baseline and 6 weeks	The PEG group lost less MAC compared with the NGT group (−3.37% vs –8.07%, *P* < 0.001)
	6‐week MUAC change	Corry et al^61^	Tube insertion to week 6	No difference was found in PEG vs NGT groups (295 mm vs 283 mm, *P* = 0.25)
	Triceps skinfold thickness	Corry et al^61^	Week 6	PEG group had higher triceps skinfold thickness than NGT group (13.5 vs 9.5 mm, *P* = 0.03)

Abbreviations: BMI, body mass index; CTC, Common Terminology Criteria; EORTC, European Organisation for Research and Treatment of Cancer; MAC, mid‐arm circumference; MUAC, mid‐upper arm circumference; NGT, nasogastric tube; PEG, percutaneous endoscopic gastrostomy; QLQ‐C30, Quality of Life Questionnaire–Core 30; QLQ‐H&N35, Quality of Life Questionnaire–Head and Neck 35; QOL, quality of life; RT, radiotherapy.

### Rationale and discussion

Patients with head and neck cancer are at high risk of dysphagia, odynophagia, and mucositis owing to the tumor location and treatment.^66^ Enteral feeding is required to meet energy and protein requirements when patients are unable to achieve sufficient oral intake. Enteral feeding using a PEG or RIG is typically used when enteral feeding is expected to be needed for longer durations, whereas NGT feeding is typically used when a shorter duration is anticipated. PEG and RIG tube insertion is generally safe; however, a narrative review indicates complications are reported to occur in 5%–40% of cases.^67^ The most common complication is wound infection, which is typically minor, although major complications requiring surgical intervention or that are life‐threatening may occur.^67^ A narrative review suggests NGT insertion is most commonly associated with minor complications, including discomfort, sinusitis, or epistaxis, and long‐term use may result in irritation of the gastric lining, nasal pressure ulcers, and gastrointestinal bleeding.^68^ Confirmation of placement must occur before initiating EN.^68^ The current weak recommendation is based on these considerations, along with some evidence of benefit for PEG feeding from the literature, balanced with the potential harms, and aligns with similar recommendations in the COSA guidelines.^5^

Observational studies suggest that a longer duration of enteral feeding is likely to be required in patients requiring extensive surgical resection, those planned for oral or bilateral radiotherapy with concurrent chemotherapy, or patients with pre‐existing dysphagia.^21^, ^69^ Patients who have experienced severe unintentional weight loss (>10% in 6 months), severe malnutrition, or pre‐existing poor oral intake may also be at high risk of needing a longer duration of enteral feeding.^21^ While this recommendation explicitly states PEG and RIG tubes, it applies to all types of gastrostomy tubes.

### Future research

Randomizing patients to PEG compared with NGT may be challenging when clear indications exist for one vs the other, but RCTs may be warranted in patients for whom indication for an NGT or PEG is equal. Further studies should examine the pre and post effect of implementing protocols or algorithms to guide decisions on the enteral access device as treatment modalities and their toxicity profiles evolve. Outcomes that should be examined include nutrition status, treatment completion/interruptions, treatment toxicities, unplanned hospital admissions, dysphagia, and survival.

#### Question 6

In adult patients with head and neck cancer receiving any treatment modality, does more frequent speech pathology intervention compared with the standard of care change the time to transition to full oral diet, progression‐free survival, overall survival, nutrition intake, nutrition status, weight, muscle mass, sarcopenia, myosteatosis, dysphagia, global quality of life, fatigue, return to work, performance status, treatment completion feeding tube dependence, incidence of stricture, fistula development, treatment interruptions, treatment toxicity, surgical complications, length of stay, or unplanned hospital admission or readmission?

*Recommendation*: In adults with head and neck cancer receiving any treatment modality, we recommend consultation by a speech pathologist before treatment (surgery or radiotherapy with or without chemotherapy) for baseline assessment and education if the treatment is likely to affect swallowing function or in the case of pre‐existing dysphagia. We recommend that the frequency of consultation by a speech pathologist during and after radiotherapy (with or without chemotherapy) and after surgery be guided by the treatment plan as well as the severity of dysphagia and other treatment toxicities. Additional considerations include clinical, psychosocial, and socioeconomic status. Interventions should be tailored to reduce dysphagia risk, minimize malnutrition, and improve quality of life. These interventions may include the maintenance of oral intake throughout radiotherapy (if safe to do so), prophylactic or therapeutic swallowing exercises, texture modification, swallowing maneuvers, compensatory strategies, and education.

**Certainty of Evidence:** Moderate

**Strength:** Strong

**Clinical Panel Delphi Agreement:** 100%

**External Validation Panel Delphi Agreement:** 100%

Eight studies met the inclusion criteria for this question.^70^, ^71^, ^72^, ^73^, ^74^, ^75^, ^76^, ^77^ Five studies demonstrated a significant benefit in the intervention group compared with the control.^71^, ^72^, ^73^, ^74^, ^77^ Three reported better swallow function,^71^, ^72^, ^74^ three found improved mouth opening,^72^, ^74^, ^77^ one reported improved normalcy of diet at 3 and 6 months,^73^ and one reported improved self‐reported functional oral intake in the intervention group.^71^ One study favored the control, finding improved ability to swallow^70^ in the control group. The remaining study found no significant differences between the two groups.^75^ One study was rated as having a “low” risk of bias. One quasi‐experimental design was rated as having “serious” risk of bias. The remaining studies were rated as having some concerns or high risk of bias (Tables 16, 17, and S19–S21).

**Table 16:** Summary of question 6 studies.

Study	Sample size	Population	Intervention	Comparator	Risk of bias
Ahlberg et al^70^ Quasi‐experimental	374	Patients diagnosed with HNC	Patients were examined by the SLP before RT and 3 months after completion of therapy. They were instructed on mobility exercises for the tongue and larynx and given a regimen	Control group did not meet with SLP or PT	Serious
Balbinot et al^71^	30	Adults and older adults in Brazil who were surgically treated for tongue cancer	30 min of speech therapy, once a week, over 1 month, and instruction to perform care and exercises at home	No speech therapy	Some concerns
Carnaby‐Mann et al^72^	41	Adult patients with oropharyngeal HNC with planned external beam RT	Standardized high‐intensity swallowing therapy (“pharyngocize”)	A sham swallow group and a true control group	Some concerns
Kotz et al^73^	26	Patients scheduled for CRT for newly diagnosed HNC	Prophylactic swallowing exercises initiated before the start of radiation and continued for the duration of CRT	Standard care including SLP referral	Some concerns
Messing et al^74^	60	Patients with biopsy‐proven stage III or IV squamous cell carcinoma of the oral, oropharynx, pharynx, or larynx regions undergoing CRT	Prophylactic swallowing exercises twice daily	No exercises, TheraBite (Atos Medical) prophylactically as standard of care	Some concerns
Van den Berg et al^36^	120	Adult patients with stage II–IV HNC treated with postoperative (chemo)radiation	Dietetic counseling plus swallow therapy	Dietetic counseling plus standard care	Some concerns
Petersson et al^76^	89	Adult patients with HNC offered RT with curative intent	Swallowing or mouth opening exercises	Standard care	Low
Guillen‐Sola et al^77^	52	Adult patients with HNC	Patients began swallow training rehabilitation program 2 weeks early	Patients began swallow training rehabilitation program 2 weeks later	Some concerns

Abbreviations: CRT, chemoradiotherapy; HNC, head and neck cancer; PT, physical therapist; RT, radiotherapy; SLP, speech‐language pathologist.

**Table 17:** Outcomes data for question 6 studies.

Topic	Outcome	Study	Timing of data collection	Results
Dysphagia	Percentage able to swallow all consistencies of food	Ahlberg et al^70^	Diagnosis to 6 months	Control group had improved ability to swallow all consistencies of food compared with SLP group (58% vs 35%, *P* < 0.001)
	Speech problems Reduced ability to open mouth	Ahlberg et al^70^	Diagnosis to 6 months	Control group had lower percentage of speech problems (*P* = 0.001) and more ability to open mouth (*P* = 0.018) compared with the SLP group
	FEES	Balbinot et al^71^	Before and after speech therapy	The intervention group improved more than the control group in FEES (*P* < 0.001)
	FEES	Petersson et al^76^	Pre‐RT, 1 month post‐RT	No differences between groups
	Interincisal mouth opening (MIO) and Penetration‐Aspiration Scale (PAS)	Petersson et al^76^	Pre‐RT, 1 month post‐RT	No differences between groups
	Modified barium swallow study	Messing et al^74^	Baseline and 3, 6, 12, and 24 months	No significant between‐group differences
	Oromotor function	Messing et al^74^	Baseline and 3, 6, 12, and 24 months	At month 6, the intervention group had an improvement in oromotor function (*P* = 0.04) compared with the control group and an improvement in incisal opening at 24 months (*P* = 0.04). All other measures and time points were insignificant
	Oral pharyngeal swallow efficiency	Messing et al^74^	Baseline and 3, 6, 12, and 24 months	No significant between‐group differences
	Mann assessment of swallowing ability	Carnaby‐Mann et al^72^	Posttherapy and 6 months	Functional swallowing ability deteriorated less in the intervention group compared with usual care (*P* = 0.03) or sham group (*P* = 0.06)
	Mouth opening	Carnaby‐Mann et al^72^	Posttherapy and 6 months	The intervention group had greater ability to open mouth by month 6 compared with sham group and usual care (*P* = 0.047)
	Dysphagia severity via Normalcy of Food Intake Scale for Head and Neck Logopedic Part/MD Anderson Dysphagia Inventory/swallowing velocity/swallowing volume	Van den Berg et al^36^	Baseline, week 10, and week 30	No significant differences between groups on any domain of any variable
	PSS‐H&N and FOIS	Kotz et al^73^	Pre‐CRT; post‐CRT; and 3, 6, 9, and 12 months	Improvement was reported in the intervention group vs the control group for Eating in Public, Normalcy of Diet, and FOIS at months 3 and 6 (*P* < 0.05). No significant difference at any other time point
	Swallowing function (DOSS, DIGEST, PAS/BRS)	Guillen‐Sola et al^77^	Pre‐RT; end of RT; 3, 6, and 12 months; post‐RT	No difference in any measure between groups, but the intervention was directionally favored
	Mouth opening (MIO)	Guillen‐Sola et al^77^	Pre‐RT; end of RT; 3, 6, and 12 months; post‐RT	The intervention group preserved better mouth opening from baseline to end of RT (*P* = 0.037) but was insignificant for other time points
Mortality	2‐year survival	Ahlberg et al^70^	Diagnosis to 2 years	No difference in 2‐year survival between groups (*P* = 0.49)
Nutrition intake	PSS‐H&N Normalcy of Diet and Normalcy of Food Intake Scale for Head and Neck Dietetic Part	Van den Berg et al^36^	Baseline, week 10, week 30	No significant differences between groups for either measure
	Self‐reported eating and drinking	Petersson et al^76^	Pre‐RT, 1 month post‐RT	No differences between groups
Quality of life	FOIS	Messing et al^74^	Baseline and 3, 6, 12, and 24 months	No between‐group difference was found at any time point
	FOIS	Carnaby‐Mann et al^72^	Posttherapy and 6 months	No significant difference between groups
	EORTC QLQ‐C30 EORTC QLQ‐H&N35	Messing et al^74^	Baseline and 3, 6, 12, and 24 months	At month 3, the intervention group had improvements in global health (*P* = 0.05) and social eating (*P* = 0.05). All other measures and time points were insignificant
	EORTC HADS	Ahlberg et al^70^	Diagnosis to 6 months	No differences were found for any domain of EORTC or HADS
	DHI FOIS	Balbinot et al^71^	Before and after speech therapy	The intervention group improved more than the control group in DHI (*P* < 0.001) and FOIS (*P* < 0.001)
	EORTC QLQ‐C30	Petersson et al^76^	Pre‐RT, 1 month post‐RT	No differences between groups for any domain
	EORTC QLQ‐C30	Guillen‐Sola et al^77^	Pre‐RT; end of RT; 3, 6, and 12 months; post‐RT	No difference in any measure between groups, but the intervention was directionally favored
Weight/nutrition status	Mean weight loss	Carnaby‐Mann et al^72^	Posttherapy and 6 months	No significant difference between groups
	Mean weight loss	Van den Berg et al^36^	Baseline, week 10, week 30	No significant difference between groups
	Change in weight from diagnosis	Ahlberg et al^70^	Diagnosis to 6 months	No difference between SLP vs control groups (−5.9 vs −6.2 kg, *P* = 0.68)
	Number malnourished	Van den Berg et al^36^	Baseline, week 10, week 30	No significant difference between groups, but more patients were malnourished in the intervention group at baseline (*P* = 0.03)

Abbreviations: BRS, Bolus Residue Scale; CRT, chemoradiotherapy; DHI, Deglutition Health Index; DIGEST, Dynamic Imaging Grade of Swallowing Toxicity; DOSS, Dysphagia Outcome and Severity Scale; EORTC, European Organisation for Research and Treatment of Cancer; FEES, Fiberoptic Endoscopic Evaluation of Swallowing; FOIS, Functional Oral Intake Scale; HADS, Hospital Anxiety and Depression Scale; MIO, maximum interincisal opening; PAS, Penetration‐Aspiration Scale; PSS‐H&N, Performance Status Scale for Head and Neck Cancer patients; QLQ‐C30, Quality of Life Questionnaire–Core 30; QLQ‐H&N35, Quality of Life Questionnaire–Head and Neck 35; RT, radiotherapy; SLP, speech‐language pathologist.

### Rationale and discussion

Dysphagia is prevalent in patients with head and neck cancer, which affects patients’ ability to achieve an adequate oral intake, and observational studies suggest this may lead to malnutrition.^66^ Speech pathologist intervention may improve or prevent decline in dysphagia. More frequent interventions by a speech pathologist may, therefore, be a strategy to improve oral intake and prevent decline in nutrition status. The current recommendation is based on this potential benefit, along with some evidence of benefit from the literature, and no known harms.

There was significant variation across the studies in the type of intervention provided, frequency of the intervention, and outcome measures reported (type and timing). In one study,^71^ the intervention was provided at least 3 months after surgery, whereas in the remaining studies, exercises were provided as a prophylactic approach during radiotherapy. It is important to note that compliance with prophylactic exercises during radiotherapy is often poor, and in two studies, the swallowing intervention was not provided by a speech pathologist. As such, many of these studies did not investigate the comprehensive and individualized approach generally used by speech pathologists in clinical practice.

There has been increasing interest in alternate service delivery models to support the provision of prophylactic exercises during radiotherapy. A study by Wall et al did not find differences in outcomes between clinician‐directed telepractice therapy and self‐directed therapy; however, patients significantly preferred the first two approaches over the patient‐directed approach.^78^

### Future research

Future research on the impact of speech pathology interventions for patients with head and neck cancer needs to address the effectiveness of specific therapy/rehabilitation strategies, as well as investigate different delivery and methodological approaches to such interventions. Such research would generally be appropriate as either an RCT or a pre‐post implementation approach, depending on the current practices of the center(s) involved. Consideration should be given to the outcome measures used, with comprehensive research ideally including all the following: patient‐reported outcomes, clinician‐rated function, and instrumental swallow assessments. Consideration should also be given to the specific patient population, time frames of the intervention, and data collection and therapy regimens (where relevant), to maximize the ability to make comparisons between studies and implement outcomes into clinical practice.

#### Question 7

In adult patients with head and neck cancer undergoing any treatment modality, does an interdisciplinary approach to nutrition management vs standard care change progression‐free survival, overall survival, nutrition intake, time to transition to full oral diet, nutrition status, weight, muscle mass, sarcopenia, myosteatosis, global quality of life, fatigue, return to work, performance status, treatment completion feeding tube dependence, treatment interruptions, treatment toxicity, surgical complications, length of stay, or unplanned hospital admission or readmission?

*Recommendation*: In adults with head and neck cancer, we recommend an interdisciplinary approach to nutrition management. An interdisciplinary approach should involve collaboration between health professionals with the expertise to manage any symptom or issue that is affecting or anticipated to affect the patient's nutrition intake or nutrition status. We recommend that the core team for nutrition management include dietitians, nurses, pharmacists, physicians, and speech pathologists. Additional members may include dental professionals, physical therapists, psychologists, and social workers.

**Certainty of Evidence:** Moderate

**Strength:** Strong

**Clinical Panel Delphi Agreement:** 100%

**External Validation Panel Delphi Agreement:** 100%

Four studies met the inclusion criteria for this question.^79^, ^80^, ^81^, ^82^ All four studies favored an interdisciplinary approach to nutrition management. Three studies reported less weight loss.^79^, ^81^, ^82^ Two reported less mucositis.^79^, ^81^ One reported a shorter hospital length of stay.^79^ Two reported decreased risk of malnutrition,^80^, ^81^ with one reporting improved nutrition status.^82^ One study reported fewer speech problems, improved handgrip strength, improved role functioning, and improved pain on a quality of life questionnaire in the intervention group but favored the control group for physical function, feeling more ill, and higher nutrition supplement use, which may be related to detection bias.^80^ This RCT was found to be low risk of bias,^80^ whereas the other studies were at “high” risk of “some concerns” for bias (Tables 18, 19, and S22–S24).^79^

**Table 18:** Summary of question 7 studies.

Study	Sample size	Population	Intervention	Comparator	Risk of bias
Kono et al^79^ Quasi‐experimental	61	Adult HNSCC patients receiving chemoradiation therapy	Quasi‐experimental postmeasure after changing to an interdisciplinary nutrition support team	Quasi‐experimental premeasurement before the nutrition support team was in place	Serious
Kristensen et al^80^	71	Survivors of HNC treated with radiation therapy with curative intent ≥5 years before recruitment through a nationwide survey	Interdisciplinary (RD, physiotherapist, psychologist, MD, SLP) residential nutrition rehabilitation program with a primary focus on the physical, psychological, and social aspects of eating problems after treatment for HNC	Waitlist for the intervention	Low
Zeng et al^81^	100	Adult patients with nasopharyngeal cancer	Patients were assessed and plans were made by an interdisciplinary team (physician, nurse, and “nutritionist”)	Standard care	Some concerns
Tang et al^82^	60	Adult patients with nasopharyngeal cancer undergoing CCRT	Patients were provided a personalized nutrition plan designed by physician, nurse, and “nutritionist”	Standard recommendations for eating during CCRT	Some concerns

Abbreviations: CCRT, concurrent chemoradiotherapy; HNC, head and neck cancer; HNSCC, head and neck squamous cell carcinoma; MD, medical doctor; RD, registered dietitian; SLP, speech‐language pathologist.

**Table 19:** Outcomes data for question 7 studies.

Topic	Outcome	Study	Timing of data collection	Results
Malnutrition/weight	% change in body weight	Kristensen et al^80^	Baseline and 3‐month follow‐up	No significant between‐group change in body weight
	Median weight loss rates	Kono et al^79^	N/A. They are comparing before and after policy change	Less weight was lost from before to after treatment in the intervention group vs the nonintervention group (3.3% vs 7.3%, *P* = 0.02)
	BMI	Kristensen et al^80^	Baseline and 3‐month follow‐up	No significant between‐group differences for BMI
	Weight	Zeng et al^81^	Admission to discharge	The intervention group had higher body weight after the intervention compared with the control group (*P* = 0.009)
	BMI	Zeng et al^81^	Admission to discharge	The intervention group had higher BMI after the intervention compared with the control group (*P* = 0.025)
	Weight	Tang et al^82^	Baseline and 3, 10, and 24 weeks	The intervention has higher weight at 3 and 24 weeks (*P* = 0.037 and 0.044, respectively)
Quality of life	EQ‐5D‐5L	Kristensen et al^80^	Baseline and 3‐month follow‐up	No between‐group differences were found on any domain
	EORTC QLQ‐C30	Kristensen et al^80^	Baseline and 3‐month follow‐up	Compared with the control, the intervention group had improved handgrip strength (*P* = 0.042), maximal mouth opening (*P* = 0.072), role functioning (*P* = 0.041), pain (*P* = 0.048), and fatigue (*P* = 0.053) but had a worse 30‐s chair stand test (*P* = 0.008)
	Diagnosis‐specific EORTC QLQ‐H&N35	Kristensen et al^80^	Baseline and 3‐month follow‐up	Compared with the control, the intervention group had greater improvements in speech problems (*P* = 0.040) but felt more ill (*P* = 0.020) and used more nutrition supplements (*P* = 0.005)
	HADS	Kristensen et al^80^	Baseline and 3‐month follow‐up	No significant between‐group differences were reported
	SF‐36	Tang et al^82^	Baseline and 3, 10, and 24 weeks	The intervention group scored higher on domains of physical functioning (*P* = 0.019), role‐physical (*P* = 0.025), bodily pain (*P* = 0.039), general health (*P* = 0.002) and vitality (*P* = 0.016), social functioning, role‐emotional (*P* = 0.165), and mental health (*P* = 0.061)
Treatment toxicities	Mucositis	Kono et al^79^	N/A. They are comparing before and after policy change	Grade 3 mucositis was higher in the nonintervention group than in the intervention group (70% vs 25%, *P* = 0.006)
	Mucositis	Zeng et al^81^	Admission to discharge	The intervention group had lower grades of mucositis vs the control (*P* < 0.05)
Length of stay	Length of hospital stay from end of treatment	Kono et al^79^	Hospital discharge	The intervention group had shorter hospital length of stay compared with the nonintervention group (12 vs 18 days, *P* = 0.01)
Malnutrition/nutrition risk	NRS‐2002 PG‐SGA	Zeng et al^81^	Admission to discharge	The intervention group had improved nutrition risk scores vs controls, with lower NRS‐2002 (*P* = 0.006) and PG‐SGA scores (*P* < 0.001)
	NRS‐2002	Tang et al^82^	Baseline and 3, 10, and 24 weeks	The intervention has lower NRS‐2002 scores at 3, 10, and 24 weeks (*P* = 0.014, 0.007, and 0.023, respectively)

Abbreviations: BMI, body mass index; EORTC, European Organisation for Research and Treatment of Cancer; EQ‐5D‐5L, EuroQol 5‐Dimension 5‐Level questionnaire; HADS, Hospital Anxiety and Depression Scale; N/A, not applicable; NRS‐2002, Nutritional Risk Screening 2002; PG‐SGA, Patient‐Generated Subjective Global Assessment; QLQ‐C30, Quality of Life Questionnaire–Core 30; QLQ‐H&N35, Quality of Life Questionnaire–Head and Neck 35; SF‐36, Short Form 36 Health Survey.

### Rationale and discussion

Malnutrition is common in patients with head and neck cancer.^18^ The factors contributing to malnutrition are multifactorial, including symptoms affecting nutrition intake as well as psychosocial and socioeconomic status. An interdisciplinary approach to nutrition management may, therefore, be an appropriate strategy to manage these complex factors and prevent nutrition decline. The current recommendation is based on this potential conceptual benefit, along with some evidence of benefit from the literature, especially the low‐bias RCT. Although more studies are needed, the balance between potential benefits and harms were considered, so it was given a rating of “strong.”

Dietitians, nurses, pharmacists, physicians, and speech pathologists are expected to be required for the nutrition management of all patients with head and neck cancer. Some patients may require a social worker to organize meal delivery or shopping and address financial hardship if it impacts food security. If dentition is affecting a patient's nutrition intake, a dental professional may be required. Mental health concerns, such as anxiety and depression, may affect nutrition intake and require support from a mental health worker (eg, psychologist, social worker, or psychiatrist). In patients with muscle loss, a physical therapist may be needed to maintain or improve muscle mass.

### Future research

RCTs of an interdisciplinary approach to nutrition management during treatment are required. If more than one site is planning to initiate an interdisciplinary team approach, a stepped‐wedge randomized design where implementation is staggered and compared between sites may be particularly suitable. If not, a quasi‐experimental design would be suitable. This could be a pre‐post design comparing findings before and after a policy change. Alternatively, one hospital without an interdisciplinary nutrition team could be compared with one without. Outcomes of importance for such studies include nutrition status, muscle mass, dysphagia, time to transition to a full oral diet, treatment interruptions, hospital length of stay, return to work, and survival.

#### Question 8

In adult patients with head and neck cancer receiving any treatment modality, does a pharmaceutical appetite stimulant compared with no pharmaceutical appetite stimulant change progression‐free survival, overall survival, nutrition intake, nutrition status, weight, muscle mass, sarcopenia, myeosteatosis, global quality of life, fatigue, return to work, performance status, treatment completion treatment interruptions, treatment toxicities, surgical complications, length of stay, or unplanned hospital admission or readmission?

*Recommendation*: In adults with head and neck cancer who are experiencing anorexia and receiving any treatment modality, we suggest dietary counseling (including oral nutrition supplements or enteral feeding) and management of other symptoms that are affecting oral intake as first‐line strategies to address anorexia and improve nutrition intake. Otherwise, a pharmaceutical appetite stimulant may be considered for short‐term use where clinically appropriate. In conjunction with the medical team and dietitian, this decision should ideally include discussion with a pharmacist specializing in oncology.

**Certainty of Evidence:** Very low/expert opinion

**Strength:** Weak

**Clinical Panel Delphi Agreement:** 100%

**External Validation Panel Delphi Agreement:** 100%

One quasi‐experimental study was found that addressed this question (Tables 20, 21, S25, and S26).

**Table 20:** Summary of question 8 studies.

Study	Sample size	Population	Intervention	Comparator	Risk of bias
Lin et al^83^ Quasi‐experimental	104	Adult patients with pharyngolaryngeal squamous cell carcinoma receiving CCRT	Patients received prophylactic megestrol acetate (400 mg/day)	Patients received CCRT alone with reactive megestrol only if <5% weight loss occurs	Serious

Abbreviation: CCRT, concurrent chemoradiotherapy.

**Table 21:** Outcomes data for question 8 studies.

Topic	Outcome	Study	Timing of data collection	Results
Weight	Weight loss	Lin et al^83^	Baseline, weekly during CCRT	The intervention group had less weight loss vs the control (*P* = 0.001)
Treatment toxicities	Mucositis Neutropenia	Lin et al^83^	Baseline, weekly during CCRT	The intervention group had shorter duration of grade 3–4 mucositis vs the control (*P* = 0.009) and shorter duration of neutropenia grade 1–2 (*P* < 0.001)
Survival	Overall survival Disease‐free survival	Lin et al^83^	Baseline, weekly during CCRT	The intervention group had significantly higher overall survival (*P* = 0.045) and directionally higher (but not significant) disease‐free survival (*P* = 0.395)

Abbreviation: CCRT, concurrent chemoradiotherapy.

### Rationale and discussion

Anorexia and cachexia occur in some patients with head and neck cancer, and observational studies indicate they are associated with adverse outcomes.^84^ In patients for whom dietary counseling and other symptom management approaches have not improved nutrition intake, a pharmaceutical appetite stimulant (eg, corticosteroids, progesterone analogs, or olanzapine) may be an appropriate strategy to increase nutrition intake and prevent nutrition decline. Pharmaceutical appetite stimulants are not recommended for long‐term use. This is because the agents that may be most beneficial, corticosteroids or progesterone analogs, may cause other, more serious, issues or toxicities with chronic use. Ghrelin receptor agonists, such as anamorelin, have been shown to improve body weight, muscle mass, and quality of life in patients with advanced lung cancer,^85^ with no studies in patients with head and neck cancer. Ghrelin receptor agonists may have side effects including gastrointestinal upset, hyperglycemia, and, rarely, fatal arrhythmia. One quasi‐experimental trial (*n* = 104) comparing megesterol acetate (400 mg/day) with no megesterol acetate unless >5% weight loss occurred met the inclusion criteria for this question.^83^ Although this study was found to be at “serious” risk for bias, it reported decreased weight loss, duration of neutropenia and Grade 3–4 mucositis, and improved overall survival in the megesterol group vs the control. The current recommendation is based on the recognition that although some potential benefit may be realized by pharmaceutical appetite stimulants, this must be balanced against the potential harmful side effects, which can be determined through discussion with an oncology clinical pharmacist.

### Future research

Adequately powered, double blind RCTs examining the effect of appetite stimulants compared with standard care are needed. Relevant outcomes include measures of appetite, nutrition intake, nutrition status, muscle mass, quality of life, and survival. Such trials should specifically recruit participants with pre‐existing anorexia and stratify by nutrition status.

#### Question 9

In adult patients with head and neck cancer receiving chemoradiation or radiation, does continuing oral intake (if tolerated) after the initiation of EN compared with not continuing oral intake change progression‐free survival, overall survival, nutrition intake, nutrition status, weight, muscle mass, sarcopenia (skeletal muscle mass + strength), myosteatosis, dysphagia, incidence of stricture, global quality of life, fatigue, return to work, performance status, treatment completion, feeding tube dependence, time of transition to a full oral diet, surgical complications, length of stay, or hospital readmission?

*Recommendation*: In adults with head and neck cancer who have commenced EN and who can safely continue oral intake per consult with a speech pathologist, we suggest that continuing any degree of oral intake may be beneficial for maintaining swallow function. The amount, type, and texture of the oral intake will be dependent on swallow safety and treatment toxicities. The volume and timing of enteral feeding should be adjusted according to what is consumed orally to optimize the opportunity for the patient to continue oral intake while also ensuring that nutrition requirements are met.

**Certainty of Evidence:** Very low/expert opinion

**Strength:** Weak

**Clinical Panel Delphi Agreement:** 100%

**External Validation Panel Delphi Agreement:** 100%

No studies met the inclusion criteria for this question.

### Rationale and discussion

Observational studies demonstrate that swallow function may deteriorate in the absence of any oral intake,^86^ of which exclusive EN during treatment may be one contributing factor. Reduced swallow function leads to difficulties in resuming oral intake.^86^ Maintaining some degree of oral intake throughout enteral feeding may, therefore, be an appropriate strategy for maintaining swallow function, which has the potential to improve quality of life. The current recommendation is based on this potential benefit and no known harms, assuming this occurs in consultation with a speech‐language therapist to minimize the risk of aspiration or other dysphagia‐related complications. This recommendation is consistent with findings from a large retrospective observational study, where maintenance of oral intake during treatment was independently associated with better long‐term diet after treatment and shorter duration of gastrostomy dependence, when adjusted for tumor and treatment burden.^86^

It is important to note that this may not be appropriate for all patients. Continuing oral intake throughout enteral feeding in some patients may be very challenging and possibly inappropriate because of the severity of dysphagia and/or other treatment‐related side effects. Decisions to maintain oral intake should, therefore, be made on an individualized basis while considering the potential for a negative psychological impact from being asked to perform a task they are unable to complete.

### Future research

Traditional RCTs to investigate maintaining any degree of oral intake alongside enteral feeding would be unethical. This is because patients who are otherwise capable of eating and drinking (albeit in small volumes) would be required to remain on nothing by mouth status if randomized to the control group. Instead, quasi‐experimental studies investigating the pre‐post effect of implementing a protocol to guide clinical decisions regarding maintaining oral intake are required. Such protocols should prompt clinicians to consider the appropriateness of maintaining oral intake based on pre‐existing dysphagia and toxicities such as pain, taste, and appetite. A stepped‐wedge randomized trial comparing the intervention with usual care may be an appropriate study design if more than one hospital is initiating a new protocol for oral intake alongside enteral feeding. Regardless of design, outcomes such as swallow function, consistency, amount of oral intake, ongoing need for EN, and dysphagia‐related complications should be assessed.

#### Question 10

In adult patients with head and neck cancer receiving any treatment modality, does use of special‐purpose nutrients (eg, arginine or glutamine) compared with not using special‐purpose nutrients change progression‐free survival, overall survival, nutrition intake, nutrition status, weight, muscle mass, sarcopenia, myosteatosis, global quality of life, fatigue, return to work, performance status, treatment completion treatment interruptions, treatment toxicities, surgical complications, length of stay, or unplanned hospital admission or readmission?

*Recommendation (arginine)*: Given the limited evidence on progression‐free and overall survival and some evidence of benefit for decreased fistula development and length of stay in adults with head and neck cancer, we suggest that using arginine‐supplemented nutrition may be acceptable at the discretion of the interdisciplinary team.

**Certainty of Evidence:** Low

**Strength:** Weak

**Clinical Panel Delphi Agreement:** 100%

**External Validation Panel Delphi Agreement:** 100%

*Recommendation (glutamine)*: Oral/enteral glutamine has been shown to reduce the severity of oral mucositis, with the potential to reduce other treatment toxicities and hospitalization, and improve treatment completion. We therefore suggest that the use of oral/enteral glutamine in patients with head and neck cancer may be acceptable at the discretion of the interdisciplinary team. Intravenous glutamine is more controversial because of one small study that reported increased mortality in the patients receiving intravenous glutamine and recent preclinical trials suggesting mechanisms through which glutamine may contribute to tumor growth and treatment resistance. For this reason, we suggest not adding parenteral glutamine to standard nutrition therapy in patients with head and neck cancer until further research becomes available to confirm its safety.

**Certainty of Evidence:** High

**Strength:** Weak

**Clinical Panel Delphi Agreement:** 100%

**External Validation Panel Delphi Agreement:** 100%

*Recommendation (ω‐3s)*: Given the inconsistent evidence for benefit but no evidence of significant harms in patients with head and neck cancer, we suggest that ω‐3–supplemented nutrition is unlikely to be harmful and may be used or not at the discretion of the interdisciplinary team.

**Certainty of Evidence:** Low

**Strength:** Weak

**Clinical Panel Delphi Agreement:** 100%

**External Validation Panel Delphi Agreement:** 100%

*Recommendation (combined nutrients)*: In patients with head and neck cancer, given the inconsistent evidence for benefit but no evidence of significant harms, we suggest that the use of combined special‐purpose nutrient or immunonutrition‐supplemented formulas is unlikely to be harmful and may be used or not at the discretion of the interdisciplinary team.

**Certainty of Evidence:** Very low

**Strength:** Weak

**Clinical Panel Delphi Agreement:** 100%

**External Validation Panel Delphi Agreement:** 100%

### Arginine

**Figure 6:**
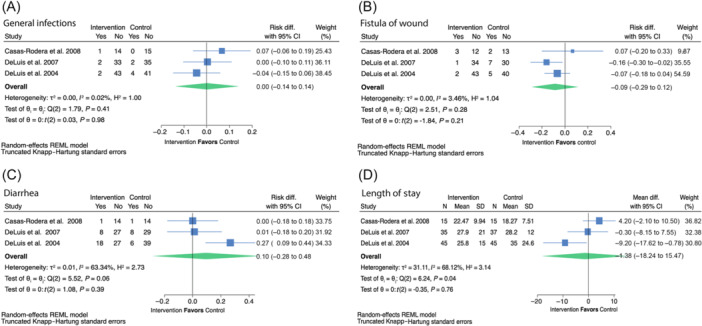
Forest plots for question 10—arginine outcomes. Forest plot (A) shows results for the effects of arginine on general infections. Forest plot (B) shows results for its effect on fistulas of wound. Forest plot (C) shows results for its effect on diarrhea. Forest plot (D) shows results for its effect on length of hospital stay. diff., difference; REML, restricted maximum likelihood.

Seven studies that met the inclusion criteria for this question examined the use of arginine.^87^, ^88^, ^89^, ^90^, ^91^, ^92^, ^93^ Six studies demonstrated a significant benefit from arginine supplementation compared with the control.^87^, ^88^, ^89^, ^90^, ^91^, ^93^ Three studies reported lower development of fistulas,^88^, ^89^, ^90^ two studies reported shorter length of stay,^89^, ^90^, ^91^ and one study reported improved disease‐free and overall survival^87^ in the intervention group. One study reported better BMI retention, improved quality of life, and lower pain and oral toxicity scores in the intervention group relative to the control.^93^ The remaining two studies found no significant difference between the two groups for any outcome (Tables 22, 23, S27, and S28). Five studies were rated as having some concerns for risk of bias and two were rated at low risk of bias. Three studies were meta‐analyzable (Figure 6) and found no significant differences in general infections (RD = 0.00, 95% CI = −0.14 to 0.14; *P* = 0.41), fistula of wound (RD = −0.09, 95% CI = −0.29 to 0.12; *P* = 0.28), diarrhea (RD = 0.10, 95% CI = −0.28 to 0.48; *P* = 0.39), and length of stay (RD = −1.38, 95% CI = −18.24 to 15.47; *P* = 0.76).

**Table 22:** Summary of question 10 studies.

Study	Sample size	Population	Intervention	Comparator	Risk of bias
**Arginine**					
Buijs et al^87^	32	Severely malnourished adult patients with HNC	Received arginine‐supplemented perioperative EN	Received standard perioperative EN	Low
De Luis et al^88^	47	Adult patients with oral and laryngeal cancer	Received enteral diet supplemented with arginine and fiber within 24 h of surgery	Received an isocaloric, isonitrogenous control formula	Some concerns
De Luis et al^89^	90	Adult patients with oral and laryngeal cancer	Received enteral diet supplemented with arginine within 24 h of surgery	Received an isocaloric, isonitrogenous control formula	Some concerns
De Luis et al^90^	72	Adult patients with oral and laryngeal cancer	EN supplemented with arginine	An isocaloric, isonitrogenous EN with no arginine	Some concerns
De Luis et al^91^	72	Adult patients with oral and laryngeal cancer	Received enteral diet supplemented with arginine and fiber within 24 h of surgery	Received an isocaloric, isonitrogenous control formula	Some concerns
Hassanein et al^93^	69	Adult patients with HNC and radiation‐induced mucositis	One intervention arm received oral glutamine and the other received arginine	No glutamine or arginine	Low
VanBokhorst‐de van der Schueren et al^92^	49	Adult patients who were severely malnourished, had HNC, and were eligible for surgery	Preoperatively received enteral formula, in which 41% of the casein was replaced with arginine, for 7–10 days	Standard‐care formula 7–10 days preoperatively that was isonitrogenous and isoenergetic	Some concerns
**Glutamine**					
Azman et al^94^	44	Adult patients scheduled for surgery, with head and neck malignancy	Glutamine supplementation (0.3 g/kg/day) One sachet three times daily (Glutamine Plus; Fresenius Kabi)	Control group did not receive the formula	Some concerns
Cerchietti et al^95^	32	Adult patients with squamous HNC, clinically unresectable tumor, committed to a treatment of induction chemotherapy plus CRT	Received L‐alanyl‐L‐glutamine (0.4 g per kilogram of body weight per day)	Received a saline placebo	Some concerns
Chattopadhyay et al^96^	70	Adult patients with malignant neoplasms of the head and neck region, assigned to receive radical RT, and with performance status not worse than 50%	Received 10 g oral glutamine suspension daily 2 h before radiation	Nothing before radiation	High
Diwan and Khan^97^	60	Adult patients with HNSCC receiving either definitive or adjuvant radiation therapy	Received 10 g glutamine powder daily within 1 h before radiation and again 7–8 h after radiation	Received a placebo and followed the same regimen	Some concerns
Fatima et al^98^	60	Adult patients with HNC assigned to receive definitive or adjuvant RT and CRT	Oral glutamine (10 g) once daily	Placebo	Some concerns
Hassanein et al^93^	69	Adult patients with HNC and radiation‐induced mucositis	One intervention arm received oral glutamine and the other received arginine	No glutamine or arginine	Low
Huang et al^99^	71	Adult patients with cancer of nasopharynx, oropharynx, hypopharynx, larynx, or oral cavity	Oral glutamine (5 g glutamine and 10 g maltodextrin) three times per day	Placebo	Low
Pachón et al^129^ Quasi‐experimental	220	Adult patients with HNSCC, without mucositis, and with no other prophylactic measures	A post–policy change group given glutamine (10 g) every 8 h	A pre–policy change group in which patients do not receive glutamine	Serious
Ibrahim et al^100^	50	Adult patients with HNC receiving RT with mucositis in the second to third week of RT	Oral glutamine (5 g) three times daily 30 min before a meal	Placebo	High
Khanum et al^101^	40	Adult patients with HNSCC undergoing conventional RT with concurrent chemotherapy either radical or post‐op	10 g of glutamine in 1000 ml of water 2 h before RT on all days of irradiation	Standard care	Some concerns
Lopez‐Vaquero et al^102^	50	Adults with primary cancer in any head and neck location undergoing RT	10 g of L‐glutamine three times per day with meals	Maltodextrin placebo	Some concerns
Pathak et al^103^	60	Adult patients with locally advanced carcinoma of the oropharynx and larynx (stage III–IV) receiving CCRT	10 mg oral glutamine 2 h before RT 5 days per week over 7 weeks of RT	Standard care	Low
Pattanayak et al^104^	162	Adult patients with HNSCC who were treated at the Department of Radiation Oncology	Patients told to swish the oral glutamine (15 g) in a glass of water for 2 min and then swallow. This was done twice per day throughout treatment	Control did not do this	Low
Tsujimoto et al^105^	40	Adult patients with squamous cell carcinoma of the nasopharynx, oropharynx, hypopharynx or larynx who were scheduled to undergo CRT	Oral glutamine 10 g three times a day	Placebo	Low
Tsujimoto et al^106^	38	Adult patients with HNSCC receiving RT	Oral glutamine 10 g three times a day	Placebo	Low
**n‐3 Enhanced**					
Cereda et al^107^	159	Adult patients with newly diagnosed HNC	An energy‐dense, high‐protein, n‐3–enriched oral formula (Resource Support Plus; Nestlé Health Science)	Control group did not receive the formula	Low
De Luis et al^108^	73	Adult ambulatory postsurgical patients with oral and laryngeal cancer without a recent weight loss (<5% at 3 months before the study)	Received an n‐3–enhanced supplement that was higher in EPA	Received an arginine‐enhanced supplement	Some concerns
Hanai et al^109^	27	Adult patients undergoing HNC surgery who required resection and free flap reconstruction and who exhibited ≥5% weight loss.	Two packs of an EPA‐enriched oral nutrition supplement per day (ProSure; Abbott Laboratories Ltd).	Standard care	Some concerns
Jantharapattana and Orapipatpong, 2020^110^	62	Adult patients with HNC eligible for curative surgical treatment, with a score of ≥2 on the MST	Received an EPA‐containing nutrition supplement (ProSure) 7 days presurgery and then 14 days post‐op	Received an isocaloric conventional supplement (Blendera; Thai Otsuka Pharmaceutical Co Ltd) 7 days presurgery and then 14 days post‐op	Some concerns
Pottel et al^111^	85	Adult patients with HNSCC with or without systemic treatment	Patients received 7.5 ml twice a day (15 ml in total) of echium oil (BioMega SDAW, containing 235 ± 30 mg/ml ALA + 95 ± 13 mg/ml ALA SDA + 79 ± 10 mg/ml GLA) daily	Patients received same amount of n‐3–deficient sunflower oil	Low
Solís‐Martínez et al^112^	64	Adult patients with HNSCC about to start any antineoplastic treatment	Two bottles of polymeric high‐protein supplement enriched with 2 g EPA per day	Two bottles of a standard polymeric supplement along with 24 g of calcium caseinate per day (isocaloric and isonitrogenous)	Some concerns
**Combined immunonutrition**					
Ascoli et al^113^ Quasi experimental	199	Adult patients with HNC receiving major ablative therapy	Received oral immunomodulating formula (Oral Impact; Nestlé) enriched with l‐arginine, n‐3, and RNAs	No immunomodulating formula	Serious
Boisselier et al^114^	180	Adult patients with HNSCC treated surgically with curative intent and eligible for adjuvant CRT to begin >8 weeks after surgery	Oral immunomodulating formula (Oral Impact) enriched with l‐arginine, n‐3, and RNAs	The same product but nonenriched with immunonutrients. Isocaloric and isonitrogenous	Low
Casas‐Rodera et al^131^	44	Adult patients with oral and laryngeal cancer	Two intervention groups: one receiving EN arginine and one receiving EN arginine, RNA, and n‐3	Standard polymeric EN	Some concerns
Chitapanarux et al^130^	40	Adult patients with HNC undergoing CCRT	Regular diet plus 250 kcal of a formula of corn oil, medium‐chain triglyceride, and fish oil and protein (casein, arginine, and glutamine) given 1 h before and after RT session	Regular diet	High
Chitapanarux et al^115^	40	Adult patients with HNC	Regular diet plus 250 kcal of a formula of corn oil, medium‐chain triglyceride, and fish oil and protein (casein, arginine, and glutamine) given 1 h before and after RT session	Regular diet	High
Dechaphunkul et al^116^	110	Adult patients with HNC including 3‐week cisplatin cycles	Patients received immunonutrition (n‐3, arginine, dietary nucleotides, and soluble fiber) 5 days before each chemotherapy session	Patients received an isocaloric, isonitrogenous control formula on the same schedule	Low
Falewee et al^117^	205	Adult patients with oropharyngeal and pharyngolaryngeal tumor with anticipated surgery	Impact with immune nutrients (arginine, RNA, n‐3, EPA, DHA); one group received this preoperatively and a second intervention group received it perioperatively	Perioperative Impact (Nestlé) without immune nutrients	Low
Felekis et al^118^	40	Adult patients with HNSCC	EN enriched with the nutrients arginine, RNA, and n‐3 (Impact; Nestlé)	No preoperative nutrition support	Some concerns
Fietkau et al^119^	111	Adult patients inoperable head and neck or esophageal cancer (only 8%), intended for CCRT	Patients received 500 ml Supportan (Fresenius Kabi), which contains high amounts of fat (40% of energy), protein (27% of energy), and n‐3 from fish oil (2.0 g EPA and 0.85 g DHA) and is low in carbohydrate (33% of energy)	Patients received 500 ml of the enteral standard nutrition Fresubin Energy Fibre (Fresenius Kabi) (protein, 15% of energy; carbohydrate, 50% of energy)	Some concerns
Ghosh et al^120^	60	Adult patients with advanced squamous cell carcinoma of the oral cavity, oropharynx, larynx, or hypopharynx	Received immunonutrition (arginine, RNA, n‐3, EPA, DHA via Impact)	Received an isocaloric, isonitrogenous control feed manufactured for the trial	Some concerns
Imai et al^121^	40	Adult patients with HNC, treated with CCRT involving cisplatin	Patients received beta‐hydroxy‐beta‐methylbutyrate/arginine/glutamine supplement	Standard care	Some concerns
Jantharapattana and Orapipatpong, 2020^110^	62	Adult patients with HNC eligible for curative surgical treatment, with a score of ≥2 on the MST	Received an EPA‐containing nutrition supplement (ProSure) 7 days presurgery and then 14 days post‐op	Received an isocaloric conventional supplement (Blendera) 7 days presurgery and then 14 days post‐op	Some concerns
Kotb et al^122^	176	Adult patients with oral cavity or mandibular tumors undergoing resection	Patients were given oral n‐3 and IV L‐alanyl‐L‐glutamine	Standard care	Some concerns
Kuroki et al^123^	75	Adult patients with HNSCC receiving platinum‐based CRT	Patients received an immunonutrition mixture of beta‐hydroxy‐beta‐methylbutyrate, arginine, and glutamine	Patients received no immunonutrition	High
Muangwong et al^124^	87	Adults patients with locally advanced HNC	Patients received an immunonutrition supplement packet of arginine, glutamine, and fish oil	No immunonutrition	High
Mueller et al^125^ Quasi‐experimental	96	Adult patients undergoing salvage surgery for recurrent HNSCC	After policy change, patients received Oral Impact (three units per day for 5 days) before surgery, enriched with n‐3 (1.0 g/unit), arginine (3.8 g/unit), RNA‐nucleotides (0.39 g/unit), and soluble guar fiber (3 g/unit)	A pre–policy change group in which patients did not receive immunonutrition	Serious
Sittitrai et al^126^	116	Adult patients with HNC classified as ECOG performance status 0–1, undergoing major clean‐contaminated surgery	Blenderized perioperative immune‐enhancing diet containing arginine, glutamine, and fish oil (Neo‐Mune; Thai Otsuka Pharmaceutical Co Ltd) for 7 days preoperatively and 14 days post‐op	Blenderized chicken, egg, rice bran oil, and sucrose	Some concerns
Turnock et al^127^	8	Patients with HNC scheduled for radical resection of the oral cavity, pharynx or larynx	Patients received three 74‐g packets per day of powdered Oral Impact (arginine, n‐3, DHA, EPA, RNA) to be taken for 5 days immediately preceding day of surgery	Standard‐care group receiving Isosource Standard (Nestlé Health Science)	Low
Yeh et al^128^	68	Adult patients with HNC and an ECOG performance score measured at 3 months	Ethanwell (a protein‐ and energy‐dense oral nutrition supplement that contains several ingredients, including n‐3, glutamine, selenium, and CoQ10; Ethan Nutraceutical Co Ltd) and Ethanzyme (an enzyme product composed of multiple probiotics and vitamins; Ethan Nutraceutical Co Ltd)	Isocal (Nestlé)	Some concerns

Abbreviations: ALA, alpha‐linolenic acid; CCRT, concurrent chemoradiotherapy; CoQ10, coenzyme Q10; CRT, chemoradiotherapy; DHA, docosahexaenoic acid; ECOG, Eastern Cooperative Oncology Group; EN, enteral nutrition; EPA, eicosapentaenoic acid; GLA, gamma‐linolenic acid; HNC, head and neck cancer; HNSCC, head and neck squamous cell carcinoma; IV, intravenous; MST, Malnutrition Screening Tool; n‐3, ω‐3 fatty acid; post‐op, postoperatively; RT, radiotherapy.

**Table 23:** Outcomes data for question 10 studies.

Topic	Outcome	Study	Timing of data collection	Results
**Arginine**				
Complications/adverse treatment effects	General infections	De Luis et al^90^	Baseline and day 12	There was no difference in infections of any kind (5.7% vs 5.4, *P* = NS)
	General infection	De Luis et al^89^	Baseline to day 14	No significant difference between intervention vs control (4% vs 9%, *P* = NS)
	Infections	De Luis et al^91^	Presurgery to POD 10	No difference between intervention vs control (23.6% vs 20.6%, *P* = NS)
	Infection of wound	De Luis et al^90^	Baseline to day 12	No difference between intervention and control regarding wound infections (0% vs 0%, *P* = NS)
	Infection of wound	De Luis et al^89^	Baseline to day 14	No infection of wound occurred in either group
	Wound infection	De Luis et al^91^	Presurgery to POD 10	No difference between intervention vs control (2.63% vs 2.90%, *P* = NS)
	Wound infection	De Luis et al^88^	Baseline to day 14	The intervention group had lower incidence of wound infection vs the control, but this did not achieve significance (4.3% vs 12.5%, *P* = NS)
	Fistula of wound	De Luis et al^90^	Baseline and day 12	The intervention group had significantly lower percent of participants with fistula compared with the control group (2.8 vs 18.9, *P* < 0.05)
	Fistula of wound	De Luis et al^89^	Baseline to day 14	The intervention group had lower incidence of fistula of wound vs the control (5% vs 11%, *P* < 0.05)
	Fistula	De Luis et al^91^	Presurgery to POD 10	Fistula development was lower in the intervention group vs the control (5.2% vs 17.6%, *P* = 0.026)
	Fistula	De Luis et al^88^	Baseline to day 14	The intervention group had reduced incidence of fistula vs the control (0% vs 20.8%, *P* < 0.05)
	Diarrhea	De Luis et al^91^	Presurgery to POD 10	No difference between intervention vs control (7.89% vs 5.88%, *P* = NS)
	GI problems/diarrhea	De Luis et al^90^	Baseline and day 12	No difference between intervention and control regarding diarrhea (22.8 vs 21.6, *P* = NS)
	Diarrhea	De Luis et al^89^	Baseline to day 14	The intervention group had more diarrhea vs the control (40% vs 13%, *P* < 0.05)
	Diarrhea	De Luis et al^88^	Baseline to day 14	Intervention group had more diarrhea vs control, but this did not achieve significance (17.4% vs 8.3%, *P* = NS)
	Post‐op complications	VanBokhorst‐de van der Schueren et al^92^	7–10 days pre‐op to 7 days post‐op	No difference in post‐op complications between intervention and control group (47% vs 59%, *P* = 0.723)
	Post‐op complications	De Luis et al^88^	Baseline to day 14	No differences for post‐op complications between intervention vs control (21.7% vs 16.7%, *P* = NS)
	WHO oral toxicity scale	Hassanein et al^93^	Baseline and second, fifth, and seventh week of RT	The arginine group had improved WHO scores vs the control starting by week 5 (*P* < 0.001). No difference between arginine and glutamine group
	Pain VAS score	Hassanein et al^93^	Baseline, second, fifth, and seventh week of RT	The arginine group had improved pain VAS scores vs the control starting by week 5 (*P* < 0.0037) and persisting at week 7 (*P* = 0.0001). No difference between arginine and glutamine group
Length of stay	Hospital length of stay	De Luis et al^90^	Baseline and day 12	No difference between intervention and control regarding hospital length of stay (27.9 vs 28.2 days, *P* = NS)
	Length of stay	De Luis et al^89^	Baseline to day 14	The intervention group had decreased length of stay vs the control (25.8 vs 35 days, *P* < 0.05)
	Length of stay	De Luis et al^91^	Presurgery to POD 10	Post‐op length of stay was less in the intervention group vs the control (24.3 vs 36.1 days, *P* = 0.036)
	Length of stay	De Luis et al^88^	Baseline to day 14	The intervention group had decreased length of stay vs the control but did not achieve significance (22.8 vs 31.2 days, *P* = 0.07)
Weight/body composition	Post‐op weight loss	De Luis et al^90^	Baseline and day 12	No difference between intervention and control regarding post‐op weight loss (2.3% vs 1.8%, *P* = NS)
	BMI	Hassanein et al^93^	Baseline and second, fifth, and seventh week of RT	The arginine group retained their BMI better than the control (*P* = 0.001). No difference between arginine and glutamine group
Survival	Mortality	De Luis et al^88^	Baseline to day 14	No difference in mortality between the intervention vs the control group (13% vs 8.3%, *P* = NS)
	Disease‐free survival	Buijs et al^87^	Baseline to 34.8 months	Being assigned to the intervention group was associated with improved disease‐free survival (HR, 4.167; 95% CI, 1.389–12.500)
	Overall long‐term survival	Buijs et al^87^	Baseline to 34.8 months	Being assigned to the intervention group was associated with improved overall survival (HR, 2.632; 95% CI, 1.142–6.061)
QOL	OHIP‐14	Hassanein et al^93^	Baseline and second, fifth, and seventh week of RT	The arginine group had OHIP‐14 scores vs the control starting by week 5 (*P* = 0.001). No difference between arginine and glutamine group
**Glutamine**				
Complications/adverse treatment effects	Oral mucositis	Huang et al^99^	1 week before RT, during RT, and for 2 weeks after completion of RT	No significant difference between groups for oral mucositis but directionally favored the glutamine group (OR, 0.3; 95% CI, 0.05–1.67; *P* = 0.169)
	Oral mucositis	Pathak et al^103^	Baseline to week 7	Incidence of mucositis was less severe in the intervention group vs the control (grade 3 oral mucositis 42.86% vs 96.43%, *P* < 0.001)
	Mucositis	Tsujimoto et al^105^	Baseline and weekly for 6 weeks	There was no difference in incidence of mucositis, but mean maximal grade of mucositis was lower in the intervention group (*P* = 0.005)
	Mucositis	Lopez‐Vaquero et al^102^	Baseline and week 6	No difference in incidence of mucositis between intervention and control (76% vs 87.5%, *P* = 0.324) or functional mucositis (76% vs 75%, *P* = 0.511). Grade did not differ between groups for either outcome
	Mucositis	Chattopadhyay et al^96^	Baseline and then weekly until end of RT	Fewer patients in the intervention vs the control developed grade 3 mucositis (14.29% vs 37.14%, *P* = 0.02) and grade 4 mucositis (2.86% vs 17.14%, *P* = 0.04). Time of onset was also greater (16.5 vs 7.1 days, *P* < 0.001), and duration was shorter (6.6 vs 9.2 days, *P* < 0.001)
	Mucositis	Pachón et al^129^	Pre‐RT to post‐RT	Mucositis was lower in the glutamine vs no‐glutamine group but NS overall (50.4% vs 59.5%, *P* = 0.14)
	Mucositis by site Oral cavity Oropharynx Supraglottis Glottis	Pachón et al^129^	Pre‐RT to post‐RT	Mucositis was lower in the glutamine vs nonglutamine group with varying significance. Oral cavity mucositis: 80% vs 81.8%, *P* = 0.87; oropharynx mucositis: 53.3% vs 77.1%, *P* = 0.04; supraglottis mucositis: 27.3% vs 43.5%, *P* = 0.21; and glottis mucositis: 7.1% vs 30.4%, *P* = 0.1
	Mucositis onset	Pattanayak et al^104^	Beginning to end of radiation therapy	For the intervention group, the first case of mucositis began in weeks 2–3, with most not beginning until weeks 4–6. In the control group, mucositis began in week 1, with most occurring between weeks 2 and 4 (*P <* 0.05)
	Mucositis onset	Fatima et al^98^	Weekly during RT (6–7 weeks)	The intervention group took longer to acquire mucositis compared with the control (*P* < 0.001)
	Grade ≥3 mucositis	Khanum et al^101^	Baseline to week 6	The intervention group had lower incidence of grade ≥3 mucositis compared with the control (22% vs 55%, *P* = 0.006). This resolved completely 2 weeks post‐RT in the intervention but not the control group (0% vs 30%, *P* = 0.008).
	Mucositis severity	Fatima et al^98^	Weekly during RT (6–7 weeks)	The intervention vs the control group had fewer patients develop grade 3 (*P* = 0.040) and grade 4 (*P* = 0.004) mucositis
	WHO Oral Toxicity Scale	Hassanein et al^93^	Baseline and second, fifth, and seventh week of RT	The glutamine group had improved WHO scores vs the control starting by week 5 (*P* < 0.001). No difference between arginine and glutamine groups
	WHO Oral Toxicity Scale	Ibrahim et al^100^	Baseline, 2 weeks, end of RT	The intervention group has lower WHO scores at both 2 weeks (*P* = 0.004) and end of RT (*P* < 0.001) vs the control
	Intensity of objective mucositis developed	Cerchietti et al^95^	Unclear but presumably from baseline to end of chemotherapy	The intervention group had lower intensity of objective mucositis vs the control (0.82 vs 1.33, *P* = 0.044)
	Percentage of patients with severe objective mucositis	Cerchietti et al^95^	Unclear but presumably from baseline to end of chemotherapy	The intervention group had lower percent of severe mucositis vs the control (14% vs 67%, *P* = 0.007)
	Patients with mucositis WHO grade 4	Cerchietti et al^95^	Unclear but presumably from baseline to end of chemotherapy	The intervention group had lower percentage with grade 4 mucositis vs the control (0% vs 33%, *P* = 0.042)
	Mucositis severity grades 2 and 3	Pattanayak et al^104^	Beginning to end of radiation therapy	For the intervention group, grade 2 did not appear until weeks 4–7, and no one had grade 3. For the control, grade 2 began in weeks 1–7. Grade 3 began in weeks 2–7 and represented *P* < 0.05
	Mean days until mucositis	Diwan and Khan^97^	Start of radiation therapy to 9 weeks	Although all patients developed mucositis in both groups, the intervention group took longer to develop mucositis vs the control (32.86 vs 17.06 days, no *P* value calculated)
	Mucositis severity by grade	Diwan and Khan^97^	Start of radiation therapy to 9 weeks	Shown graphically and without significance testing, number of patients with grade 2 and grade 3 visibly lower in the intervention group vs control
	Hospital admission due to severe mucositis	Cerchietti et al^95^	Unclear but presumably from baseline to end of chemotherapy	No difference was found in intervention vs control (7% vs 33%, *P* = NS)
	Hospitalization for treatment toxicities	Pathak et al^103^	Baseline to week 7	Hospitalization for treatment toxicities was less in the intervention group vs the control (23.33% vs 53.33%, *P* = 0.03)
	Treatment completion	Pattanayak et al^104^	Beginning to end of radiation therapy	Fifty‐seven percent in the intervention group completed six cycles of chemotherapy, and 95% completed radiation in the 7‐week period. No one in the control tolerated 6 weeks of chemotherapy, and 24% required radiation extended into an eighth week. No *P* value given
	Treatment interruptions	Pathak et al^103^	Baseline to week 7	Treatment interruptions were fewer in the intervention group vs the control (16.67% vs 46.67%, *P* = 0.025)
	Pain	Tsujimoto et al^105^	Baseline and weekly for 6 weeks	Pain was significantly reduced in the intervention vs the control in weeks 5 and 6 (*P* < 0.05)
	Pain	Lopez‐Vaquero et al^102^	Baseline and week 6	No difference between intervention and control for pain (2.32 vs 1.96 visual analog scale units, *P* = 0.574)
	Pain	Pattanayak et al^104^	Beginning to end of radiation therapy	In the intervention vs the control, fewer people experienced pain level 4–5 (30.8% vs 98.7%, *P* < 0.001) and level 5–6 (67.9% vs 100%, *P* < 0.001)
	Pain VAS score	Hassanein et al^93^	Baseline and second, fifth, and seventh week of RT	The glutamine group had improved pain VAS scores vs the control starting by week 5 (*P* < 0.0037). No difference between arginine and glutamine group
	Pain VAS score	Ibrahim et al^100^	Baseline, 2 weeks, end of RT	The intervention group has lower pain scores at both 2 weeks and end of RT vs the control (*P* < 0.001)
	Pain intensity	Cerchietti et al^95^	Unclear but presumably from baseline to end of chemotherapy	Pain intensity was significantly lower in the intervention vs the control group (*P* = 0.008)
	Pain severity using WHO stepladder	Diwan and Khan^97^	Start of radiation therapy to 9 weeks	Pain severity qualitatively less in the intervention vs control
	Dermatitis	Lopez‐Vaquero et al^102^	Baseline and week 6	Incidence of dermatitis was significantly lower in the intervention vs the control (84% vs 100%, *P* = 0.038) as was dermatitis severity (*P* = 0.032).
	Nausea	Pattanayak et al^104^	Beginning to end of radiation therapy	In the intervention vs the control, fewer people experienced nausea level 4–5 (2.4% vs 19.75%, *P* < 0.001) and level 5–6 (6.1% vs 40.7%, *P* < 0.001)
	Edema	Pattanayak et al^104^	Beginning to end of radiation therapy	In the intervention vs the control, fewer people experienced edema level 4–5 (1.2% vs 19.75%, *P* < 0.001) and level 5–6 (6.2% vs 29.6%, *P* < 0.001)
	Cough	Pattanayak et al^104^	Beginning to end of radiation therapy	In the intervention vs the control, fewer people experienced cough level 4–5 (11.1% vs 44.6%, *P* < 0.001) and level 5–6 (29.6% vs 58.02%, *P* < 0.001)
Dysphagia	Dysphagia	Pattanayak et al^104^	Beginning to end of radiation therapy	In the intervention vs the control, fewer people experienced dysphagia level 4–5 (19.7% vs 97.5%, *P* < 0.001) and level 5–6 (62.9% vs 100%, *P* < 0.001)
	Dysphagia severity	Pathak et al^103^	Baseline to week 7	Dysphagia was less severe in the intervention group vs the control (grade 3 dysphagia 39.39% vs 92.86%, *P* < 0.001)
	Odynophagia by site: Oral cavity Oropharynx Supraglottis Glottis	Pachón et al^129^	Pre‐RT to post‐RT	Odynophagia was lower in the glutamine vs nonglutamine group with varying significance. Oral cavity odynophagia: 42.9% vs 54.5%, *P* = 0.39; oropharynx odynophagia: 53.3% vs 80%, *P* = 0.02; supraglottis odynophagia: 63.6% vs 100%, *P* = 0.001; and glottis odynophagia: 71.4% vs 91.3%, *P* = 0.11
	Odynophagia grade III–IV by site: Oral cavity Oropharynx Supraglottis Glottis	Pachón et al^129^	Pre‐RT to post‐RT	Odynophagia grades III–IV were lower in the glutamine vs nonglutamine group, with three sites achieving significance. Oral cavity odynophagia grades III–IV: 8.6% vs 27.3%, *P* = 0.17; oropharynx odynophagia grades III–IV: 10% vs 40%, *P* = 0.01; supraglottis odynophagia grades III–IV: 15.2% vs 60.9%, *P* = 0.0002; and glottis odynophagia grades III–IV: 14.3% vs 43.5%, *P* = 0.1
	Use of Ryle's tube	Pathak et al^103^	Baseline to week 7	Use of Ryle's tube was required less often in the intervention group vs control (26.67% vs 56.67%, *P* = 0.03)
Nutrition intake	Energy intake	Azman et al^94^	Baseline to 4 weeks postsurgery	No difference between groups (*P* > 0.05)
QOL	QOL score	Azman et al^94^	Baseline to 4 weeks postsurgery	QOL was better overall in the intervention group vs the control (2.227 vs 8.818, *P* < 0.05). Domains of improved QOL for intervention include pain (*P* = 0.010), swallowing (*P* = 0.007), social eating (*P* = 0.046), sexuality (*P* = 0.041), and specific weight loss (*P* < 0.001)
	Pain, swallowing, social eating, sexuality, and specific weight loss domains	Azman et al^94^	Baseline to 4 weeks postsurgery	Pain, swallowing, social eating, sexuality, and specific weight loss were improved in the intervention vs control (*P* < 0.05 for each). No difference was found for speech or senses
	MDASI‐HN QOL questionnaire	Lopez‐Vaquero et al^102^	Baseline and week 6	No difference between groups on any domain
	OHIP‐14	Hassanein et al^93^	Baseline and second, fifth, and seventh week of RT	The glutamine group had OHIP‐14 scores vs the control starting by week 5 (*P* = 0.001). No difference between arginine and glutamine groups
Weight/body composition	Weight loss	Lopez‐Vaquero et al^102^	Baseline and week 6	No difference between intervention and control (−3.33 vs −2.55, *P* = 0.526)
	Weight loss	Cerchietti et al^95^	Unclear but presumably from baseline to end of chemotherapy	The intervention group lost less weight vs the control group, but this did not achieve significance (−3.3 vs −5.77 kg, *P* = NS)
	Significant weight loss (>3 kg from baseline)	Pathak et al^103^	Baseline to week 7	The percentage of patients with significant weight loss was less in the intervention vs the control (71.43% vs 100%, *P* = 0.004)
	BMI	Hassanein et al^93^	Baseline and second, fifth, and seventh week of RT	The glutamine group retained their BMI better than the control (*P* = 0.001). No difference between arginine and glutamine group
	BMI	Ibrahim et al^100^	Baseline, 2 weeks, end of RT	No differences in BMI between groups at any time point
	Fat‐free mass	Azman et al^94^	Baseline to 4 weeks postsurgery	The intervention group gained fat‐free mass, whereas the control lost fat‐free mass (3.527 vs −3.209 kg, *P* < 0.05)
Survival	Overall and progression‐free survival	Tsujimoto et al^106^	Baseline to 5 years	No differences were reported between intervention vs control for overall survival (55.2 vs 48.3 months, *P* = 0.583) or progression‐free survival (46.7 vs 43.6 months, *P* = 0.682)
**ω‐3 enriched**				
Complications/adverse events	Wound complications	Hanai et al^109^	Up to 14 days post‐op	There were fewer wound complications in the intervention vs control group, but this did not achieve significance (4 vs 7, *P* = 0.27)
	Wound complications	Jantharapattana and Orapipatpong, 2020^110^	7 days pre‐op to 4 months post‐op	No difference between intervention vs control group for wound complications (12.9% vs 22.6%, *P* = 0.50)
	Infections (pulmonary and surgical site)	Jantharapattana and Orapipatpong, 2020^110^	7 days pre‐op to 4 months post‐op	No difference between intervention vs control group for pulmonary infections (6.5% vs 0%, *P* = 0.51) or surgical site infection (12.9% vs 16.1%, *P* = 1.0)
	Adverse events	Fietkau et al^119^	Before and after RT and at 6‐ to 7‐week follow‐up	No significant difference between groups
	Treatment‐related toxicities	Pottel et al^111^	Baseline to week 7	No difference in treatment toxicities was noted between intervention and control (51.2% vs 69.0%, *P* = 0.122)
	Mucositis	Cereda et al^107^	Baseline, end of RT, 1 month, 3 months	No difference in mucositis (91% vs 96.3%, *P* = 0.21) or severe mucositis (26.9 vs 32.1%, *P* = 0.49) between intervention group and control
	Temporary interruption of RT	Cereda et al^107^	Baseline, end of RT, 1 month, 3 months	No difference between intervention group and control (41% vs 39.5%, *P* = 0.87)
	RT and/or ST reduction/complete suspension	Cereda et al^107^	Baseline, end of RT, 1 month, 3 months	There was less RT/ST reduction/complete suspension in the intervention group vs the control (9% vs 22.2%, *P* = 0.029)
	Hospitalization	Pottel et al^111^	Baseline to week 7	No difference in hospitalization was noted between intervention and control (27.9% vs 35.7%; *P* = 0.490)
Length of stay	Median days of hospitalization	Jantharapattana and Orapipatpong, 2020^110^	Baseline to day 21	No significant difference between intervention vs control group (7 vs 8 days, *P* = 0.95)
Nutrition intake	Energy intake	Pottel et al^111^	Baseline to week 7	No difference in energy intake was noted between intervention and control (no statistics provided)
	Energy intake	Jantharapattana and Orapipatpong, 2020^110^	Baseline to day 21	No significant difference reported between groups
	Energy/protein intake	Solís‐Martínez et al^112^	Baseline to week 6	No significant differences between groups for energy or protein intake, but patients’ decreased tolerance of the intervention formula was noted
	Energy intake (kcal/kg/day)	Cereda et al^107^	Baseline, end of RT, 1 month, 3 months	Energy Intake was higher in the intervention group at every time point via a mixed effect regression model (*P* < 0.001)
Nutrition status	PG‐SGA	Pottel et al^111^	Baseline to week 7	No difference in PG‐SGA was noted between intervention and control (no statistics provided)
	NRS‐2002 SGA	Fietkau et al^119^	Before and after RT and at 6‐ to 7‐week follow‐up	NRS‐2002 directionally favored the intervention group but did not achieve significance (*P* = 0.075), although the score improved for the intervention group (*P* = 0.0165) and not for the control (*P* > 0.05). For SGA, the intervention group had improved nutrition status vs control at follow‐up (*P* = 0.0065)
QOL	EORTC QLQ‐C30	Solís‐Martínez et al^112^	Baseline to week 6	No differences were found between groups on any domain except for fatigue, which was lower in the intervention vs the control group (−2 vs 6, *P* = 0.012)
	EORTC QLQ‐C30 and QLQ‐H&N35	Pottel et al^111^	Baseline to week 7	No difference in QOL was noted between intervention and control (no statistics provided)
	Global QOL	Cereda et al^107^	Baseline, end of RT, 1 month, 3 months	Global QOL was higher in the intervention group at every time point via mixed‐effects regression model (*P* < 0.001)
	Karnofsky index EORTC QLQ30	Fietkau et al^119^	Before and after RT and at 6‐ to 7‐week follow‐up	No difference between groups found for Karnofsky index or Global Health Status, but the intervention group had improved appetite vs control (25.11 vs 6.22, *P* = 0.030)
Weight/body composition	Weight loss	Pottel et al^111^	Before and after RT	No significant difference between intervention group and control (8.9% vs 7.6%, *P* = 0.303)
	Weight loss	Jantharapattana and Orapipatpong, 2020^110^	7 days pre‐op to 4 months post‐op	No difference between intervention group vs control (2.95 kg vs 2.82 kg, *P* = NS)
	Body weight and BMI	Fietkau et al^119^	Before and after RT and at 6‐ to 7‐week follow‐up	Directionally, the intervention was favored for every variable vs the control after RT and at follow‐up, but this did not achieve significance
	Change in weight/BMI	Solís‐Martínez et al^112^	Baseline to week 6	No difference in intervention vs control group for weight (−0.3 vs −2.1 kg, *P* = 0.468) or BMI (−0.1 vs −0.8, *P* = 0.195)
	Body weight	Hanai et al^109^	14 days pe‐op to 14 days post‐op	No difference between groups (*P* = 0.62)
	Body weight	Cereda et al^107^	Baseline, end of RT, 1 month, 3 months	Body weight was higher at every time point in the intervention group via a mixed‐effects regression model (*P* < 0.001)
	Lean body mass	Hanai et al^109^	14 days pe‐op to 14 days post‐op	No difference between groups (*P* = 0.33)
	Change in lean body mass	Solís‐Martínez et al^112^	Baseline to week 6	No difference in intervention vs control group (−0.2 vs −1.3 kg, *P* = 0.186)
	Percent reduction in lean body mass	Jantharapattana and Orapipatpong, 2020^110^	7 days pre‐op to 4 months post‐op	No difference between intervention group vs control (2.16% vs 1.25%, *P* = NS)
	Change in fat mass	Solís‐Martínez et al^112^	Baseline to week 6	No difference in intervention vs control group (0.2 vs −1.2 kg, *P* = 0.778)
	Change in fat mass measured through BIA	Pottel et al^111^	Before and after RT	The intervention group had greater increase in fat mass relative to the control (10.55% vs −3.90%, *P* < 0.05)
	Change in fat mass, fat‐free mass, and lean mass via DXA	Pottel et al^111^	Before and after RT	No significant differences were observed between groups (statistics not provided)
	Body cell mass, fat mass, lean mass, lean tissue mass, handgrip strength	Fietkau et al^119^	Before and after RT and at 6‐ to 7‐week follow‐up	Directionally, the intervention was favored for every variable vs the control after RT and at follow‐up, but this did not achieve significance
	Phase angle	Cereda et al^107^	Baseline, end of RT, 1 month, 3 months	No difference between groups
	Change in grip strength	Pottel et al^111^	Before and after RT	No difference in loss of grip strength was reported between intervention and control (−0.86% vs −0.78%, *P* > 0.05)
	Handgrip strength	Cereda et al^107^	Baseline, end of RT, 1 month, 3 months	Handgrip strength trended lower in the intervention group vs the control at every time point via mixed‐effects regression model (*P* = 0.057)
**Combined immunonutrition**				
Complications/adverse events	Mucositis and other toxicities	Boisselier et al^114^	1 month after CRT	No difference between intervention vs control groups in any toxicity (grade 3–4 mucositis: 33.7% vs 34.9%, *P* = 0.872)
	Mucositis	Dechaphunkul et al^116^	Baseline, day 43 of treatment, 1 month after chemotherapy	No difference was found between groups for mucositis (*P* = 0.690)
	Mucositis	Kuroki et al^123^	Weekly from start of CRT until 2 weeks after CRT completion	The intervention group had lower incidence of grade 3 mucositis vs the control (25.0% vs 64.7%, *P* = 0.0037) No significant differences reported for grade 2
	Mucositis	Muangwong et al^124^	Baseline through week 7	No differences in oral mucositis (aOR, 0.9; *P* = 0.79) or severity (aOR, 0.5; *P* = 0.14)
	Grade 3 mucositis	Chitapanarux et al^130^	Baseline to week 7	Percentage of grade 3 mucositis was lower in the intervention group vs the control but did not achieve significance (5% vs 20%, *P* = 0.342)
	Esophagitis	Muangwong et al^124^	Baseline through week 7	No differences in esophagitis (aOR, 0.7; *P* = 0.54)
	Mean days until mucositis	Diwan and Khan^97^	Start of radiation therapy to 9 weeks	Although all patients developed mucositis in both groups, the intervention group took longer to develop mucositis vs the control (32.86 days vs 17.06 days, no *P* value calculated)
	Dermatitis	Muangwong et al^124^	Baseline through week 7	No differences in dermatitis (aOR=0.7, *P* = 0.45) or severity (aOR=0.5) *P* = 0.16)
	Surgical wound infection	Sittitrai et al^126^	14 days post‐op	No significant difference between intervention and control (11.7% vs 19.6%, *P* = 0.307)
	General infection (respiratory tract infection, urinary tract infection, GI tract infection, and septicemia)	Sittitrai et al^126^	14 days post‐op	No significant difference between intervention and control (16.6% vs 25%, *P* = 0.468)
	Infections complications	Falewee et al^117^	7 days presurgery to 7–14 days post‐op	No difference between groups, although a per‐protocol analysis found decreased infection complications in the peri‐op immunonutrition group vs control (OR, 0.24; *P* = 0.05)
	Surgical site infections	Falewee et al^117^	7 days presurgery to 7–14 days post‐op	No difference between groups, although a per‐protocol analysis found decreased surgical site infections in the peri‐op immunonutrition group vs control (OR, 0.17; *P* = 0.04)
	Infections	Ghosh et al^120^	5 days presurgery to 30 days postsurgery	No difference was found for infections in the intervention vs control groups (43% vs 28%, *P* = 0.27)
	Mucocutaneous fistula	Sittitrai et al^126^	14 days post‐op	Mucocutaneous fistula was less in the intervention group vs the control (8.3% vs 23.2%, *P* = 0.039)
	Minor complications	Felekis et al^118^	Post‐op day 8	No difference in minor complications between the groups
	Major complications	Felekis et al^118^	Post‐op day 8	The control group had more major complications (two had pneumonia and one had a urinary tract infection) than the intervention group (1 wound infection) (*P* < 0.05)
	Hematologic toxicities grade 3–4	Chitapanarux et al^115^	Baseline to end of treatment	The intervention group had fewer hematologic toxicities grade 3–4 vs the control group (0/20 vs 5/20, *P* = 0.047
	Nonhematologic toxicities grade 3–4	Chitapanarux et al^115^	Baseline to end of treatment	The intervention group had fewer nonhematologic toxicities grade 3–4 vs the control group, but this did not achieve significance (1/20 vs 5/20, *P* = 0.182
	Severe grade 3–4 hematologic toxicities	Chitapanaruxa et al^130^	Baseline to week 7	Lower in control group vs intervention group (*P* = 0.035)
	Post‐op complications	Turnock et al^127^	Baseline to POD 10	One post‐op complication developed in the intervention group vs three in the control. No *P* value reported
	Overall local or systemic complications	Mueller et al^125^	5 days presurgery to 30 days postsurgery	Percentage of patients with local or systemic complications was lower in the intervention group compared with the control group (35% vs 58%, *P* = 0.027)
	Specific complications Wound dehiscence Wound abscess Fistula Local hematoma, hemorrhage, seroma	Mueller et al^125^	5 days presurgery to 30 days postsurgery	Local complications—including wound dehiscence (14% vs 20%, *P* = 0.41), wound abscess (12% vs 16%, *P* = 0.59), fistula (10% vs 18%, *P* = 0.25), and local hematoma, hemorrhage, or seroma (10% vs 11%, *P* = 0.83)—were fewer in the intervention group, but this did not achieve significance
	complications	Ascoli et al^113^	Pre‐op to 1 month post‐op	No differences were found between groups for general medical or surgical complications, but the intervention group had fewer major surgical complication (20% vs 6%, *P* = 0.0262)
	Post‐op complications	Kotb et al^122^	0, 4, 8, and 12 h post‐op	Intervention group had lower incidence of wound infection (*P* = 0.03), fever (*P* = 0.01), and neutropenia (*P* < 0.001) No difference in pulmonary infection between groups
	Surgery due to complications	Mueller et al^125^	5 days presurgery to 30 days postsurgery	No difference between intervention and control (20% vs 18%, *P* = 0.82)
	Thrombocytopenia	Chitapanaruxa et al^130^	Baseline to week 7	Rate of thrombocytopenia was lower in the intervention group vs the control (0% vs 5%, *P* = 0.035)
	Flap total or partial necrosis	Mueller et al^125^	5 days presurgery to 30 days postsurgery	No difference between intervention and control (9% vs 7%, *P* = 0.83)
	Radiation dermatitis	Imai et al^121^	First day of CCRT up to week 1	No difference between intervention vs control for incidence of grade ≥3 dermatitis (18.8% vs 11%, *P* = 0.44), but intervention group had lower incidence of grade ≥2 dermatitis (62.6% vs 94.4%, *P* = 0.03)
	Adverse events (abdominal discomfort, nausea/vomiting, diarrhea, and constipation)	Sittitrai et al^126^	14 days post‐op	No significant difference between intervention and control (18.4% vs 17.9%, *P* = 0.597)
	VAS pain	Kotb et al^122^	0, 4, 8, and 12 h post‐op	No difference in pain at any time point between groups
Readmissions	Readmissions	Mueller et al^125^	5 days presurgery to 30 days postsurgery	No difference between intervention and control *P* = 0.85)
	Readmissions	Ascoli et al^113^	Pre‐op to 1 month post‐op	No difference between groups (*P* = 0.765)
Length of stay	Length of post‐op hospital stay	Sittitrai et al^126^	14 days post‐op	Post‐op hospital stay was shorter in the intervention group vs the control (24.6 vs 29.8 days, *P* = 0.043)
	Post‐op length of stay	Ghosh et al^120^	5 days presurgery to 30 days postsurgery	No difference was found in hospital mean length of stay between intervention and control (31.1 vs 35.3 days, *P* = 0.73)
	Total length of stay	Mueller et al^125^	5 days presurgery to 30 days postsurgery	Length of stay was shorter in the intervention group vs the control (6 vs 17 days, *P* < 0.001)
	Median ICU length of stay	Turnock et al^127^	Baseline to unit discharge	No difference was found in median ICU length of stay between intervention and control (18.5 vs 19.5 days, *P* = 0.33)
	Median hospital length of stay	Turnock et al^127^	Baseline to unit discharge	No difference was found in median hospital length of stay between intervention and control (10 vs 21.5 days, *P* = 0.90)
	Hospital length of stay	Ascoli et al^113^	Pre‐op to 1 month post‐op	No difference between groups (*P* = 0.296)
	Hospital length of stay	Kotb et al^122^	Admission to discharge	No difference between intervention vs control (7.5 vs 7.76 days, *P* = 0.071)
Nutrition intake	Energy intake	Chitapanaruxa et al^130^	Baseline to week 7	Intervention group said to have significantly higher intake, but no statistic given
	Protein intake	Chitapanaruxa et al^130^	Baseline to week 7	Intervention group said to have significantly higher intake, but no statistic given
Survival	Overall survival Progression‐free survival	Boisselier et al^114^	3 years	No difference in survival at 3 years between groups. However, a placebo‐controlled subgroup analysis of participants adhering to treatment found increased 3‐year survival in the intervention group vs control (81% vs 61%, *P* = 0.034) and improved progression‐free survival (73% vs 50%, *P* = 0.012)
	2‐year survival	Chitapanarux et al^115^	Baseline to 2 years	A Kaplan‐Meier curve showed no difference between groups on 2‐year survival (*P* = 0.104)
	3‐year progression‐free and overall survival	Dechaphunkul et al^116^	Baseline, 3 years	The intervention group had better 3‐year progression‐free survival (*P* = 0.056) and overall survival (*P* = 0.065)
Treatment completion	CCRT completion rate	Chitapanarux et al^115^	Baseline to end of treatment	The intervention group had a higher CCRT completion rate vs the control group, but this did not achieve significance (17/20 vs 12/20, *P* = 0.155)
Weight loss/body composition	Median weight	Chitapanaruxa et al^130^	Baseline to week 7	No difference between groups, although weight lost occurred only in the control group (*P* = 0.001). Baseline differences for this variable were not accounted for
	Weight loss BMI loss	Yeh et al^128^	Baseline to week 8	No significant differences between groups
	Weight loss	Dechaphunkul et al^116^	Baseline, day 43 of treatment, 1 month after chemotherapy	No differences in weight loss between groups
	Weight loss	Kuroki et al^123^	Weekly from start of CRT until 2 weeks after CRT completion	The intervention group had less weight loss vs the control (5.6% vs 8.9%, *P* = 0.0038)
**Studies with no control (≥2 interventions)**				
Complications	Post‐op infectious complications	De Luis et al^108^	Baseline to 3 months	No significant difference was found between the EPA vs arginine group (0% vs 8.7%, *P* = NS)
	Fistula of wound Wound infection General infection Diarrhea	Casas‐Rodera et al^131^	Baseline, POD 7, POD 14, and once medically eligible for discharge	No significant between‐group differences
Nutrition intake	Energy and protein consumption	De Luis et al^108^	Baseline to 3 months	No difference between EPA and arginine group (*P* = NS)
Length of stay	Length of stay	Casas‐Rodera et al^131^	Baseline, POD 7, POD 14, and once medically eligible for discharge	No significant between‐group differences

Abbreviations: aOR, adjusted odds ratio; BIA, bioelectrical impedance analysis; BMI, body mass index; CCRT, concurrent chemoradiotherapy; CRT, chemoradiotherapy; DHA, docosahexaenoic acid; DXA, dual‐energy x‐ray absorptiometry; EORTC, European Organisation for Research and Treatment of Cancer; EPA, eicosapentaenoic acid; GI, gastrointestinal; HR, hazard ratio; ICU, intensive care unit; MDASI‐HN, MD Anderson Symptom Inventory–Head & Neck; NRS‐2002, Nutritional Risk Screening 2002; NS, not significant; OHIP‐14, Oral Health Impact Profile‐14; OR, odds ratio; peri‐op, perioperative; PG‐SGA, Patient‐Generated Subjective Global Assessment; POD, postoperative day; post‐op, postoperative; pre‐op, preoperative; QLQ‐C30, Quality of Life Questionnaire–Core 30; QLQ‐H&N35, Quality of Life Questionnaire–Head & Neck 35; QOL, quality of life; RT, radiotherapy; ST, systemic therapy; VAS, Visual Analog Scale; WHO, World Health Organization (Oral Toxicity Scale).

### Glutamine

Fifteen studies that met the inclusion criteria for this question examined the use of glutamine.^93^, ^94^, ^95^, ^96^, ^97^, ^98^, ^99^, ^100^, ^101^, ^102^, ^103^, ^104^, ^105^, ^106^, ^129^ Twelve studies demonstrated a significant benefit from glutamine supplementation compared with the control.^93^, ^94^, ^95^, ^96^, ^98^, ^100^, ^101^, ^102^, ^103^, ^104^, ^105^, ^129^ Seven studies reported reduced severity of mucositis^93^, ^95^, ^96^, ^98^, ^100^, ^101^, ^105^; one reported lower incidence of mucositis^103^; one reported increased time to mucositis onset^98^; two reported less severe dysphagia^103^, ^104^; four reported less pain^93^, ^95^, ^100^, ^105^; one reported less severe odynophagia^129^; one reported less hospitalization for treatment toxicities, less weight loss, and fewer treatment interruptions^103^; one reported less nausea and less edema^104^; two reported improved quality of life^93^, ^94^; one reported improved fat‐free mass^94^; and one reported less dermatitis^102^ in the intervention group. Two studies found no significant difference between the two groups for any outcome (Tables 22, 23, and S27–S29). Importantly, one study reported on overall and progression‐free survival for parenteral glutamine and found no difference between groups.^106^ Six studies were rated as having low risk of bias.^93^, ^102^, ^103^, ^104^, ^105^, ^106^ Two were rated as having high risk of bias,^96^, ^100^ and the remaining seven studies were rated as having some concerns.

**Figure 7:**
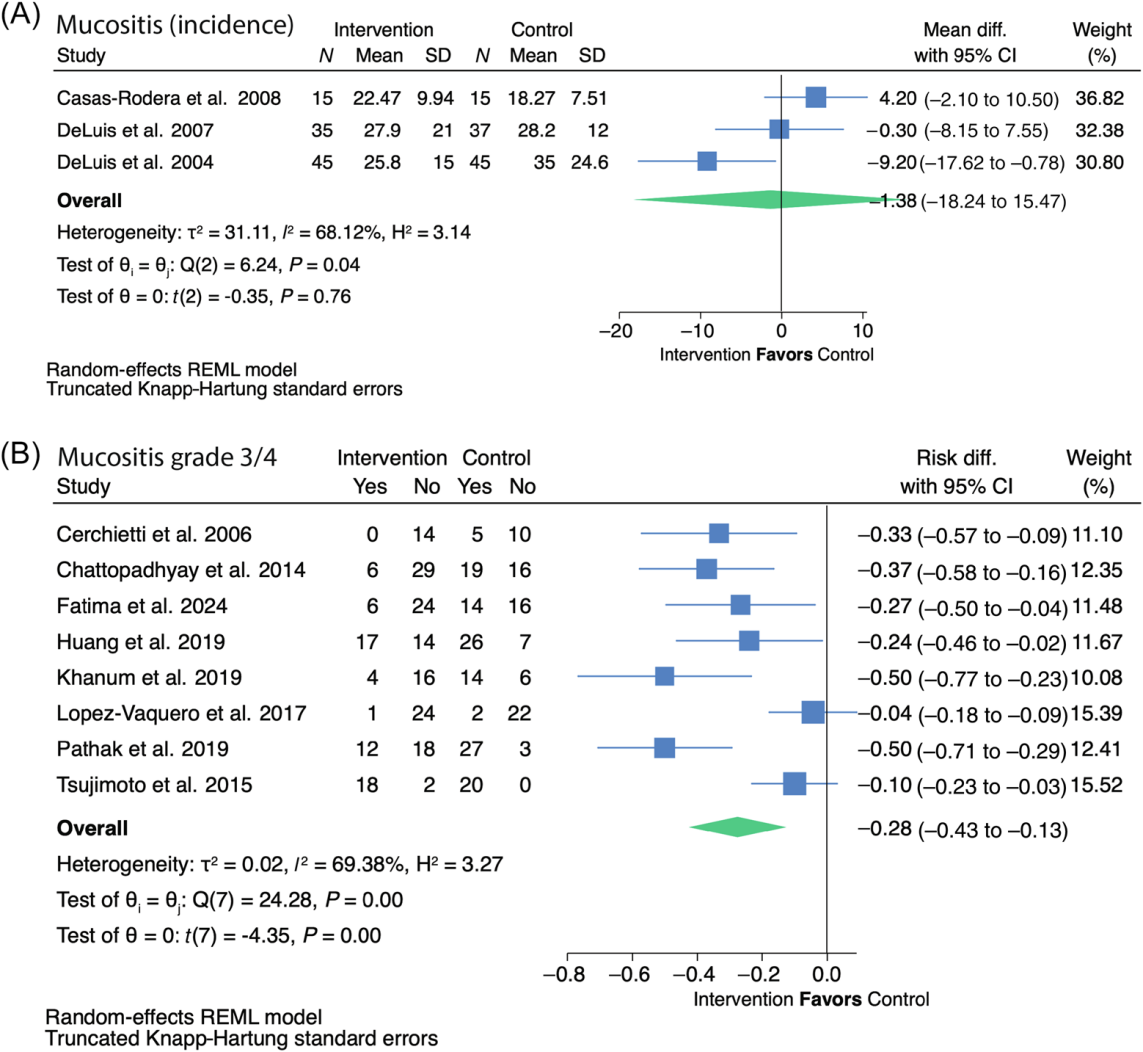
Forest plots for question 10—glutamine outcomes. Forest plot (A) shows results for the effect of glutamine incidence of mucositis, whereas forest plot (B) shows the effect of glutamine on the severity of mucositis, specifically the incidence of patients reaching mucositis grade 3 or 4. diff., difference; REML, restricted maximum likelihood.

Meta‐analysis was possible for two outcomes: mucositis and mucositis severity Grade 3–4 (Figure 7). On average, patients were reported to have 5% less mucositis in the glutamine intervention groups vs the controls, but this was not statistically significant (RD = −0.05, 95% CI = −0.12 to 0.03; *P* = 0.16). Risk of having mucositis Grade 3–4 was 28% reduced in the glutamine intervention groups vs the control (RD = −0.28, 95% CI = −0.43 to −0.13; *P* < 0.001).

### ω‐3

**Figure 8:**
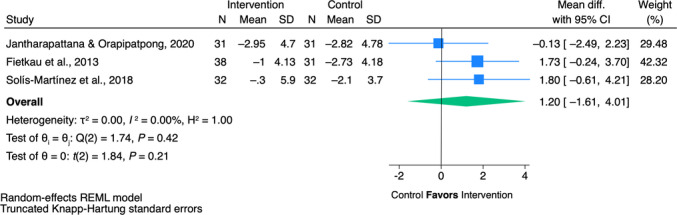
Forest plot for question 10—ω‐3 outcomes: absolute weight loss in kilograms. diff., difference; REML, restricted maximum likelihood.

Seven studies that met the inclusion criteria for this question examined the use of ω‐3 fatty acids.^107^, ^108^, ^109^, ^110^, ^111^, ^112^, ^119^ Three studies demonstrated a significant benefit from ω‐3 supplementation.^107^, ^112^, ^119^ One study reported improved nutrition status and improved appetite at 6‐ to 7‐week follow‐up,^119^ one reported less fatigue,^112^ and one reported higher energy intake and higher body weight at baseline, end of radiotherapy, and at 1 and 3 months posttreatment, improved quality of life, and higher treatment completion^107^ in the intervention group. The remaining three studies found no significant difference between the two groups for any outcome (Tables 22, 23, S27, and S28). Two studies were found to be at “low” bias risk,^107^, ^111^ and the rest were at “some concerns” for bias. Cereda et al and Pottel et al were rated as having low risk of bias, Solís‐Martínez was rated as having high risk of bias, and the remaining three studies were rated as having some concerns.^107^, ^111^ Meta‐analysis was possible for absolute weight loss (Figure 8). On average, patients in the ω‐3 intervention group had 1.2 kg less weight loss, but this was not statistically significant (MD = 1.20 kg, 95% CI = −1.61 to 4.01; *P* = 0.21).

### Combined special‐purpose nutrient formulas

Sixteen studies that met the inclusion criteria for this question examined the use of combined special‐purpose nutrient formulas.^114^, ^115^, ^117^, ^120^, ^121^, ^122^, ^125^, ^126^, ^127^, ^128^, ^130^ Twelve studies demonstrated a significant benefit from combined immunonutrition supplementation.^113^, ^115^, ^116^, ^117^, ^118^, ^121^, ^122^, ^123^, ^125^, ^126^, ^130^ Nine studies reported fewer complications.^113^, ^115^, ^117^, ^118^, ^121^, ^122^, ^125^, ^126^, ^130^ One reported lower incidence of Grade 3 mucositis.^123^ Two reported reduced postoperative or total length of hospital stay.^125^, ^126^ One reported higher energy and protein intake.^130^ Two reported increased 3‐year survival in the intervention group,^114^, ^116^ with one reporting improved overall survival^116^ and the other reporting better progression‐free survival in a subanalysis of compliant participants.^114^ One study found better weight retention in the intervention group vs the control.^123^ Three studies found no significant difference between the two groups for any outcome (Tables 22, 23, and S27–S29). Four studies were rated as having a “low” risk of bias.^114^, ^116^, ^117^, ^127^ Six studies were rated as having a “high” risk of bias,^113^, ^115^, ^123^, ^124^, ^125^, ^130^ and the remaining studies were rated as having “some concerns.”

### Rationale and discussion

As described in a narrative review, special‐purpose nutrients have a role in modulating reactions within immune, inflammatory, and lean muscle–producing pathways.^132^ Through such mechanisms, special‐purpose nutrients may have an effect on outcomes such as nutrition status, muscle mass, wound healing, and other complications.^133^, ^134^ To determine whether a specialty nutrient should be administered, a careful weighing of the benefits vs harms is needed.

The benefits of arginine were clear in the literature, with its ability to decrease incidence of fistula, decrease length of stay, and improved progression‐free and overall survival. Arginine has been associated with some negative outcomes such as headache, infection, gastrointestinal disturbances, and rash. However, these only occur in approximately 3% of the population.^135^ For this reason, we felt the benefits of taking arginine outweighed any potential harms.

Glutamine is a more complex issue. Glutamine, not to be confused with glutamate (eg, L‐glutamic acid), is not usually present in pharmacologic doses in standard care enteral or parenteral products. This is because of its stability issues in an aqueous environment. It is available as a powdered modular product. Outside the United States, it is also available as an intravenous parenteral supplement in its more stable dipeptide form. The literature found glutamine to be very effective in delaying, reversing, and decreasing the severity of mucositis. However, certain concerns have arisen in the past few years that glutamine could itself promote tumor growth and progression as well as resistance to cancer treatment. In 2022, a compelling narrative review was published by Alden et al demonstrating the current state of evidence concerning the potential for glutamine to feed tumor growth and induce resistance to cancer therapy.^136^ The mechanisms were biologically plausible. They were supported entirely by mechanistic studies showing how glutamine supports cancer cell survival, preclinical studies showing radiosensitization and improved immunotherapy efficacy when glutamine metabolism is blocked, and early clinical trials assessing the combination of glutamine inhibitors with standard treatments.^136^ Furthermore, an RCT in patients undergoing autologous stem cell transplant found worse mortality in the intervention group who received 30 g/day of parenteral glutamine, although this study was very small (*n* = 40).^137^ Conversely, a recent oral glutamine intervention study in patients with head and neck cancer reported no differences in survival. Although this study was also very small (*n* = 38), it directionally favored the oral glutamine group,^106^ and similar findings have been reported in other cancer populations. One glutamine study in non–small cell lung cancer (*n* = 101) found no significant difference in overall or progression‐free survival, with the glutamine group directionally favored at most time points of survival analysis.^138^ Another study (*n* = 122) in a similar population found the glutamine group did not have worse overall or disease‐free survival.^139^ After adjusting for weight loss, low hemoglobin level, and nodal stage, both overall (*P* = 0.05) and disease‐free survival (*P* = 0.035) were significantly higher than those of the control. These findings are corroborated in a smaller study in 60 women with breast cancer. Oral glutamine did not negatively impact tumor shrinkage or immunohistochemistry.^140^ Although this literature is still in its infancy and more research is needed, there is little to suggest that oral glutamine is problematic and much to suggest it is helpful in treating treatment toxicities. For this reason, we suggest the use of glutamine is acceptable with the caveat that caution is needed concerning parenteral glutamine unless future findings demonstrate its safety. This caution is consistent with the recommendation in a clinical guideline for the management of mucositis secondary to cancer therapy.^141^

The reported benefits of ω‐3 supplementation were minor in the literature, encompassing decreased fatigue, improved dietary intake, and weight gain. Potential ω‐3 side effects are also minor and include gastrointestinal upset, abdominal pain, rash, infection, and taste changes.^142^ The current recommendation for ω‐3 is based on the potential benefit, along with some evidence of benefit from the literature, and no serious harms.

The studies on combined immunonutrition examined various blends of immunonutrients. The data imply these formulas may be beneficial, although it is difficult to know which ingredient is performing the benefit, and different formulations were used across the different studies. Currently, all the premade immunonutritions formulations are enteral. If parenteral immunonutrition is formulated, we recommend against including dipepetide glutamine parenteral formulations until further research demonstrates its safety.

### Future research

Data regarding the impact of special‐purpose nutrients on survival are limited. In addition, current studies vary in their quality and the outcomes assessed. Adequately powered, double blind randomized trials examining the effect of supplementation with special‐purpose nutrients compared with standard care on treatment complications, hospital length of stay, treatment completion, and progression‐free and overall survival are required. This is particularly crucial for glutamine supplementation because of its potential effects on tumor growth, cancer progression, and treatment resistance. Large RCTs are needed to assess the safety of glutamine but especially of parental dipeptide glutamine formulations.

## CONCLUSION

This clinical guideline has built on current guidelines and is intended to support clinicians in delivering optimal nutrition care to patients with head and neck cancer. The recommendations are based on evidence from RCTs, quasi‐experimental designs, and expert opinion. The strongest evidence was found for the frequency of dietitian intervention, intensity of nutrition therapy, timing of EN, frequency of speech pathology intervention, and an interdisciplinary approach to nutrition management. It is anticipated these guidelines will be used to advocate for sufficient resources within health services to provide evidence‐based care to people with head and neck cancer. During the synthesis of data from the included trials, it was evident that standardized outcome reporting is lacking in this field, meaning meta‐analysis was only possible for a limited number of questions. We call on researchers in the field of head and neck cancer nutrition to work toward a standardized set of outcome measures to improve the quality of evidence and ability to synthesize the data underpinning future recommendations. Furthermore, suggestions for further interdisciplinary research, appropriate study designs, and outcomes were identified within each rationale above to encourage ongoing research in the nutrition care for this vulnerable population.

## AUTHOR CONTRIBUTIONS

**Nicole Kiss**: Conceptualization; Writing—original draft; Data curation; Supervision; Writing—review and editing. **Merran Findlay**: Conceptualization; Writing—review and editing. **Jacqui Frowen**: Conceptualization; Writing—original draft; Writing—review and editing; Data curation. **Whitney E. Lewis**: Conceptualization; Writing—review and editing; Data curation. **Jeannine Mills**: Conceptualization; Data curation; Writing—review and editing. **Anurag K. Singh**: Conceptualization; Writing—review and editing; Data curation. **David D. Church**: Data curation; Conceptualization; Writing—review and editing. **Jacob T. Mey**: Conceptualization; Data curation; Writing—review and editing. **Sarah Peterson**: Data curation; Writing—review and editing; Conceptualization. **Kathleen Aguzzi**: Data curation; Writing—review and editing. **Sarah Bellini**: Data curation; Writing—review and editing. **Maria Paula Villela Coelho**: Writing—review and editing; Data curation. **Laura Cordwin**: Writing—review and editing; Data curation. **Michael Duffy**: Writing—review and editing; Data curation. **Shanna Hager**: Writing—review and editing; Data curation. **Manpreet S. Mundi**: Writing—review and editing; Data curation. **Michael Owen‐Michaane**: Writing—review and editing; Data curation. **Kathleen Price**: Writing—review and editing; Data curation. **Heather Stanner**: Writing—review and editing; Data curation. **Bridget Storm**: Writing—review and editing; Data curation. **Malika Udagedara**: Writing—review and editing; Data curation. **Liam McKeever**: Conceptualization; Writing—original draft; Methodology; Validation; Software; Formal analysis; Project administration; Data curation; Supervision.

## CONFLICT OF INTEREST STATEMENT

Nicole Kiss has received honoraria from Nutricia Australia and Abbott Australasia for consultancy and presentations. Merran Findlay has received honoraria from Fresenius Kabi Australia and Nutricia Australia for invited presentations and was supported by a Maridulu Budyari Gumal (SPHERE) Cancer Clinical Academic Group Senior Research Fellowship funded by a Cancer Institute NSW Research Capacity Building Grant (2021/CBG003). Whitney E. Lewis accepted a position as a medical liason at Bristol Myers Squibb, USA, near the end of the guideline creation period. She was consulted for her expertise but did not provide a Delphi vote for any recommendation. Manpreet S. Mundi reports no conflict of interest pertinent to this topic; there are research grants to his institution from Nestlé and NorthSea. The remaining authors declare no conflict of interests.

## Supporting information

Supplemental Appendix.
