# Supplementary material for: Guidelines for nutrition in adults with head and neck cancer: The American Society for Parenteral and Enteral Nutrition
Source: JPEN J Parenter Enteral Nutr. 2026 Mar 3;50(3):274–338. doi: 10.1002/jpen.70067 (PMC13047306; doi:10.1002/jpen.70067)
Supplement: Supplementary file 1 — Supplemental Appendix. [file JPEN-50-274-s001.docx]

**Supplemental Appendix**

The Tables below represent some of the data the team considered when making their decisions. In most cases, some degree of expert opinion was required to account for the inconsistency or lack of data. It should be noted that two bias analyses were performed for each study to account for the differences between the bias for a variable that is or is not vulnerable to blinding status. In the body of the paper, only one bias result was selected according to what was considered most important to the question.

**Table S1 GRADE Summary of Findings Table for Question 1a**

| **Outcomes** | **№ of participants (studies) Follow-up** | **Certainty of the evidence (GRADE)** | **Relative effect (95% CI)** | **Anticipated absolute effects** | |
| --- | --- | --- | --- | --- | --- |
|  |  |  |  | **Risk with later enteral nutrition** | **Risk difference with earlier enteral nutrition** |
| 1 Year Mortality | 516  (4 RCTs) | ⨁⨁⨁◯  Moderatea | **OR 0.7558**  (0.5016 to  1.1275) | **Study population** | |
|  |  |  |  | 13 per 100 | **3 fewer per**  **100**  (6 fewer to 1 more) |
|  |  |  |  | **Moderate** | |
|  | | |  | 13 per 100 | **3 fewer per**  **100**  (6 fewer to 1 more) |
| Weight and Body Composition Changes | (5 RCTs) | ⨁⨁⨁◯  Moderateb | Five studies examine changes in weight, BMI, fat- free mass, and Nutrition Status. Outcome heterogeneity precluded statistical conflation. No significant differences were found in any case.  However, in one study (Silander et al., 2012), at 6 months post-treatment, the study group had lost  8.8 kg (11.2%) compared to the control group, which lost 9.6 kg (12.4%; p=0 .08). However, this was not durable at the one and two-year mark. | | |
| Complications | (2 RCTs) | ⨁⨁◯◯  Lowb | Two studies examined general complications with one examining unplanned hospital admissions.  No significant between group differences were found for any complication. | | |
| Dysphagia | (2 RCTs) | ⨁⨁⨁◯  Moderateb | Two studies reported on this outcome. One found no differences (Silander et al., 2013) but a 2012 study by the same author examined the outcomes in a larger sample size and found significant differences. In this larger study, dysphagia rose in both groups to 28-29% after month 3. After 1 year, 1 still had the problem in the intervention compared to 9 in the control group (p=0.047). At 1 year follow-up, 20% in Control compared to 7% in the study group still had dysphagia. | | |
| Energy Intake | (1 RCT) | ⨁⨁⨁◯  Moderateb,c | One study reported on energy intake and found no difference between groups. | | |
| Quality of Life | (3 RCTs) | ⨁⨁⨁◯  Moderateb,c | Three studies examined Quality of Life spanning multiple scales. Reporting methods precluded data conflation. Silander et al. (2012) showed improvements in physical functioning at 2-month follow-up and better social functioning, appetite loss, constipation, and diarrhea in the study group. At 6-Month follow-up, the most notable differences were observed, with 10 significant improvements in the study group, including global QoL, physical, cognitive, and role functioning, and reduced fatigue, dyspnea, feeling ill, coughing, mouth opening, and sexuality. Many improvements endured at 1 and 2 year follow-ups. Salas et al (2009) reported worse physical quality of life immediately post- gastrostomy but improved mental health by the end of the study in the study group. This group found no differences in global quality of life.  Brown et al (2017b). Found no differences at any time point for any domain. | | |
| Other Mortality Time Points | (4 RCTs) | ⨁⨁⨁◯  Moderated | Four studies looked at mortality rates at different time points as far out as 2 years. None found any significant differences. | | |
| Disease-free Survival | (1 RCT) | ⨁⨁⨁◯  Moderateb,c | In this single study, there were 19 cases of disease relapse in the standard care group and 10 cases in the intervention group (p= 0.135). | | |
| Treatment Tolerance/ Completion | (2 RCTs) | ⨁⨁⨁◯  Moderateb,c | One study found no differences between groups, while another study reported 1 treatment interruption in the study group vs 7 in the control (p=0.08). | | |
| Tube Removal | (2 RCTs) | ⨁⨁⨁◯  Moderateb,c | No between group differences in tube removal were found for either study. | | |
| Hospital Length of Stay (HLOS) | (1 RCT) | ⨁⨁⨁◯  Moderatec | Length of stay was 5.85 days shorter in the intervention vs the control (p=0.001) | | |

***The risk in the intervention group** (and its 95% confidence interval) is based on the assumed risk in the comparison group and the **relative effect** of the intervention (and its 95% CI).

**CI:** confidence interval; **OR:** odds ratio

**GRADE Working Group grades of evidence**

**High certainty:** we are very confident that the true effect lies close to that of the estimate of the effect.

**Moderate certainty:** we are moderately confident in the effect estimate: the true effect is likely to be close to the estimate of the effect, but there is a possibility that it is substantially different.

**Low certainty:** our confidence in the effect estimate is limited: the true effect may be substantially different from the estimate of the effect. **Very low certainty:** we have very little confidence in the effect estimate: the true effect is likely to be substantially different from the estimate of effect.

**Explanations**

1. Confidence intervals contained both MID for benefit and for harm. A third point was not deducted because the OIS was not met and so this crossing of MID into harm may just be an artifact of unstable confidence intervals.
2. Studies did not permit meta-analysis, and so the certainty that may be gained from combining these effects is unknown.
3. Combined sample size <400
4. No timepoint met the OIS and so the confidence intervals are likely unstable. For this reason, 2 but not 3 points were deducted.

**Table S2: Question 1a Randomized Control Trials (ROB2)**

**
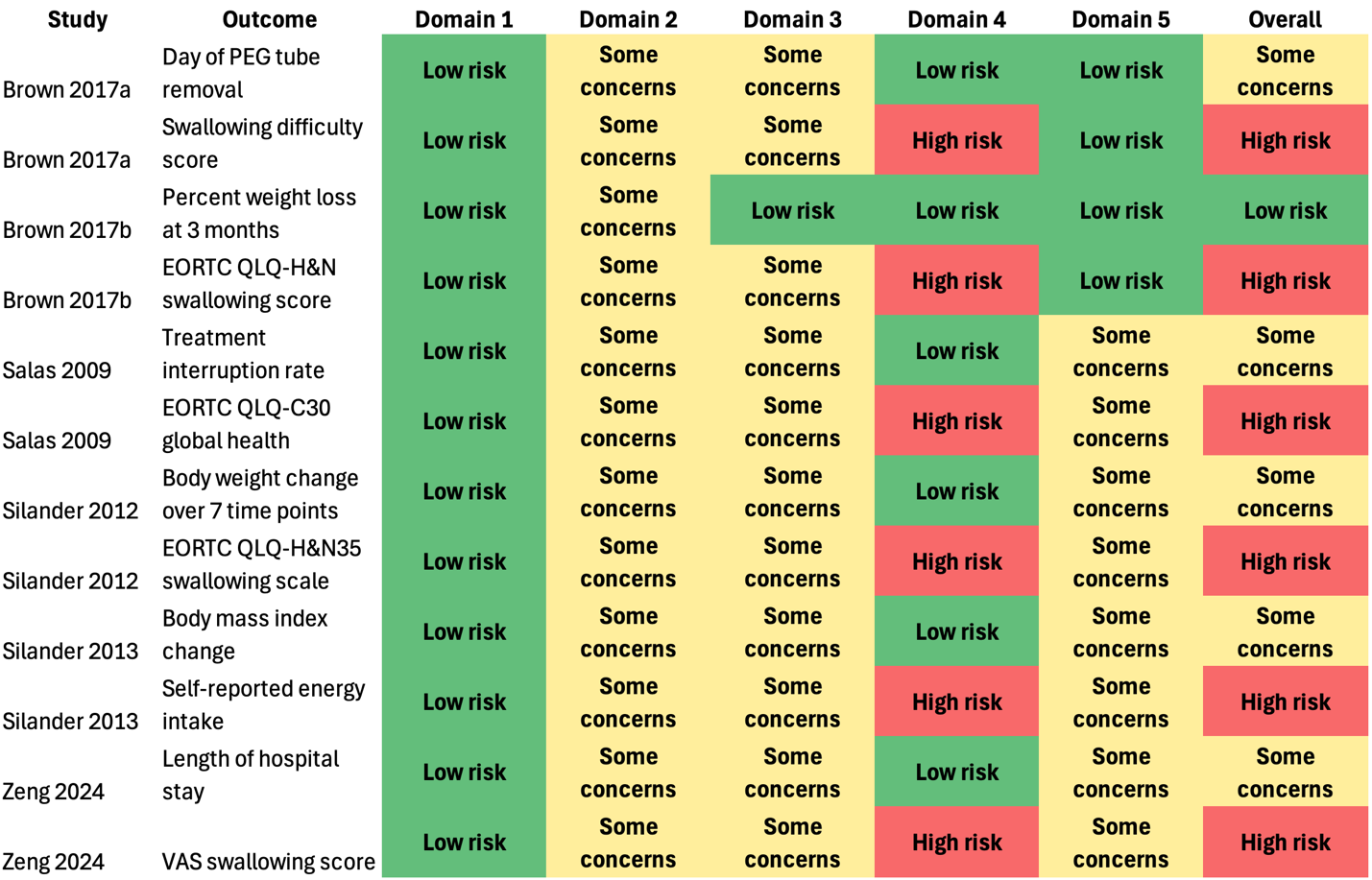
**

**Table S3 Question 1b GRADE Summary of Findings**

**
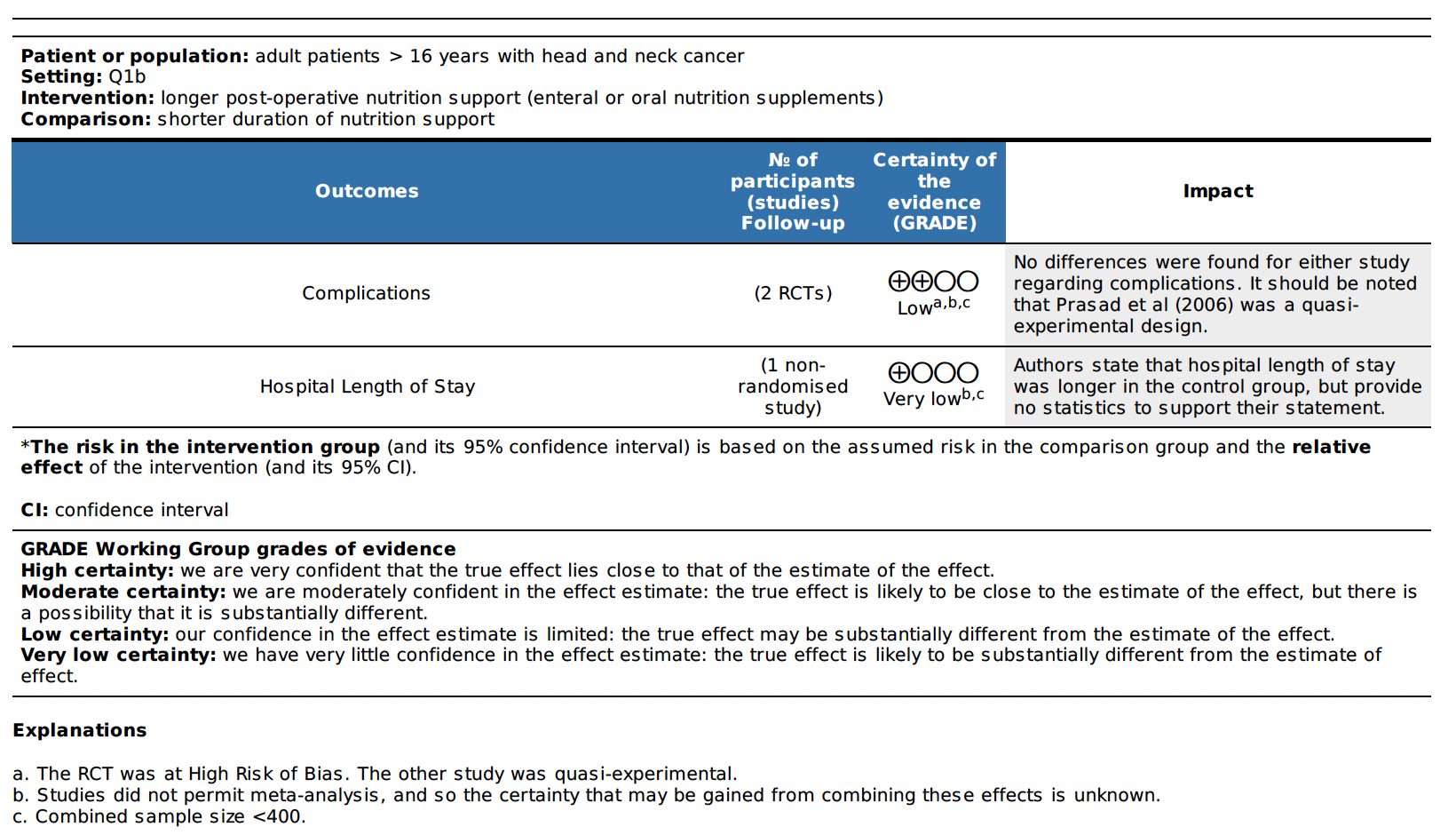
**

**Table S4 Question 1b Randomized Control Trials (ROB2)**

**
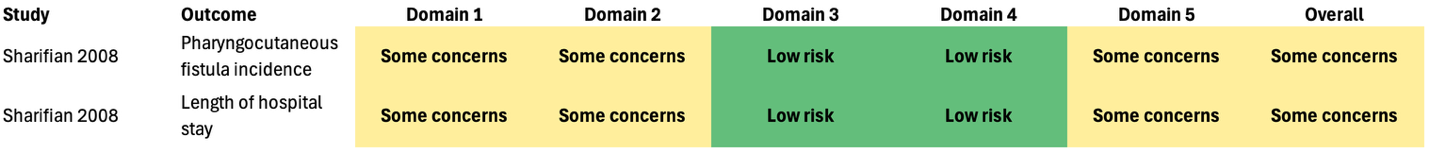
**

**Table S5 Question 1b Quasi-experimental Designs (ROBINS-I)**

**
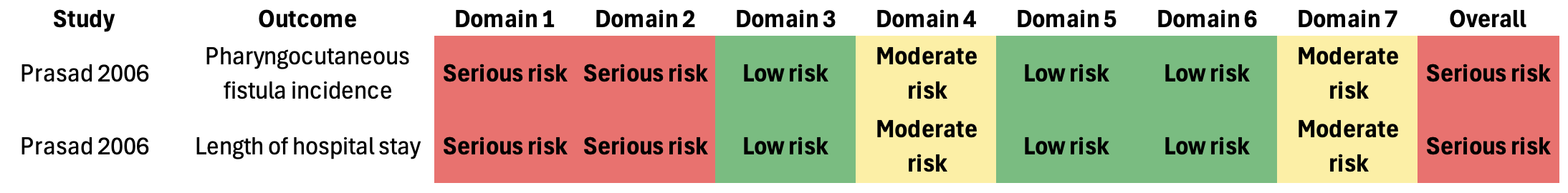
**

**Table S6 Question 1c Summary of Findings Table**

**
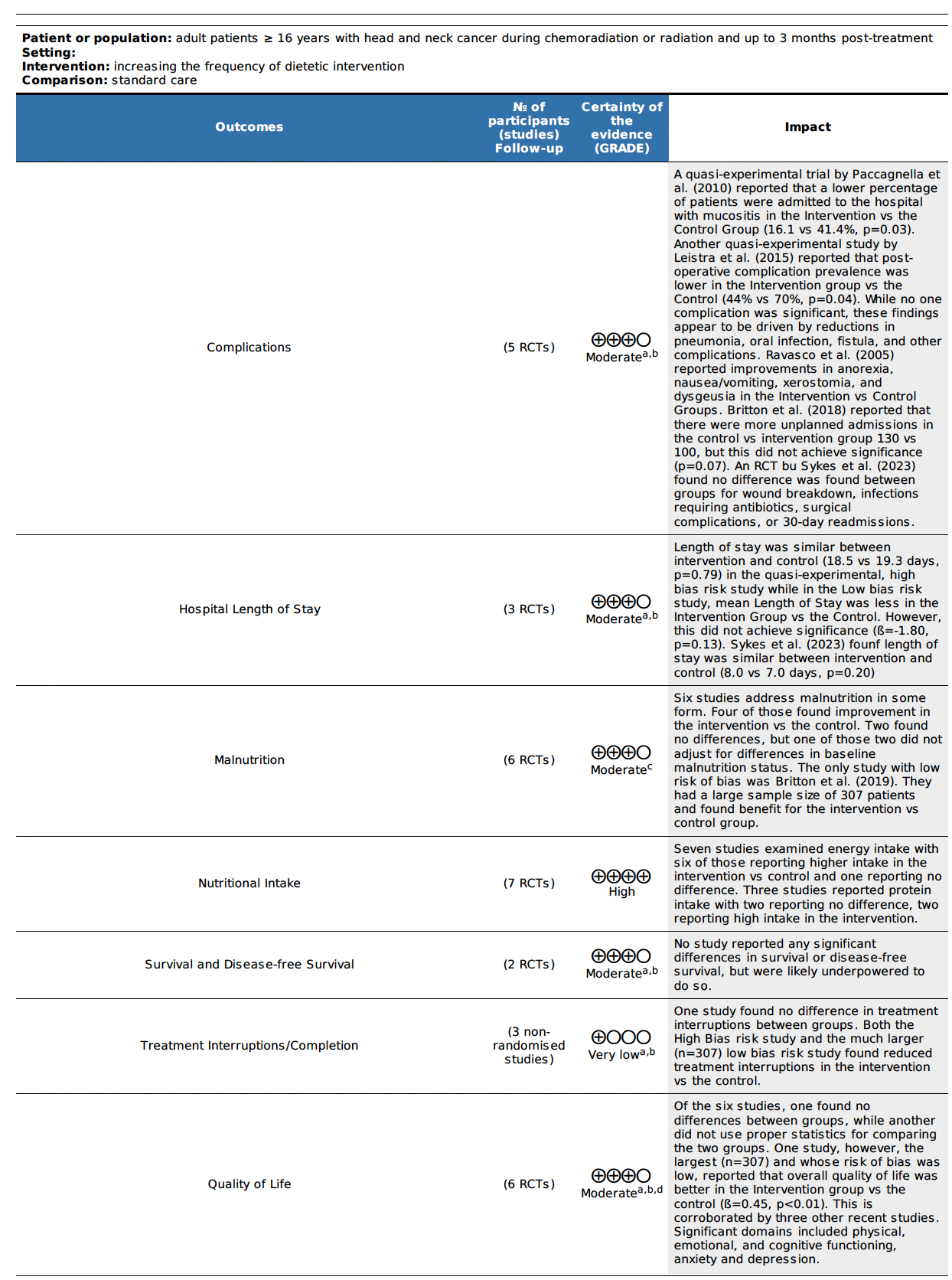
**

**
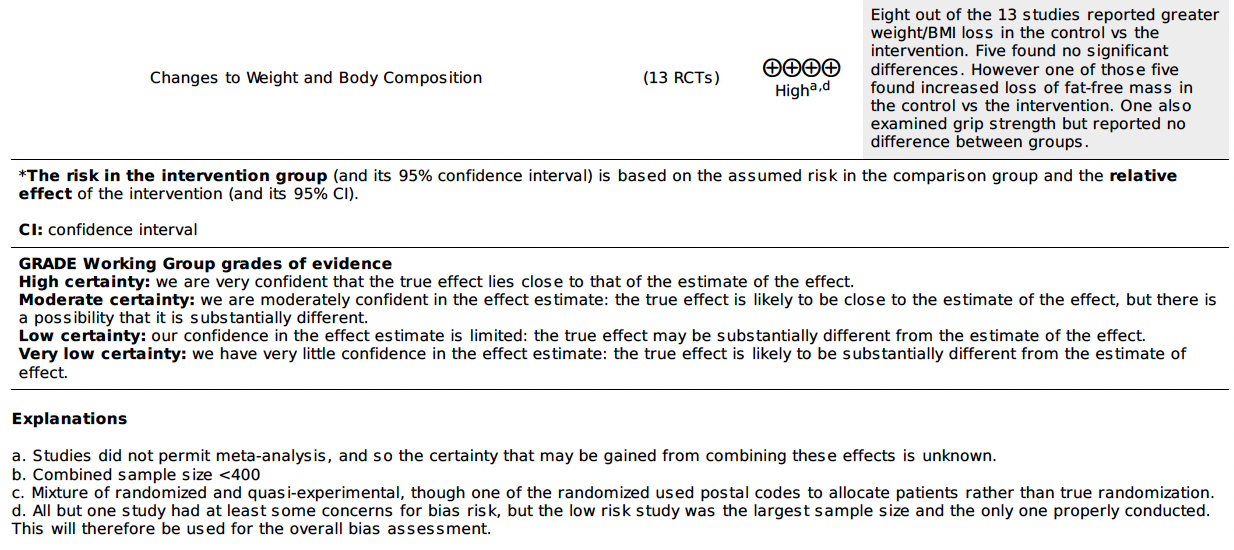
**

**Table S7: Question 1c Randomized Control Trials (ROB2)**

**
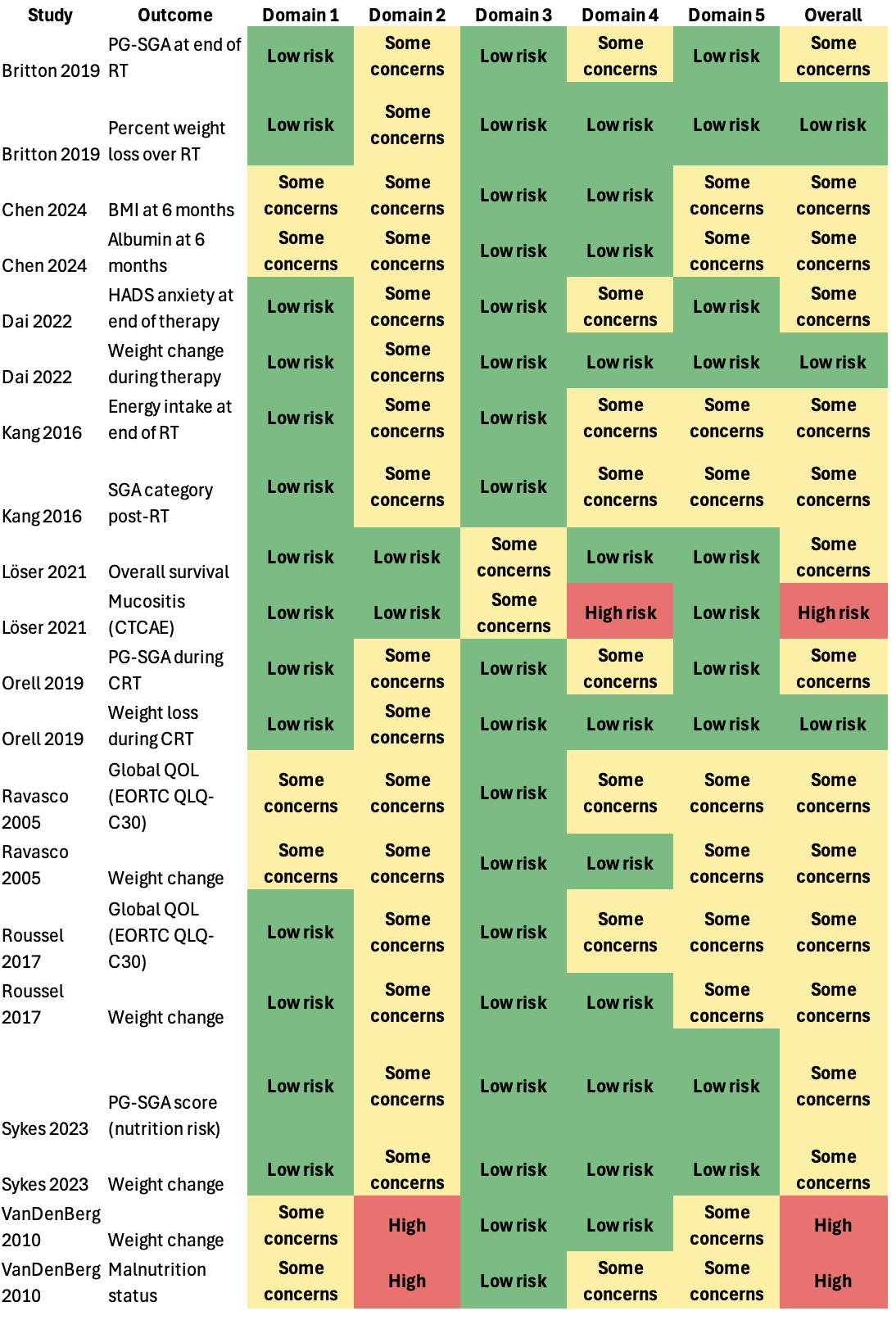
**

**Table S9: Question 1c Quasi-Experimental Studies (ROBINS-I)**

**
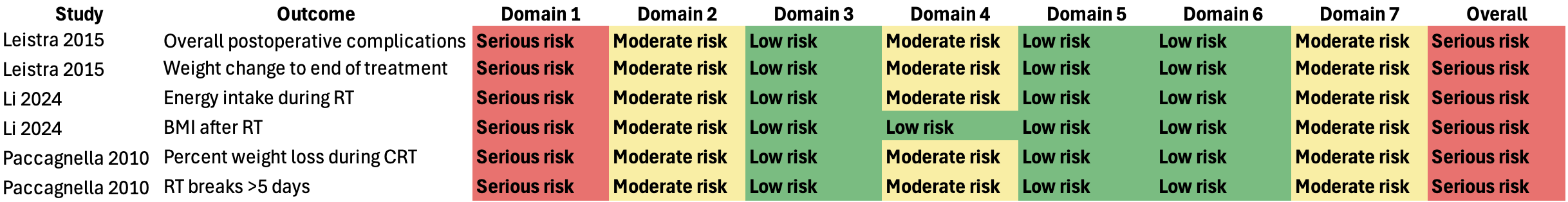
**

**Table S10: Question 2b GRADE Summary of Evidence**

**
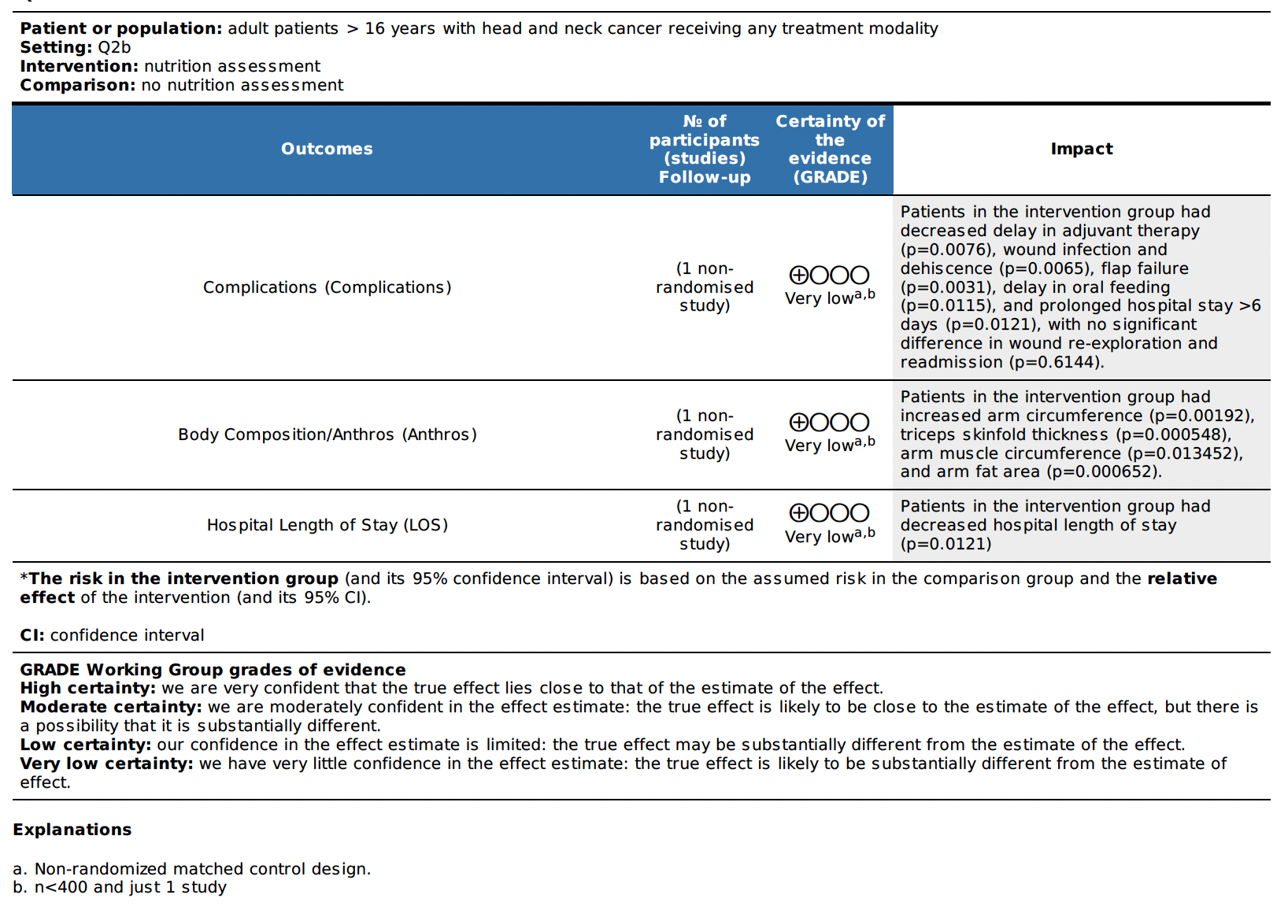
**

**Table S11: Question 2b Quasi-Experimental Studies (ROBINS-I)**

**
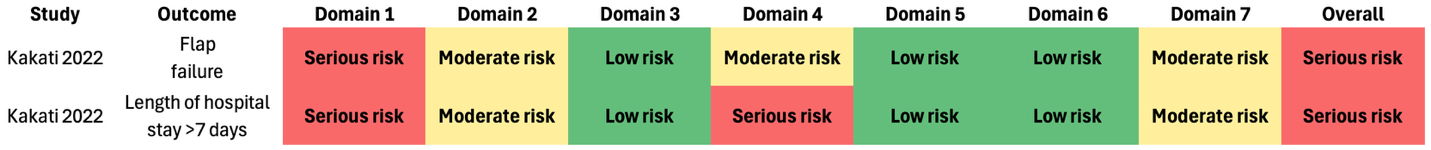
**

**Table S12: Question 3a GRADE Summary of Findings**

**
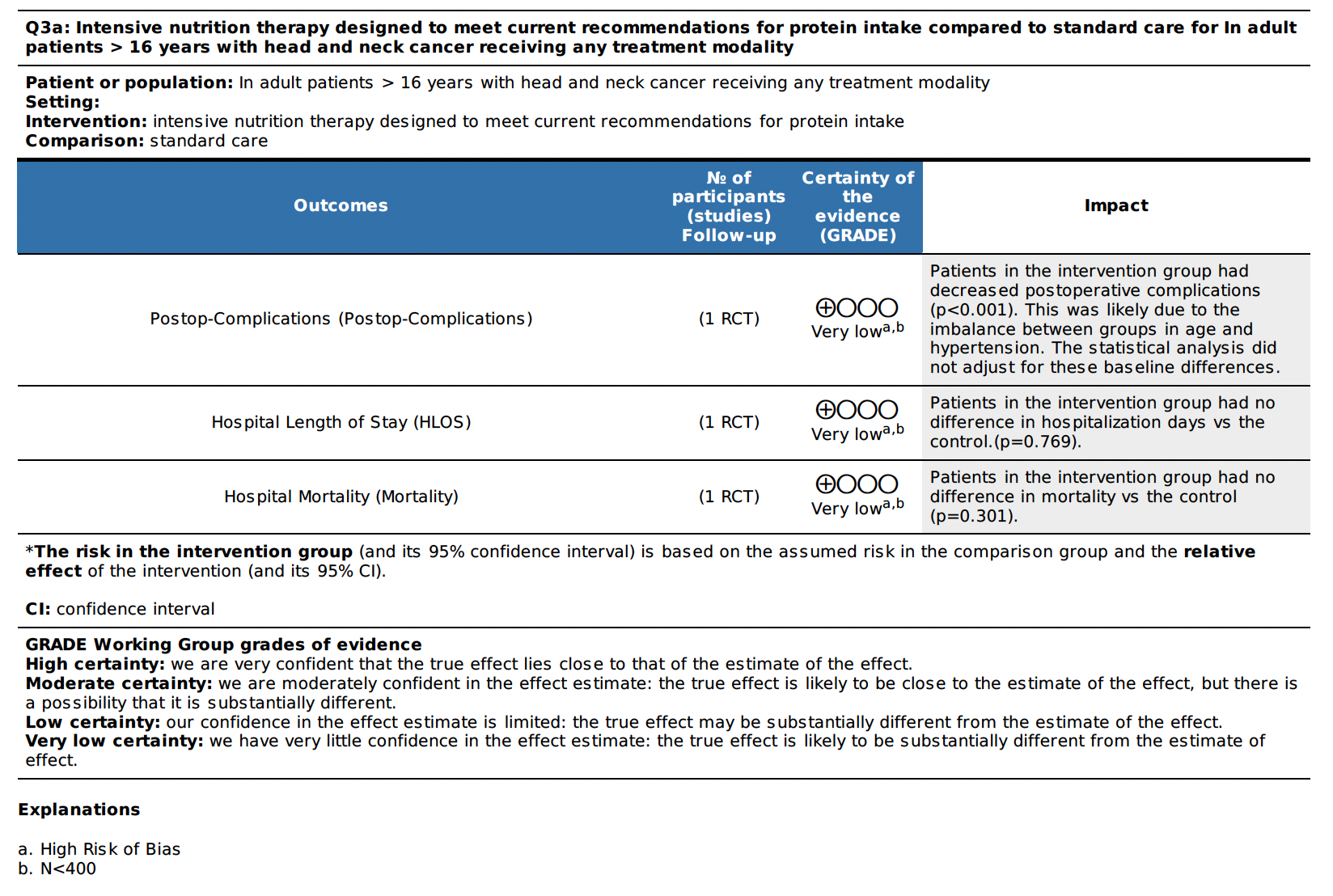
**

**Table S13Question 3a Randomized Control Trials (ROB2)**

**
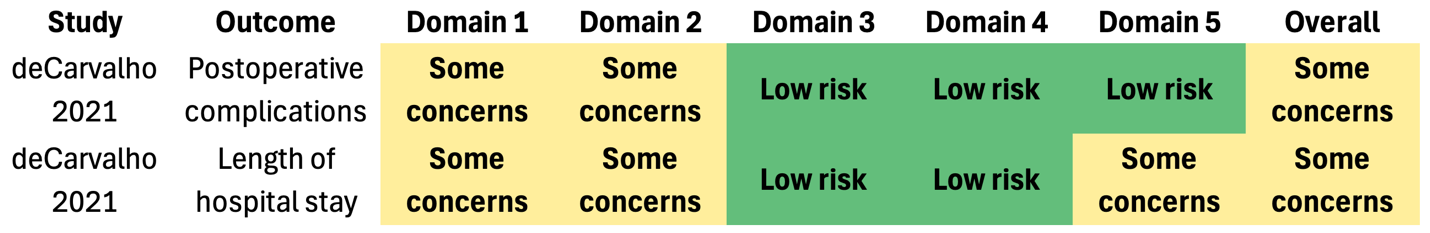
**

**Table S14 Question 3b GRADE Summary of Findings**

**
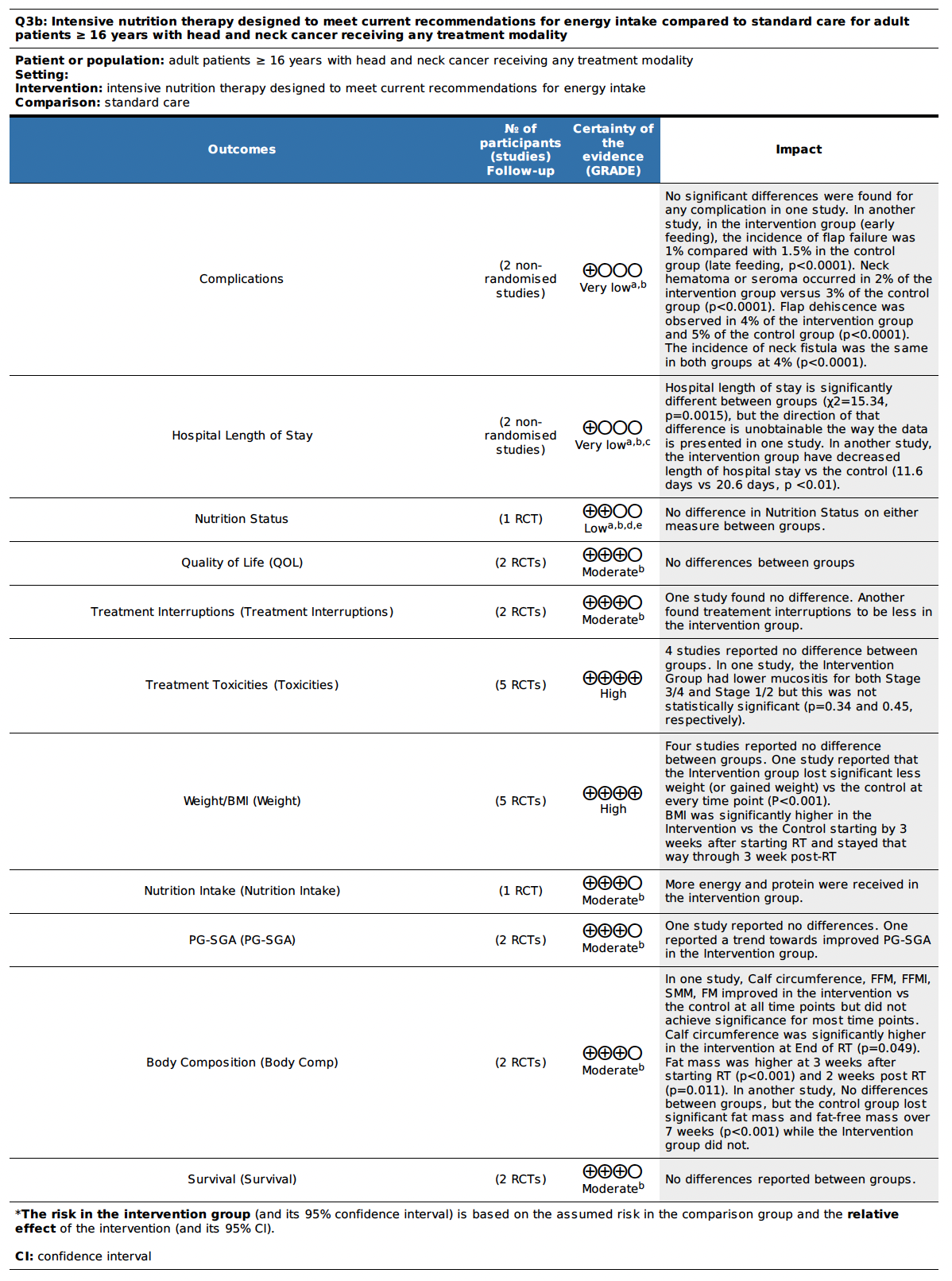
**

**Table S15: Question 3b Randomized Control Trials (ROB2)**

**
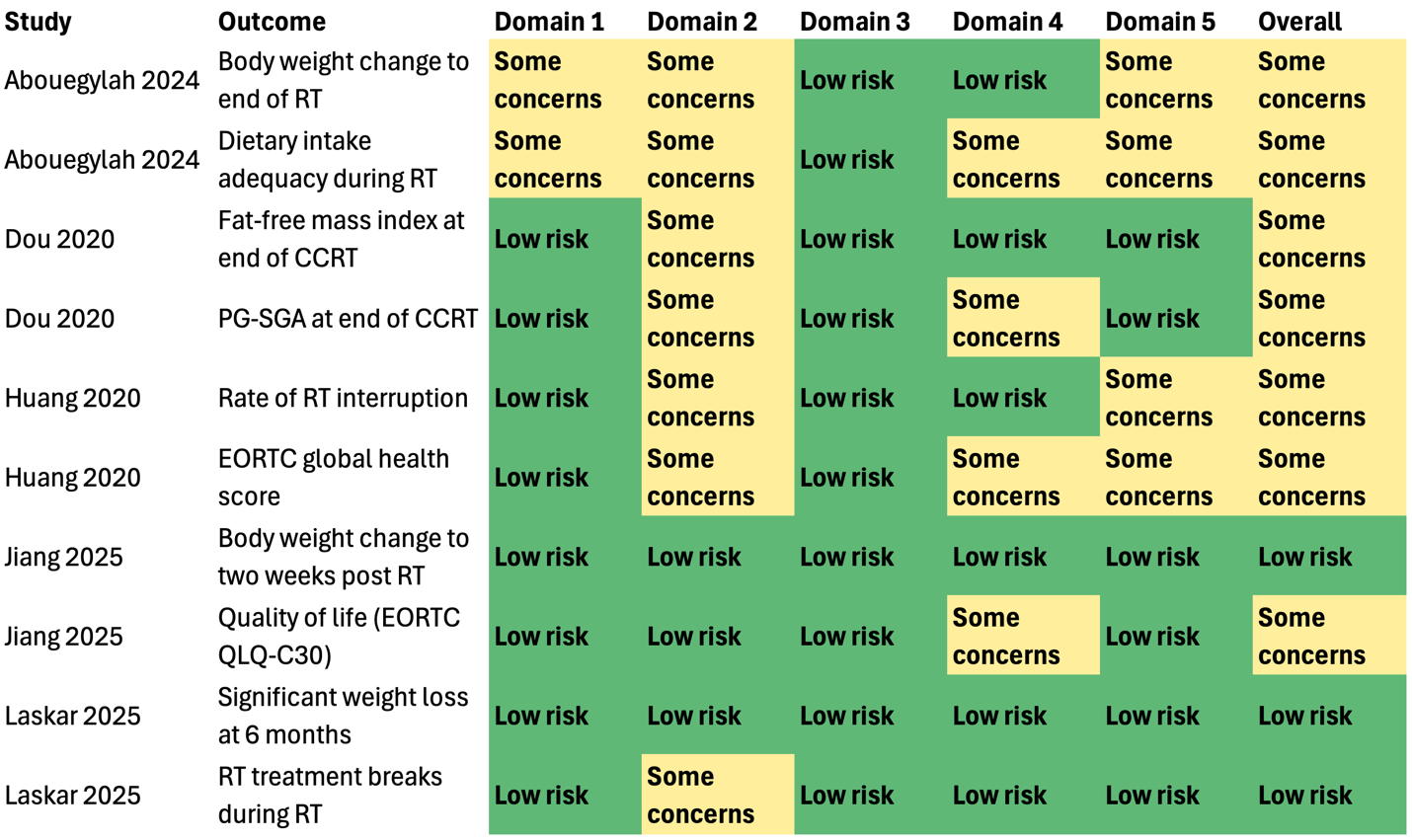
**

**Table S16: Question 3b Quasi-Experimental Studies (ROBINS-I)**

**
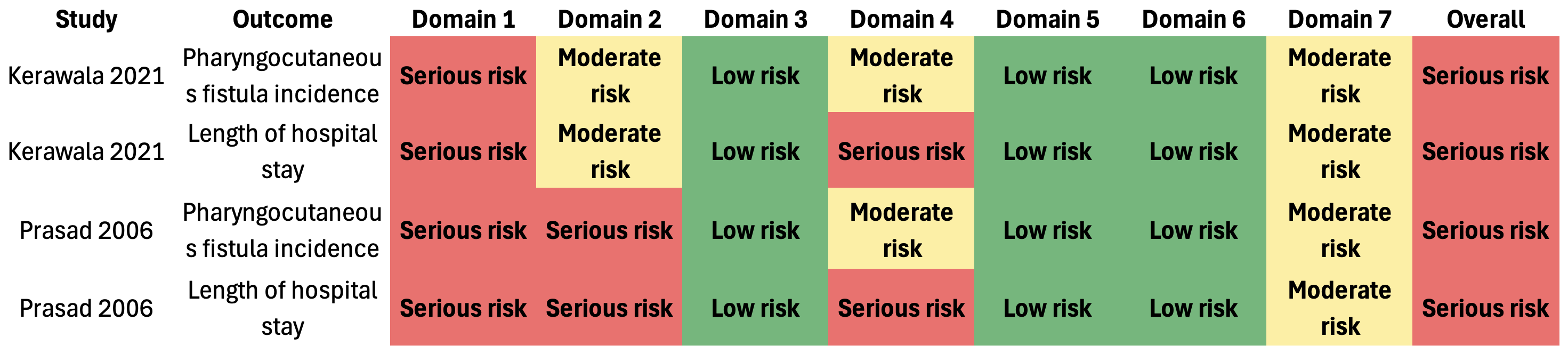
**

**Table S17: Question 5 GRADE Summary of Evidence**

**
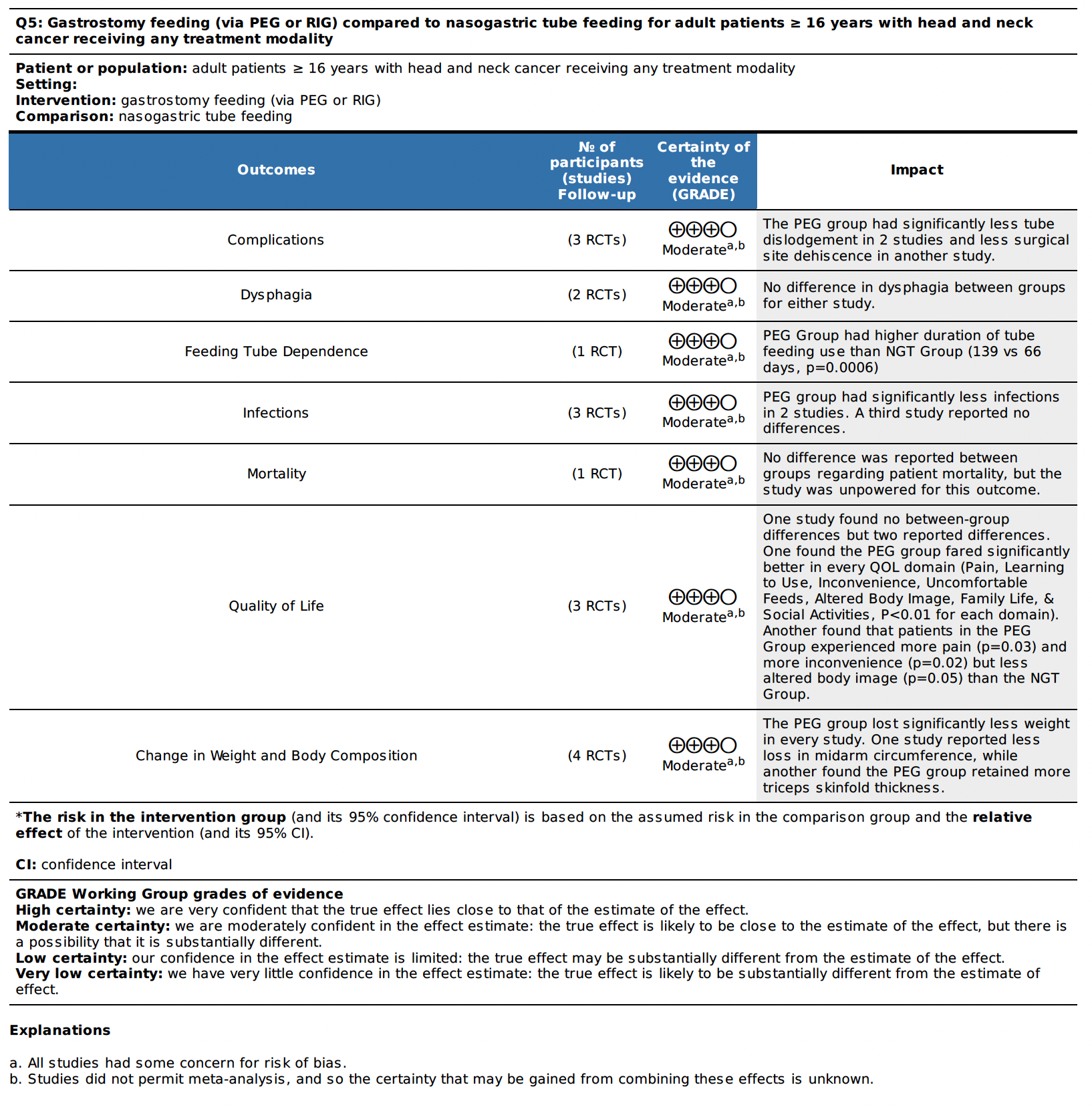
**

**Table S18: Question 5 Randomized Control Trials (ROB2)**

**
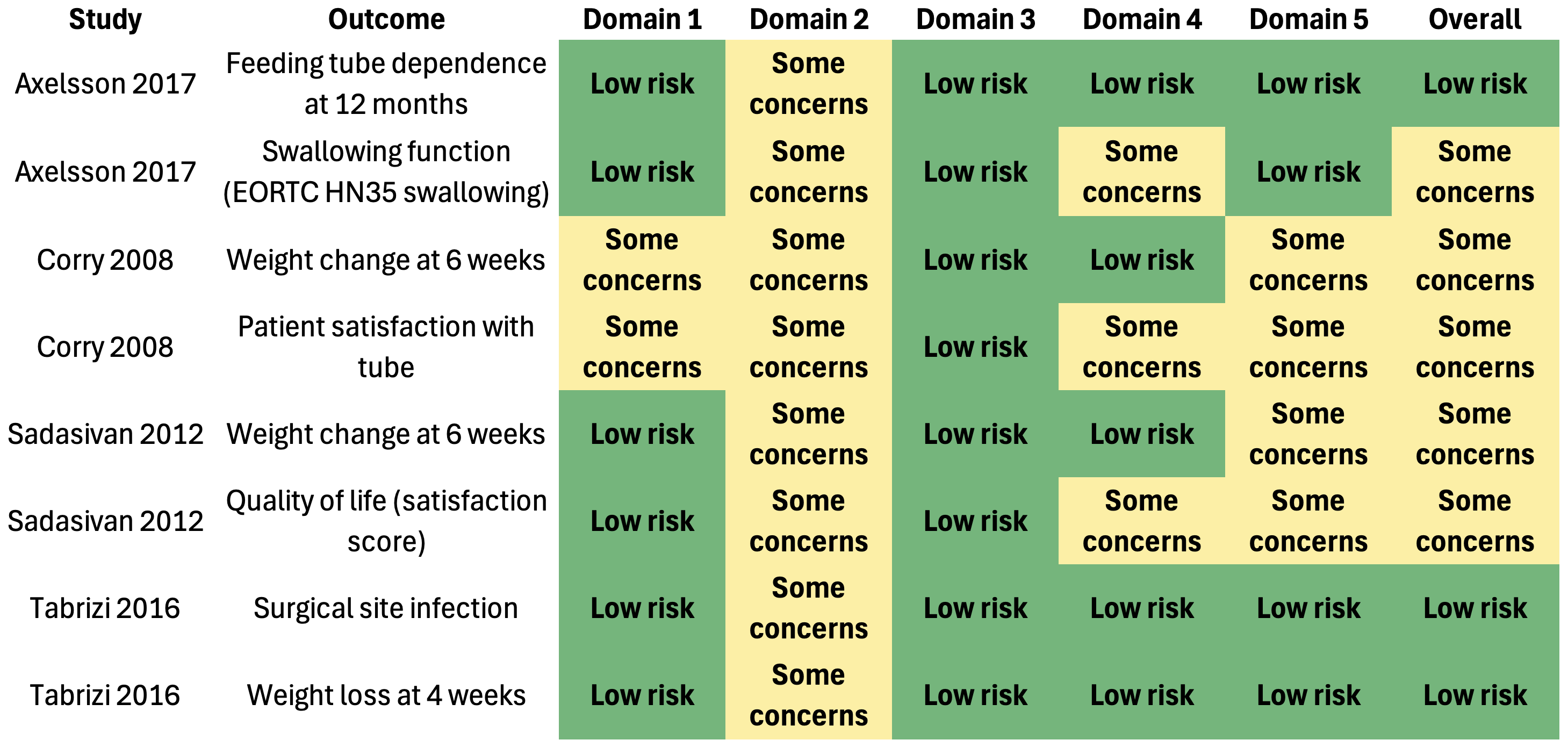
**

**Table S19: Question 6 GRADE Summary of Evidence**

**
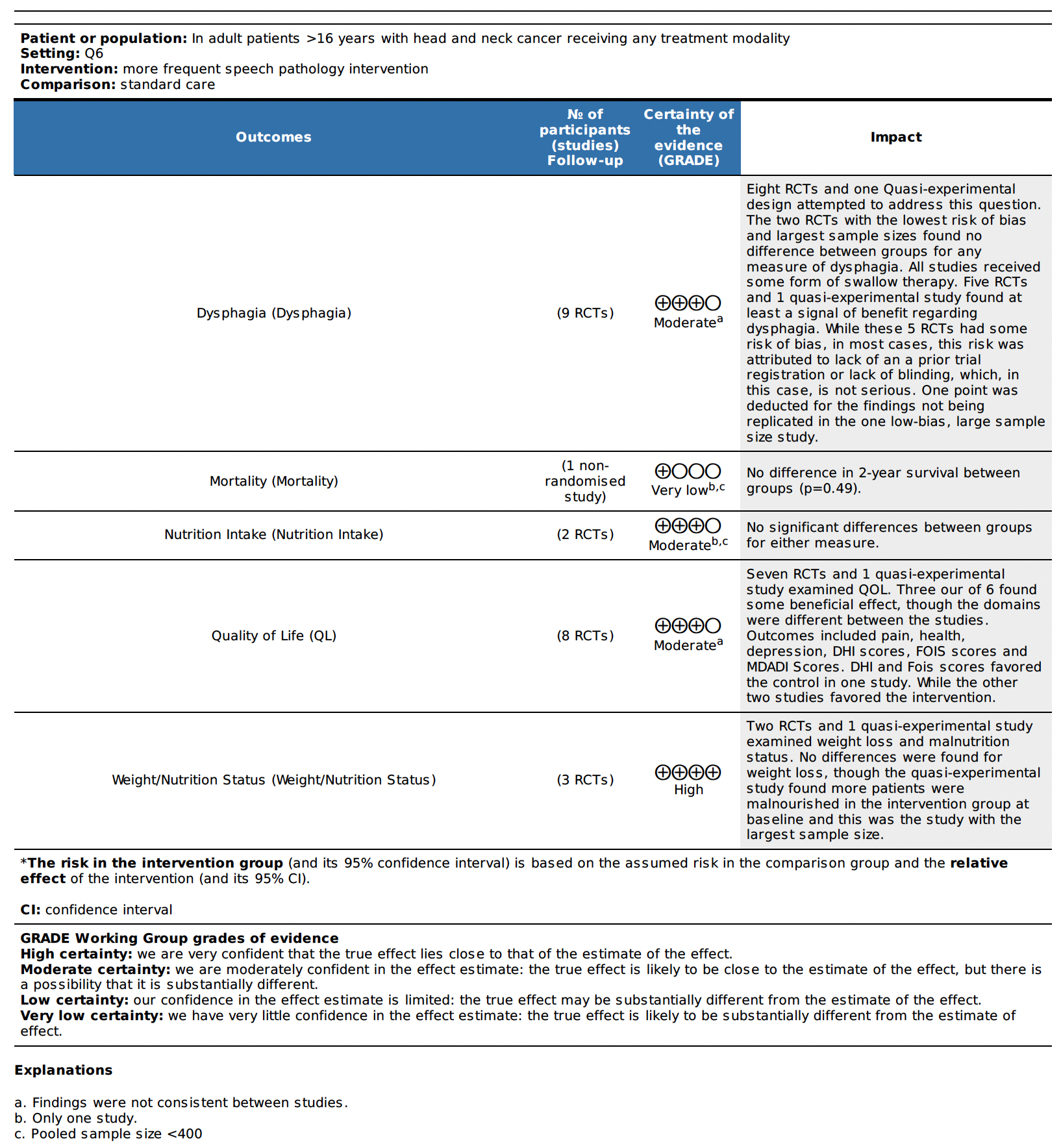
**

**Table S20: Question 6 Randomized Control Trials (ROB2)**

**
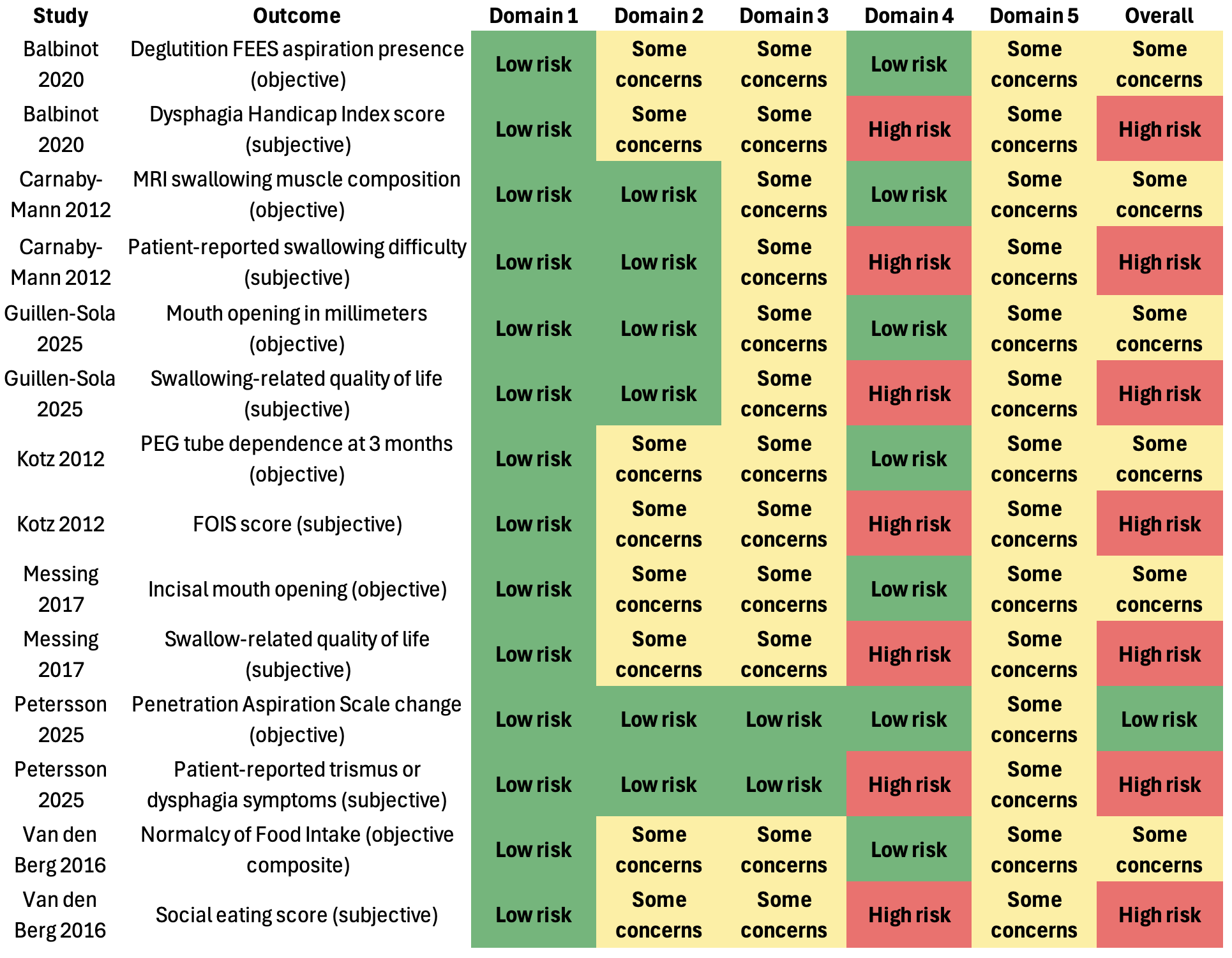
**

**Table S21: Question 6 Quasi-Experimental (ROBINS-I)**

**
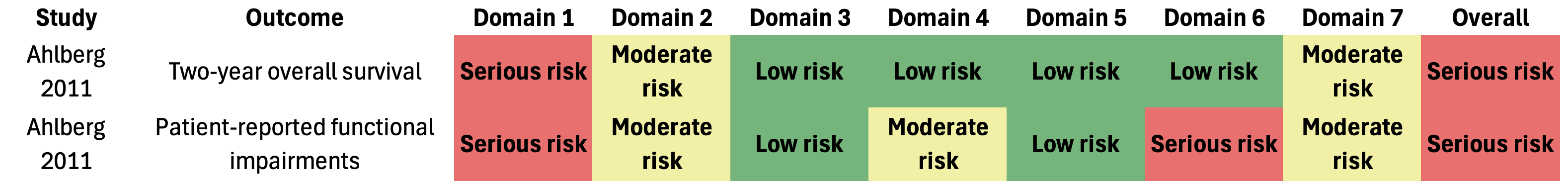
**

**Table S22: Question 7 Summary of Findings**

**
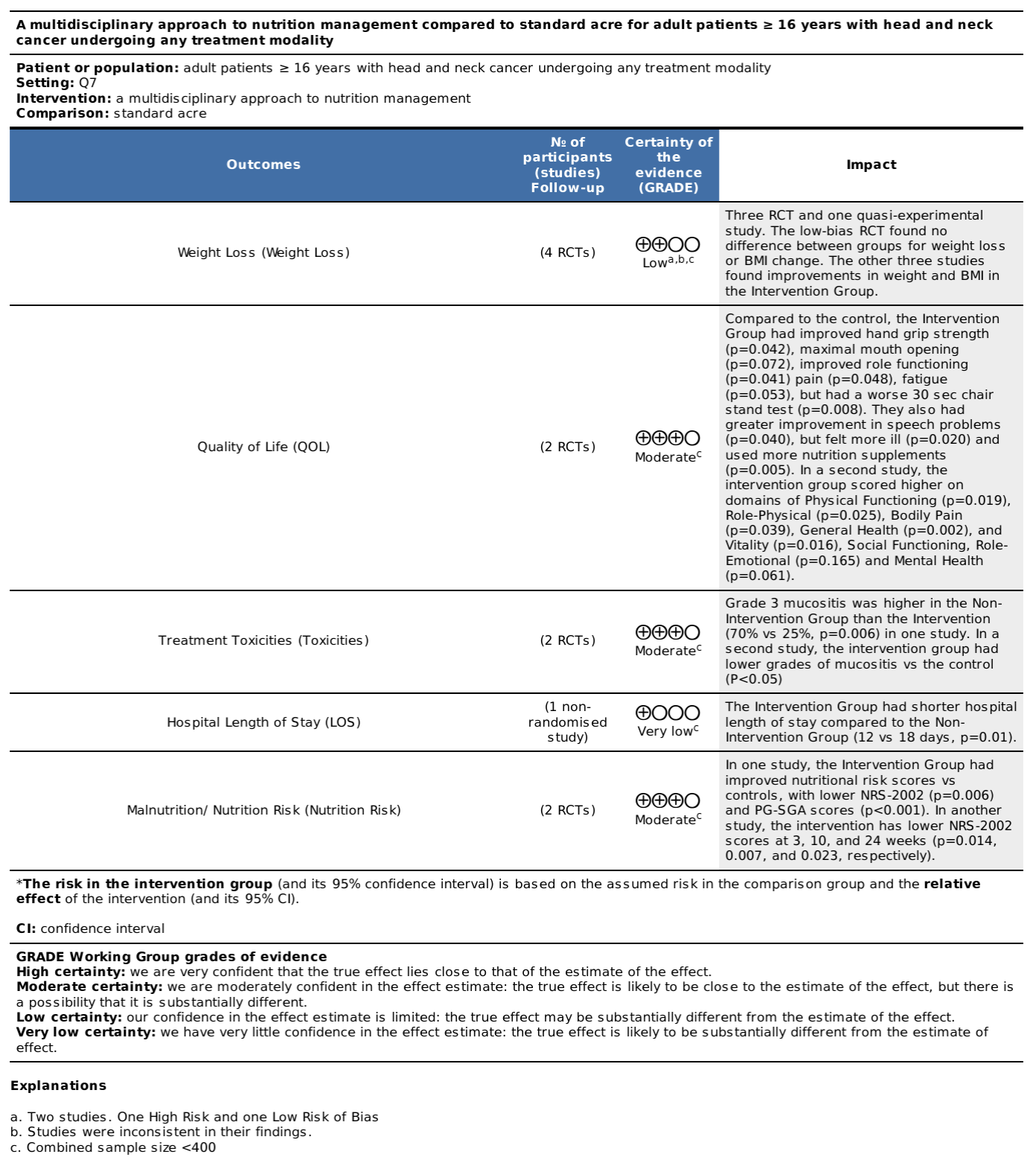
**

**Table S23: Question 7 Randomized Control Trials (ROB2)**

**
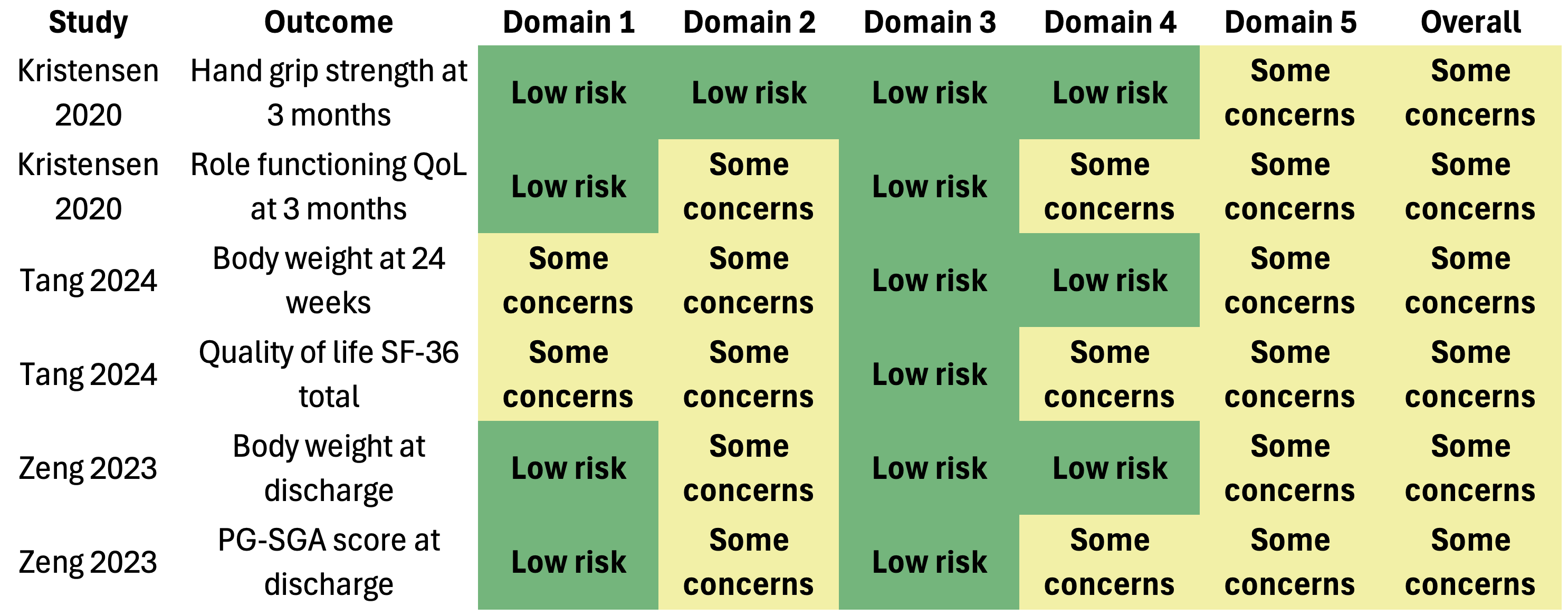
**

**Table S24: Question 7 Quasi-experimental (ROBINS-I)**

**
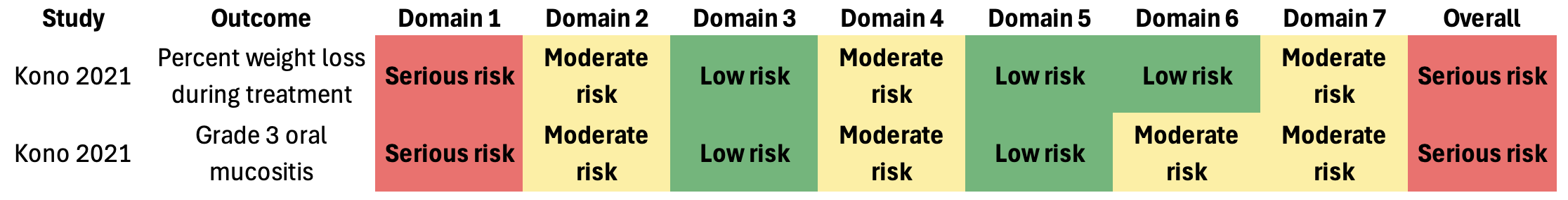
**

**Table S25: Question 8 GRADE Summary of Evidence**

**
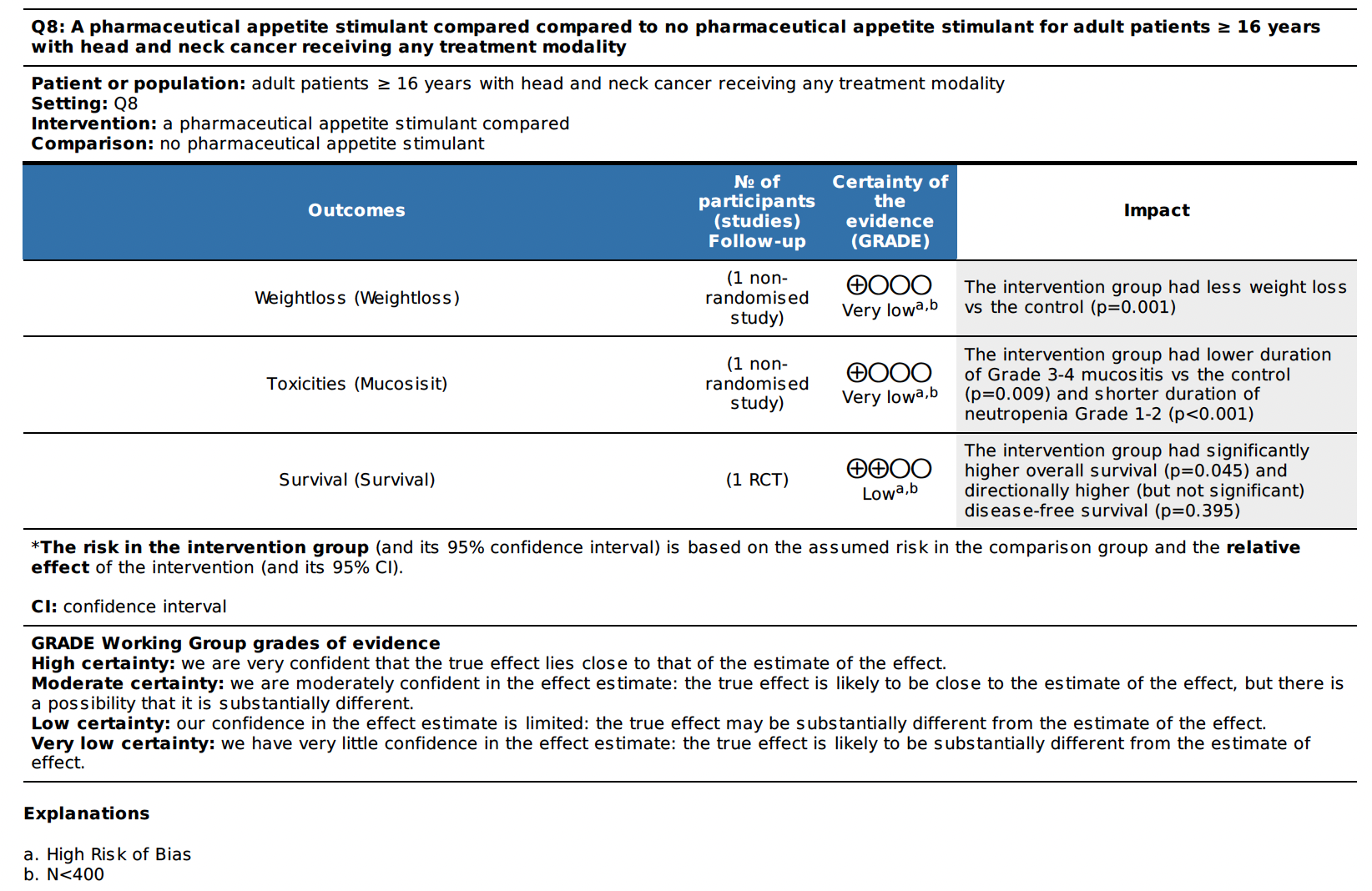
**

**Table S26: Question 8 Quasi-experimental (ROBINS-I)**

**
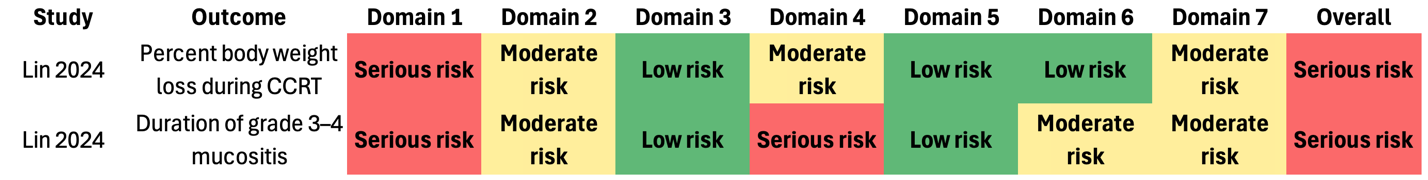
**

**Table S27: Question 10 GRADE Summary of Findings**

**
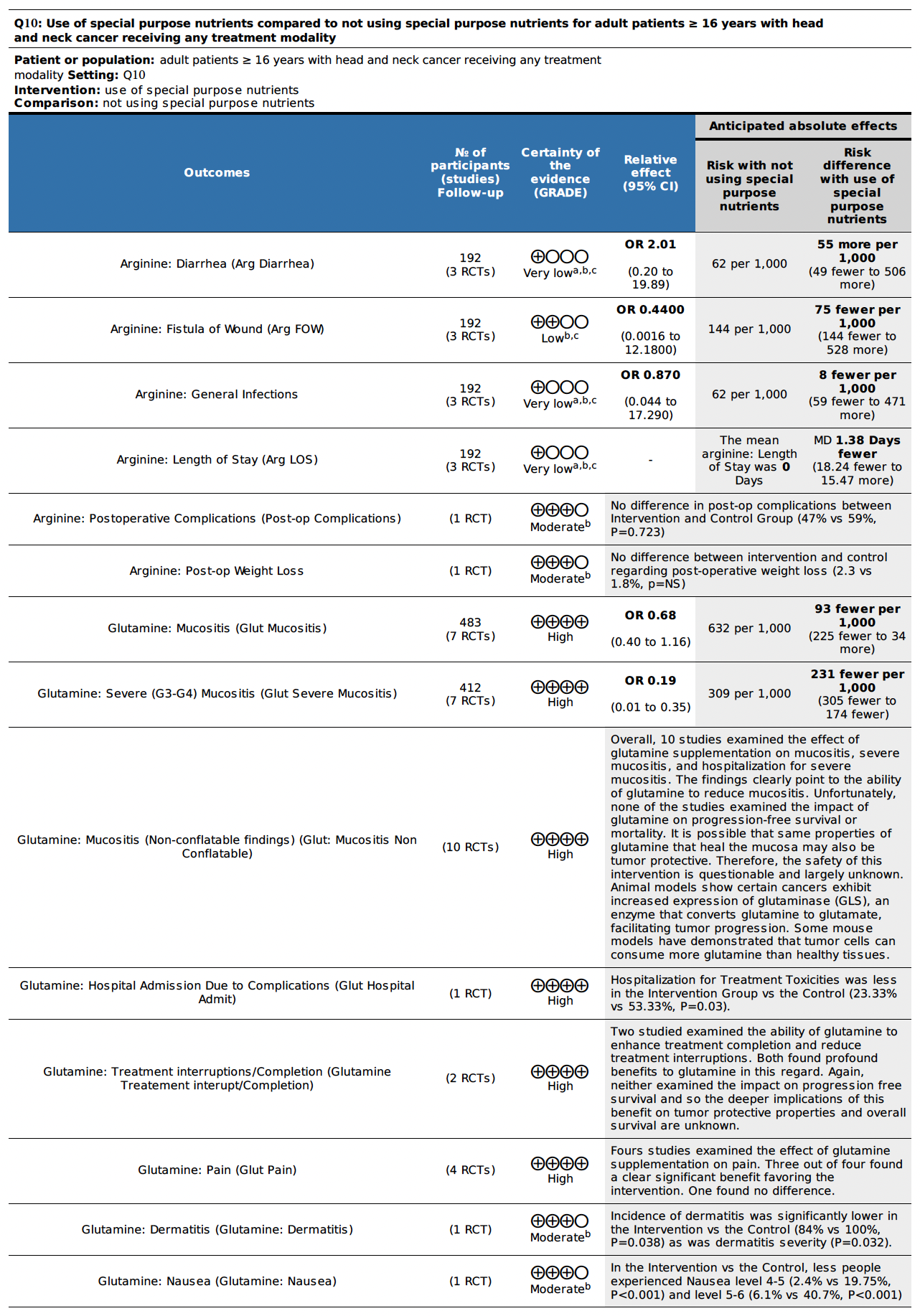
**

**
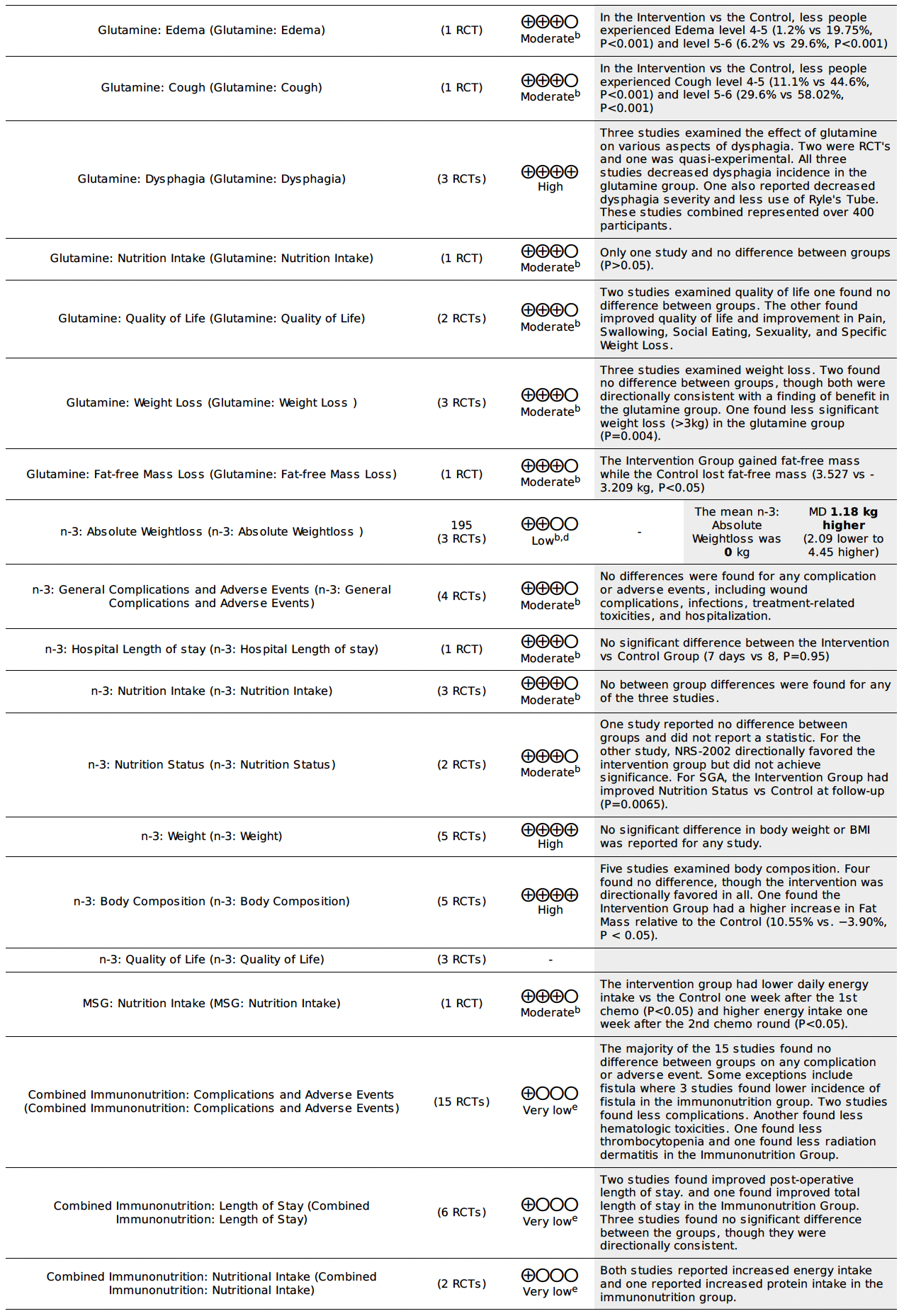
**

**
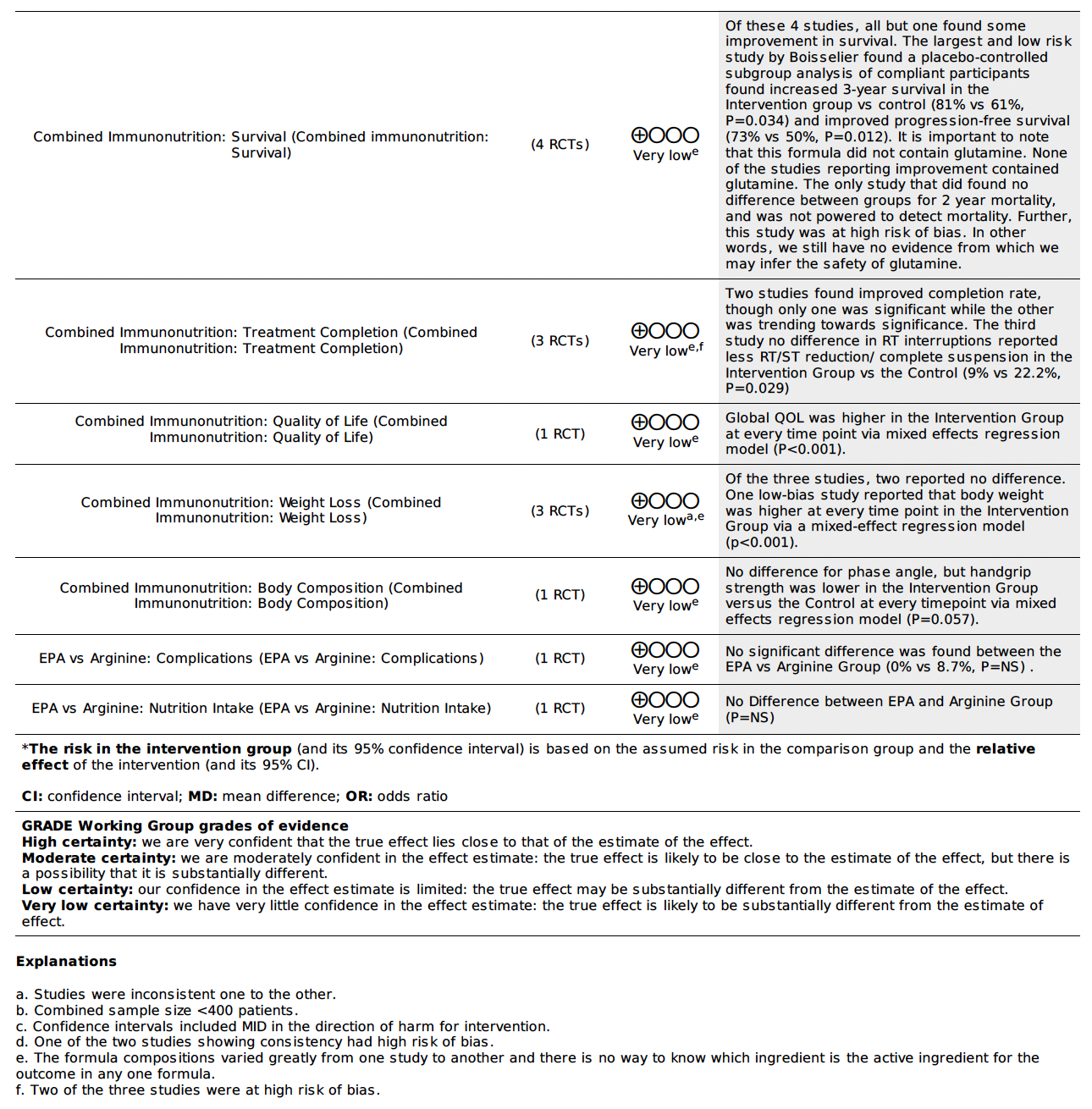
**

**Table S28: Question 10 Randomized Control Trials (ROB2)**

**
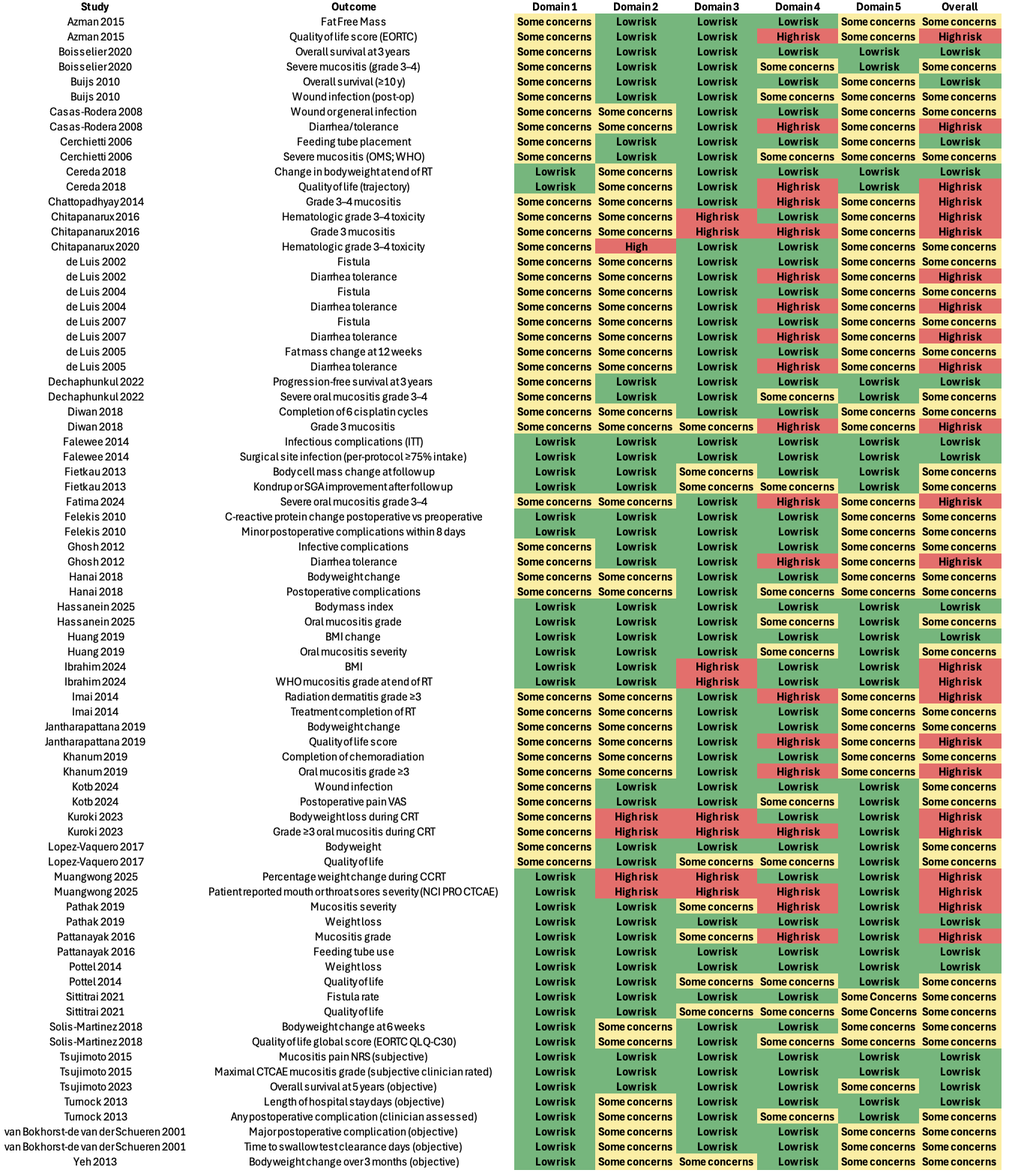
**

**Table S29: Question 10 Quasi-Experimental (ROBINS-I)
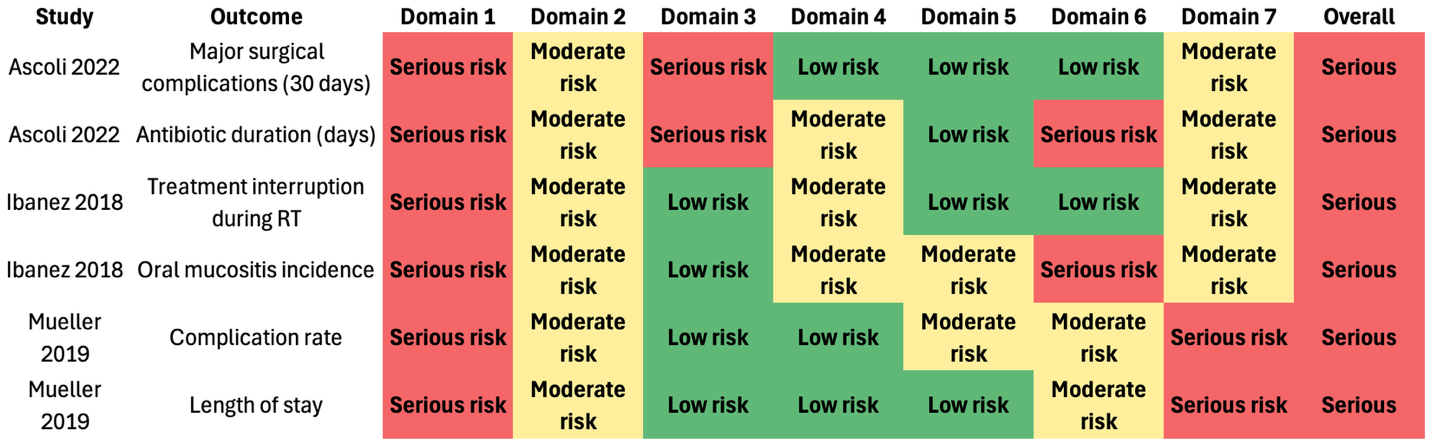
**
